# Moderate-to-severe asthma in individuals of European ancestry: a genome-wide association study

**DOI:** 10.1016/S2213-2600(18)30389-8

**Published:** 2019-01

**Authors:** Nick Shrine, Michael A Portelli, Catherine John, María Soler Artigas, Neil Bennett, Robert Hall, Jon Lewis, Amanda P Henry, Charlotte K Billington, Azaz Ahmad, Richard J Packer, Dominick Shaw, Zara E K Pogson, Andrew Fogarty, Tricia M McKeever, Amisha Singapuri, Liam G Heaney, Adel H Mansur, Rekha Chaudhuri, Neil C Thomson, John W Holloway, Gabrielle A Lockett, Peter H Howarth, Ratko Djukanovic, Jenny Hankinson, Robert Niven, Angela Simpson, Kian Fan Chung, Peter J Sterk, John D Blakey, Ian M Adcock, Sile Hu, Yike Guo, Maen Obeidat, Don D Sin, Maarten van den Berge, David C Nickle, Yohan Bossé, Martin D Tobin, Ian P Hall, Christopher E Brightling, Louise V Wain, Ian Sayers

**Affiliations:** a Department of Health Sciences, University of Leicester, Leicester, UK; b Institute for Lung Health, Department of Infection, Immunity and Inflammation, University of Leicester, Leicester, UK; c National Institute for Health Research, Leicester Respiratory Biomedical Research Centre, University of Leicester, Leicester, UK; d Glenfield Hospital, Leicester, UK; e Division of Respiratory Medicine, National Institute for Health Research, Nottingham Biomedical Research Centre, University of Nottingham, Nottingham, UK; f Division of Epidemiology and Public Health, University of Nottingham, Nottingham, UK; g Centre for Infection and Immunity, Queen's University of Belfast, Belfast, UK; h Respiratory Medicine, Birmingham Heartlands Hospital and University of Birmingham, Birmingham, UK; i Institute of Infection, Immunity and Inflammation, University of Glasgow, Glasgow, UK; j Human Development and Health, Clinical and Experimental Sciences, Faculty of Medicine and National Institute of Health Biomedical Research Centre, Southampton, University of Southampton, Southampton, UK; k Division of Infection Immunity and Respiratory Medicine, The University of Manchester, Manchester Academic Health Science Centre, and Manchester University NHS Foundation Trust, Manchester, UK; l The National Heart and Lung Institute, Imperial College, London, UK; m Data Science Institute, Imperial College, London, UK; n Academic Medical Centre, University of Amsterdam, Amsterdam, Netherlands; o Respiratory Medicine, Sir Charles Gairdner Hospital, Perth, WA, Australia; p The University of British Columbia Center for Heart Lung Innovation, St Paul's Hospital Vancouver, Vancouver, BC, Canada; q Division of Respiratory Medicine, University of British Columbia, Vancouver, BC, Canada; r University of Groningen, University Medical Center Groningen, Department of Pulmonology, Groningen Research Institute for Asthma and COPD Research Institute, Groningen, Netherlands; s Merck & Co Inc, Rahway, NJ, USA; t Institut Universitaire de Cardiologie et de Pneumologie de Québec, Department of Molecular Medicine, Laval University, Quebec City, QC, Canada

## Abstract

**Background:**

Few genetic studies that focus on moderate-to-severe asthma exist. We aimed to identity novel genetic variants associated with moderate-to-severe asthma, see whether previously identified genetic variants for all types of asthma contribute to moderate-to-severe asthma, and provide novel mechanistic insights using expression analyses in patients with asthma.

**Methods:**

In this genome-wide association study, we used a two-stage case-control design. In stage 1, we genotyped patient-level data from two UK cohorts (the Genetics of Asthma Severity and Phenotypes [GASP] initiative and the Unbiased BIOmarkers in PREDiction of respiratory disease outcomes [U-BIOPRED] project) and used data from the UK Biobank to collect patient-level genomic data for cases and controls of European ancestry in a 1:5 ratio. Cases were defined as having moderate-to-severe asthma if they were taking appropriate medication or had been diagnosed by a doctor. Controls were defined as not having asthma, rhinitis, eczema, allergy, emphysema, or chronic bronchitis as diagnosed by a doctor. For stage 2, an independent cohort of cases and controls (1:5) was selected from the UK Biobank only, with no overlap with stage 1 samples. In stage 1 we undertook a genome-wide association study of moderate-to-severe asthma, and in stage 2 we followed up independent variants that reached the significance threshold of p less than 1 × 10^−6^ in stage 1. We set genome-wide significance at p less than 5 × 10^−8^. For novel signals, we investigated their effect on all types of asthma (mild, moderate, and severe). For all signals meeting genome-wide significance, we investigated their effect on gene expression in patients with asthma and controls.

**Findings:**

We included 5135 cases and 25 675 controls for stage 1, and 5414 cases and 21 471 controls for stage 2. We identified 24 genome-wide significant signals of association with moderate-to-severe asthma, including several signals in innate or adaptive immune-response genes. Three novel signals were identified: rs10905284 in *GATA3* (coded allele A, odds ratio [OR] 0·90, 95% CI 0·88–0·93; p=1·76 × 10^−10^), rs11603634 in the *MUC5AC* region (coded allele G, OR 1·09, 1·06–1·12; p=2·32 × 10^−8^), and rs560026225 near *KIAA1109* (coded allele GATT, OR 1·12, 1·08–1·16; p=3·06 × 10^−9^). The *MUC5AC* signal was not associated with asthma when analyses included mild asthma. The rs11603634 G allele was associated with increased expression of *MUC5AC* mRNA in bronchial epithelial brush samples via proxy SNP rs11602802; (p=2·50 × 10^−5^) and *MUC5AC* mRNA was increased in bronchial epithelial samples from patients with severe asthma (in two independent analyses, p=0·039 and p=0·022).

**Interpretation:**

We found substantial shared genetic architecture between mild and moderate-to-severe asthma. We also report for the first time genetic variants associated with the risk of developing moderate-to-severe asthma that regulate mucin production. Finally, we identify candidate causal genes in these loci and provide increased insight into this difficult to treat population.

**Funding:**

Asthma UK, AirPROM, U-BIOPRED, UK Medical Research Council, and Rosetrees Trust.

## Introduction

Asthma is a common disease and was identified as the most prevalent chronic respiratory disease in the Global Burden of Diseases, Injuries, and Risk Factors Study (GBD) 2015.^1^, ^2^ 10–15% of individuals with asthma have severe asthma and substantial unmet clinical needs, with symptoms including debilitating breathlessness, associated frequent exacerbations, and increased hospital admissions despite the high use of medicines.^3^ Both genetic and environmental factors contribute to disease risk, with genetic factors thought to account for 35–95% of the susceptibility to develop asthma.^4^ Previous genome-wide association studies^5^, ^6^, ^7^, ^8^, ^9^, ^10^, ^11^, ^12^, ^13^, ^14^, ^15^, ^16^, ^17^, ^18^, ^19^, ^20^ have identified 38 regions of association with asthma, including signals in or near *PEX14, IL6R, PYHIN1* (African American individuals only)*, ADAMTS4, CD247, TNFSF18, DENND1B, ADORA1, ID2, IL1RL1/IL18R1, D2HGDH, LPP, TLR1, USP38* (Japanese individuals only), *PDE4D, TSLP/WDR36, RAD50/IL13, NDFIP1, GPX5, HLA-C/NOTCH4/HLA-DRB1/HLA-DQA1, GRM4, BACH2, CDHR3, SLC30A8* (Japanese individuals only)*, ZBTB10, IL33, EQTN, GATA3, LRRC32, IKZF4, STAT6, RAD51B, RORA, SMAD3, CLEC16A, ERBB2/GSDMB/ORMDL3, ZNF652*, and *IL2RB.* Asthma signals substantially overlap with signals reported in genome-wide association studies of self-reported allergy and allergic sensitisation—eg, *TLR1, WDR36, IL1RL1, SMAD3, STAT6, C11orf30* (EMSY)*, IL1RL1*, and *TLR1.*^21^, ^22^ In a 2018 genome-wide association study of allergic disease and asthma,^23^ the authors showed a genetic correlation between asthma and allergic disease with evidence of specific loci being unique to asthma—eg, *ORMDL3*.

**Research in context**

**Evidence before this study**

We searched the National Human Genome Research Institute-European Bioinformatic Institute Catalog of published genome-wide association studies from database inception to January, 2018, for studies that tested the association between genetic variants and asthma using the search term “asthma”, and manually searched the findings to identify studies that used a diagnosis of asthma to define cases. We examined the original publications and included studies with more than 500 cases and 500 controls and we considered signals of relevance to be those that met genome-wide significance (p<5 × 10^−8^). These previous studies reported 38 regions associated, at genome-wide significance, with susceptibility to develop asthma, providing novel insight into disease biology. To date, only two genome-wide association studies have specifically investigated moderate-to-severe asthma; however, the power of these studies was restricted by the number of cases included (<1000).

**Added value of this study**

To our knowledge, this is the largest genetic study of moderate-to-severe asthma to date. We identified three novel genome-wide significant genetic associations that imply *MUC5AC, GATA3*, and *KIAA1109* have an association with susceptibility to the development of moderate-to-severe asthma. Altered expression of the pathogenic mucin *MUC5AC* potentially contributes to mucus plugging and airway obstruction, *GATA3* is a transcription factor linked to the T-cell response in asthma and eosinophilia, and the *KIAA1109* locus has previously been associated with allergic sensitisation. We also describe and further characterise the contribution of 21 previously described asthma signals to this phenotype, and identify potential candidate causal genes.

**Implications of all the available evidence**

Identification of genetic associations with variants in multiple genes of the innate or adaptive immune (type 2 inflammation) pathways suggest that targeting this pathway could be a therapeutic opportunity in moderate-to-severe asthma. The association identified between variants in the *MUC5AC* locus adds to evidence of alterations in the airway epithelium and mucin dysregulation in more severe forms of asthma, and potentially supports specific targeting of *MUC5AC* expression and induction in severe asthma. Identification of the *GATA3* and *KIAA1109* signals further extend the data that genetic variants in these regions are associated with asthma, potentially via eosinophilia and allergic sensitisation, two drivers of asthma that are also important in moderate-to-severe disease.

The concept of shared genetic origins between different allergic diseases has been tested in a large genome-wide association study^24^ with 180 129 cases (asthma, allergic rhinitis, or atopic dermatitis) and 180 709 controls. The study identified 99 genetic susceptibility loci, including 136 independent signals implicating genes involved predominantly in immune function, with only six signals showing some disease specificity—eg, *ORMDL3* specific for asthma.

In the first genome-wide association study in severe or difficult to treat asthma,^25^ which used the Epidemiology and Natural History of Asthma: Outcomes and Treatment Regimes (TENOR) cohort, associations with known asthma loci were found—eg, with single nucleotide polymorphisms (SNPs) in the *RAD50/IL13* and *HLA-DR/HLA-DQ* regions. Similarly, the Asthma UK Genetics of Severe Asthma (AUGOSA) study^19^ of moderate-to-severe asthma replicated the known 17q21 association at *ORMDL3/GSDMB/ZPB2.* Neither study identified any new signals that met genome-wide significance, potentially because the number of cases was small. Therefore, we aimed to complete a large genome-wide association study of moderate-to-severe asthma to address three specific objectives: first, we aimed to identify novel signals predicting disease risk for moderate-to-severe asthma (as opposed to mild asthma); second, we wanted to see whether asthma signals that have been previously described were specifically associated with moderate-to-severe asthma; and finally, we aimed to translate genetic findings into disease mechanisms via initial functional studies using the Unbiased BIOmarkers in PREDiction of Respiratory Disease Outcomes (U-BIOPRED) integrated asthma patient genomics resource,^26^ which might in turn identify new targets for therapeutic intervention.

## Methods

### Study design and participants

In this genome-wide association study, we used a two-stage design to identify novel and significant genome-wide associations that confer susceptibility to moderate-to-severe asthma. We used a two-stage case-control design, with variants that showed suggestive association (p<1 × 10^−6^) in stage 1 tested in stage 2 and then meta-analysed across the two stages to maximise power.

For stage 1, we selected individuals of European ancestry with moderate-to-severe asthma who had been recruited from primary and secondary care settings across the UK as part of the Genetics of Asthma Severity and Phenotypes (GASP) initiative, with additional cases included from the U-BIOPRED asthma cohort^26^ and the UK Biobank May, 2015,^27^, ^28^ genetic data release (appendix). Genotyped data were assessed for quality control (details are in the appendix). From GASP and U-BIOPRED, we identified patients with moderate-to-severe asthma by assessing clinical records that indicated that a patient was taking medication required for patients defined as having moderate-to-severe asthma according to the British Thoracic Society (BTS) 2014 guidelines.^29^ From the UK Biobank, cases of moderate-to-severe asthma were defined as having asthma diagnosed by a doctor, taking medication for asthma, no diagnosis of emphysema or chronic bronchitis by a doctor, and meeting the definition of moderate-to-severe asthma by BTS criteria. Therefore, cases were selected from individuals for whom medication information was available and who met BTS stage 3–5 criteria—ie, for stage 3, taking a long-acting β_2_ agonist plus inhaled corticosteroid; stage 4, taking higher dose inhaled corticosteroids than stage 3 patients, and addition of a fourth drug (eg, leukotriene receptor antagonist, theophylline); and stage 5, taking oral corticosteroid or omalizumab, or both. A complete list of medications used to identify patients with moderate-to-severe asthma is in the appendix. Controls for stage 1 were identified from the UK Biobank by taking the remaining subjects for whom genotyped data were available that passed quality control and excluding individuals with asthma, rhinitis, eczema, allergy, emphysema, or chronic bronchitis as diagnosed by a doctor, or if medication data were not available to assign to either the mild-moderate or moderate-severe asthma group. Additional controls for stage 1 were included from U-BIOPRED to ensure we had controls from each cohort. Patients in the U-BIOPRED cohort had not been screened for rhinitis or eczema, and so this information was not available for these controls at time of selection. For stage 2, both cases and controls were selected from the UK Biobank May, 2017, release using the same criteria to define cases and controls as in stage 1. There was no overlap in the patients included in stage 1 and stage 2. A case-control ratio of 1:5 was chosen for both stages to balance power and computational time. Cases and controls were matched across age and sex strata, and in stage 1 across genotyping arrays. All cohorts included individuals with self-reported European ancestry; individuals of non-European ancestry were excluded to reduce confounding of the study by ancestry.

UK Biobank has ethical approval from the UK National Health Service (NHS) National Research Ethics Service (Ref 11/NW/0382). All other studies were approved by an appropriate ethics committee. Informed consent was obtained from all participants.

### Procedures

In stage 1, cases and controls were genotyped using the Affymetrix Axiom (Affymetrx, Santa Clara, CA, USA) UK BiLEVE array^30^ and the later-generation Affymetrix Axiom UK Biobank array, which are 95% identical in content. In stage 2, only the later-generation Affymetrix Axiom UK Biobank array was used. If the sentinel SNP from stage 1 was not available in stage 2, a proxy was chosen with the highest linkage disequilibrium to the sentinel SNP. Genotyping and imputation procedures are described in the appendix. After imputation using UK10K Project^31^ and 1000 Genomes Project Phase 3^32^ reference panels, 33 771 858 SNPs were available for association testing in stage 1 (appendix).

### Statistical analysis

Descriptive statistics of baseline characteristics were compared using a χ^2^ test to check for imbalances in comorbidities, smoking, and oral corticosteroid use between cases in stage 1 and 2. Our sample sizes were determined by the number of moderate-to-severe cases in each cohort for each stage. We then collected data for an excess of potenial controls and used a ratio of 1:5 cases to controls to maximise the power of our study.

For stage 1, we used a logistic model of association to determine genome-wide associations for susceptibility to moderate-to-severe asthma and assumed an additive genetic model of asthma status with imputed genotype dose fitted (effect allele count continuous over the range 0–2 to reflect uncertainty in genotype imputation) using SNPTEST version 2.5,^33^ adjusted for ancestry using the first ten principal components of genotypic variance derived by EIGENSOFT 6.1.4. In stage 1, we used conditional analyses (Genome-wide Complex Trait Analysis version 1.26.0^34^) to identify additional independent signals in the same loci (passing the same threshold of p<1× 10^−6^ as for the unconditional analysis) and we did a sensitivity analysis to check for array effects. In stage 2, we followed up independent variants that reached the p value significance threshold of less than 1 × 10^−6^ in stage 1 association testing.

Variants with p<1 × 10^−6^ in stage 1 were then meta-analysed across stage 1 and stage 2 using inverse-variance weighted meta-analyses. Variants with the same direction of effect in stages 1 and 2, with a genome-wide significant association (ie, p<5 × 10^−8^) in the meta-analyses of stages 1 and 2 and a p value of less than 0·05 in stage 2, were included in further analyses. We used a Bonferroni correction for the number of putative novel signals as the threshold for independent replication in stage 2. We controlled for age and sex by selecting cases and controls with similar age and sex distributions. Ancestry was controlled by selecting European samples and including ten principal components as covariates. To avoid missing data, we only included samples with complete phenotype, age, sex and covariate data.

Because we excluded patients with allergic disease from the controls in stage 1 and 2, we could potentially have inflated shared genetic signals associated with allergic comorbidities in the asthma population. Hence, we also did a sensitivity analysis that included stage 2 cases and controls including individuals with rhinitis, eczema, and allergy.

To investigate whether novel variants we identified as associated with moderate-to-severe asthma showed an association with susceptibility to all types of asthma (mild, moderate, and severe), we interrogated a large genome-wide association study of asthma,^5^ which included 28 399 cases of self-reported asthma and 128 843 controls. We also investigated SNPs previously associated with asthma^5^, ^6^, ^7^, ^8^, ^9^, ^10^, ^11^, ^12^, ^13^, ^14^, ^15^, ^16^, ^17^, ^18^, ^19^, ^20^ and allergic diseases^23^, ^24^ at genome-wide significance (p<5 × 10^−8^) in our stage 1 dataset.

For all signals we determined to have a genome-wide significant association with moderate-to-severe asthma, we investigated the lead SNP (and SNPs in linkage disequilibrium—ie, r^2^>0·4) for association with mRNA levels in cells and tissues using expression quantitative loci (eQTL) datasets. For lung tissue we used an eQTL database with data for 1110 subjects,^35^ and for blood cells we used an eQTL database with data for 5311 subjects,^36^ and we used a 10% false discovery rate for eQTLs for these two databases. To complement these resources, we used five additional U-BIOPRED eQTL datasets^37^, ^38^, ^39^ for blood (n=345), sputum (n=91), bronchial biopsy samples (n=84), bronchial brushing (n=117), and nasal brushing (n=75), and for these datasets we used a 5% false discovery rate (appendix). For the U-BIOPRED cohort, DNA from whole blood was extracted and genotyped as outlined and after imputation (SHAPEIT2 for pre-phasing and IMPUTE 2, using 1000 Genome Phase 3 as reference panel) and quality control, 2 536 796 SNPs were available for association testing. Transcriptomic analysis was done with the Affymetrix HT HG-U133 1 PM GeneChip (Affymetrix, Santa Clara, Calif) on extracted RNA from the different samples. When the sentinel SNP was not available we used a proxy with the highest linkage disequilibrium. We considered an association signal meeting the designated false discovery rate for any dataset to be of interest.

For novel signals for which an eQTL effect was observed, we investigated the expression of proteins in human lung tissue using Protein Atlas^40^ to identify relevant airway cell types for further study. We also investigated mRNA levels in patients with severe asthma using two gene expression omnibus datasets to see if altered expression is a feature of these patients compared with patients with mild asthma, which might imply a mechanistic role in moderate-to-severe disease, (appendix). GSE43696^41^ includes Agilent Human GE 4×44K V2 Gene Expression data for bronchial epithelial cells from 20 controls, 50 patients with mild asthma, and 38 patients with severe asthma. GSE89809^42^ contains Affymetrix HT HG-U133+ PM GeneChip data for 18 controls, and 14 patients with mild asthma, 13 with moderate asthma, and 11 with severe asthma. The definition of asthma severity was different between these studies. In GSE43696, patients with mild-to-moderate asthma had a predicted forced expiratory volume in 1 s of less than 60%, with or without a low-to-moderate dose of inhaled corticosteroids, and patients with severe asthma were defined as having continuous use of high-dose inhaled corticosteroids or frequent use of oral corticosteroids, or both, with continuing symptoms or chronic airflow limitations. In GSE89809, patients with mild asthma were defined as taking β_2_ agonists alone, those with moderate asthma as taking inhaled corticosteroids, and those with severe asthma had persistent symptoms despite taking high-dose inhaled corticosteroids and oral corticosteroids. Both of these studies defined patients as having moderate-to-severe asthma by use of medication, and so comparable definitions were used between these genome-wide association studies and our study. Robust multi-array average data was extracted from these datasets for *KIAA1109* and *MUC5AC* and expression was compared across groups (Kruskal-Wallace test).

To provide insight into the disease mechanism, we investigated lead SNPs (and SNPs in linkage disequilibrium with r^2^>0·4) at the novel loci using the HaploReg version 4 resource^43^ and the deep-learning functional prediction resource DeepSEA.^44^ We used GRASP^45^ and a GWAS catalog^46^ to identify whether any variants in linkage disequilibrium with the three novel signals had previously been reported in genome-wide association studies of other diseases or quantitative outcomes.

### Role of the funding source

The funder of the study had no role in study design, data collection, data analysis, data interpretation, or writing of the report. IS and LVW had full access to all the data in the study and had final responsibility for the decision to submit for publication.

## Results

**Figure 1:**
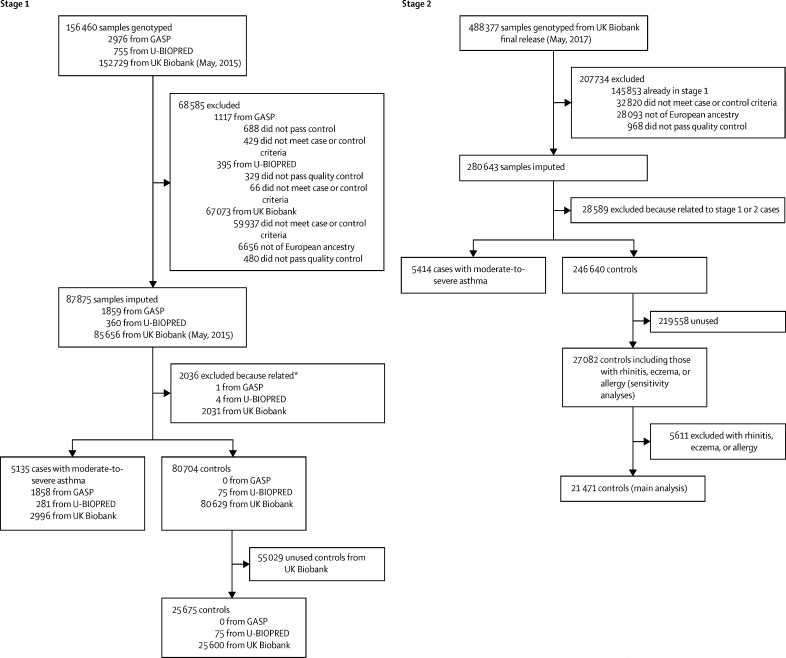
Quality control and sample selection GASP=Genetics of Asthma Severity and Phenotypes. U-BIOPRED=Unbiased BIOmarkers in PREDiction of respiratory disease outcomes. *Related samples (second degree or closer) were removed; see appendix for more details of sample selection.

For the stage 1 analyses, after genotyping of the GASP and U-BIOPRED cohorts and using available data from the UK BioBank (May, 2015, release), data were available for 5135 moderate-to-severe asthma cases, (GASP n=1858, U-BIOPRED n=281, and UK Biobank n=2996), and 25 675 controls (U-BIOPRED n=75, and UK Biobank n=25 600; figure 1). For the independent stage 2 cohort, 5414 cases and 21 471 controls were selected from the UK Biobank (May, 2017, release) after excluding the cases and controls in stage 1. Baseline characteristics for the stage 1 and 2 cohorts are in table 1.

**Table 1:** Baseline characteristics of stage 1 and stage 2 cohorts

		**Stage 1 cohort**		**Stage 2 cohort**		
		Cases (n=5135)	Controls (n=25 675)	Cases (n=5414)	Controls (n=21 471)	Controls for sensitivity analyses*(n=27 082)
Age, years		55 (12)	56 (8)	58 (8)	58 (8)	58 (8)
Sex						
	Female	3170 (61·7%)	14 626 (57·0%)	3354 (62·0%)	13 135 (61·2%)	16 816 (62·1%)
	Male	1965 (38·3%)	11 049 (43·0%)	2060 (38·0%)	8336 (38·8%)	10 266 (37·9%)
	FEV_1_, % predicted	72·4% (21·4)	91·8% (17·4)	84·5% (17·2)	93·7% (14·1)	93·9% (13·9)
	FEV_1_/FVC	0·67 (0·12)	0·76 (0·06)	0·73 (0·09)	0·77 (0·06)	0·77 (0·06)
Smoking status						
	Ever smoker	2265 (44·1%)	11 913 (46·4%)	2509 (46·3%)	9479 (44·2%)	11 707 (43·2%)
	Never smoker	2647 (51·6%)	13 487 (52·5%)	2787 (51·5%)	11 621 (54·1%)	14 918 (55·1%)
	Unknown	223 (4·3%)	275 (1·1%)	118 (2·2%)	371 (1·7%)	457 (1·7%)
Rhinitis or eczema status						
	Yes	1897 (36·9%)	8†	2556 (47·2%)	0	5541 (20·5%)
	No	2062 (40·2%)	25 667†	2858 (52·8%)	21 471 (100%)	21 541 (79·5%)
	Unknown	1176 (22·9%)	0†	0	0	0
	Oral corticosteroid use (prednisolone)	222/3710 (6·0%)	NA	162/5414 (3·0%)	NA	NA

Data are mean (SD) or n (%), unless otherwise stated. FEV_1_=forced expiratory volume in 1 s. FVC=forced vital capacity. NA=not applicable. U-BIOPRED=Unbiased BIOmarkers in PREDiction of respiratory disease outcomes.

* Including all controls with rhinitis, eczema, and allergy.

† Patients in the U-BIOPRED cohort were not screened for rhinitis or eczema before sample selection but were subsequently found to comprise eight patients with rhinitis, eczema, or allergy.

Cases in the stage 1 cohort had lower lung function and higher use of oral corticosteroids than cases in stage 2, suggesting a higher severity of asthma among the stage 1 cases. Cases in the stage 1 and 2 cohorts did not differ significantly by the proportion who had allergic comorbidities (allergic rhinitis or eczema, or both; p=0·51) or a history of smoking (p=0·21).

**Figure 2:**
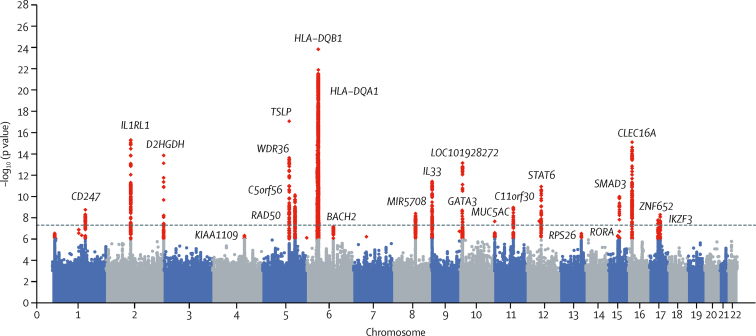
Manhattan plot for stage 1 analyses of risk of moderate-to-severe asthma Data are for 5135 cases with moderate-to-severe asthma and 25 675 controls assessed for 33·8 million well-imputed variants. p values have had genomic control applied. Red data points are signals meeting criteria for follow-up in stage 2 (p<1 × 10^−6^) and the dotted line indicates genome-wide significance (p<5 × 10^−8^). Loci are labelled with the nearest gene for the 24 signals meeting genome-wide significance in the meta-analysis. Quantile-quantile plot for this analysis is in the appendix.

In stage 1, 32 independent signals were associated (ie, p<1 × 10^−6^) with susceptibility to moderate-to-severe asthma, of which 21 additionally met genome-wide significance (p<5 × 10^−8^) in stage 1 alone (figure 2; appendix). Array sensitivity analyses did not identify any array effects (appendix). The 32 signals included independent secondary signals at the *TSLP, IL13*/*RAD50*, and *HLA* loci (appendix).

All 32 signals showing association in stage 1, including 11 potentially novel signals (appendix), were further analysed in the stage 2 cohort dataset. In stage 2 analyses, 26 signals showed consistent direction of effect and p values of less than 0·05 for association (appendix).

After meta-analyses of data from stages 1 and 2, we identified 25 signals that showed consistent direction of effect and overall genome-wide significance. After sensitivity analyses in stage 2, the rs61816761 (Filaggrin, FLG) signal was excluded because the signal was at least in part driven by atopy (appendix).

**Figure 3:**
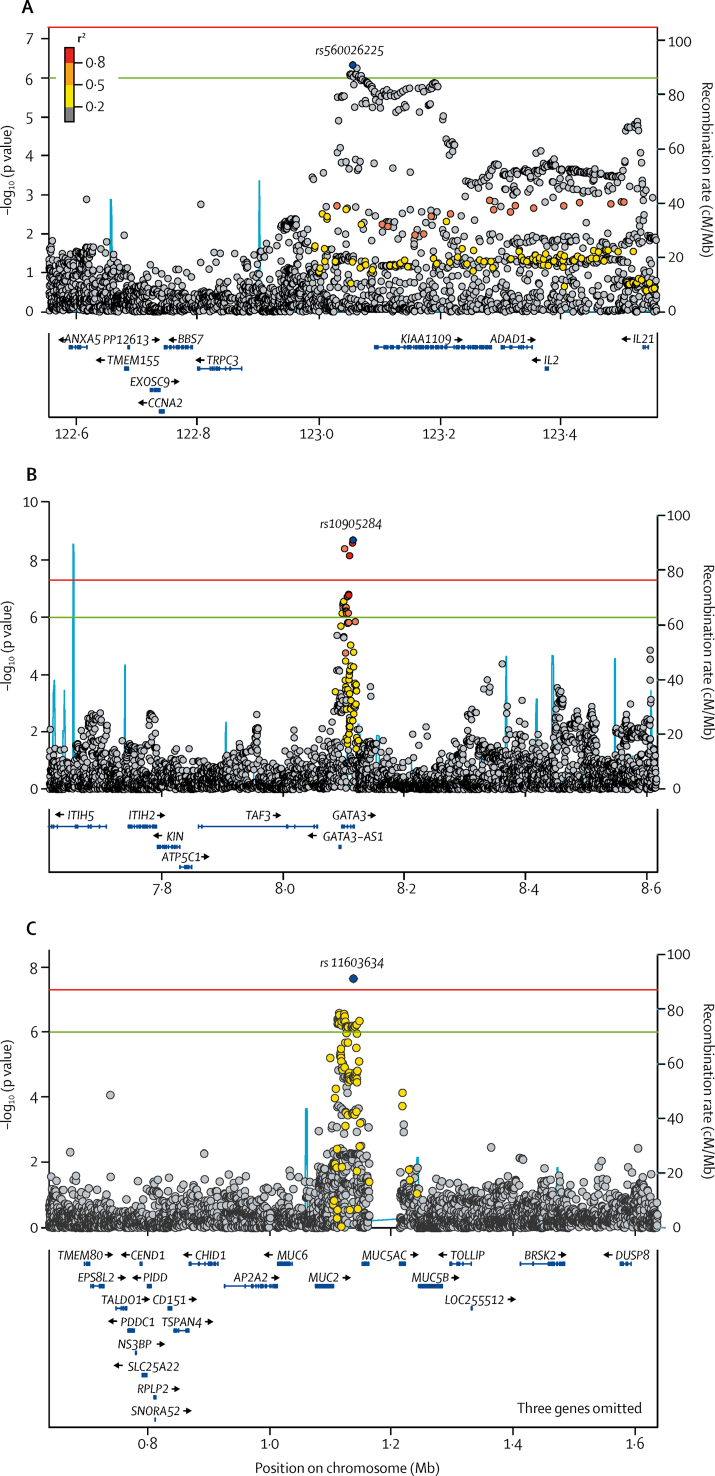
Regional association plots of novel signals *KIAA1109* (A), *GATA3* (B), and *MUC5AC* (C) associated with moderate-to-severe asthma Regional association plots from stage 1 analyses for the three novel signals that show statistically replicated association in stages 1 and 2 and met genome-wide significance in the meta-analyses. Significance of each single nucleotide polymorphism (SNP) is on the –log10 scale as a function of chromosome position (NCBI build 37). The sentinel SNP at each locus is shown by the blue peak, and data points are colour coded to show the correlations (r^2^) of each of the surrounding SNPs to the sentinel SNP. The green line indicates signals meeting criteria for inclusion in stage 2 (p<1 × 10^−6^) and the red line indicates genome-wide significance (p<5 × 10^−8^).

Of the 24 signals, after exclusion of FLG (table 2), three were novel for asthma (figure 3): the sentinel SNP rs10905284 in *GATA3* (coded allele A, odds ratio [OR] 0·90, 95% CI 0·88–0·93; p=1·76 × 10^−10^) and rs11603634 in the *MUC5AC* region (coded allele G, OR 1·09, 1·06–1·12; p=2·32 × 10^−8^). The third signal included an indel (rs560026225, proxy in stage 2 rs72687036) in a locus covering *KIAA1109* (coded allele GATT, OR 1·12, 1·08–1·16; p=3·06 × 10^−9^). rs10905284 (*GATA3*) is a novel signal for asthma independent from previously described signals in the *GATA3* region for asthma—eg, rs10508372,^15^ rs2589561,^11^ and rs12413578.^5^ Confirmed by conditional analyses, we identified a second signal in the *GATA3* region, rs61840192 (labelled *LOC101918272* because it is distal from *GATA3*; table 2), which is in linkage disequilibrium with the previously described signals at rs12413578 (r^2^=0·166) and rs2589561 (r^2^=0·161). Both the *KIAA1109* and *GATA3* signals meet the threshold for independent replication based on Bonferroni correction threshold (p<0·005, Bonferroni correction for 11 potentially novel signals); however, the *MUC5AC* signal did not meet this threshold but was nominally significant (p=0·018; table 2)

**Table 2:** Gene variants with genome-wide significance for moderate-to-severe asthma, by chromosome

	**Position**	**Variant**	**Locus**	**Non-coded**	**Coded**	**Minor allele**	**MAF**	**rsid.ukb (proxy r^2^)**	**Stage 1 cohort**		**Stage 2 cohort**		**Meta-analyses of stage 1 and 2**	
									OR (95% CI)	p value	OR (95% CI)	p value	OR (95% CI)	p value
**Novel**														
4	123 055 701	rs560026225	*KIAA1109*	G	GATT	GATT	23·60%	rs72687036 (0·66)	1·15 (1·09–1·21)	4·62 × 10^−7^	1·09 (1·04–1·15)	7·50 × 10^−4^	1·12 (1·08–1·16)	3·06 × 10^−9^
10	8 115 362	rs10905284	*GATA3*	C	A	C	42·94%	rs10905284	0·87 (0·84–0·91)	2·01 × 10^−9^	0·94 (0·90–0·98)	2·25 × 10^−3^	0·90 (0·88–0·93)	1·76 × 10^−10^
11	1 136 478	rs11603634	*MUC5AC*	A	G	A	49·64%	rs11603634	1·13 (1·08–1·18)	2·30 × 10^−8^	1·05 (1·01–1·10)	1·82 × 10^−2^	1·09 (1·06–1·12)	2·32 × 10^−8^
**Previous**														
1	167 427 247	rs7523907	*CD247*	C	T	C	45·92%	rs7523907	1·14 (1·10–1·20)	1·64 × 10^−9^	1·05 (1·00–1·10)	2·11 × 10^−2^	1·10 (1·06–1·13)	4·82 × 10^−9^
2	102 949 161	rs12479210	*IL1RL1*	C	T	T	38·73%	rs12479210	1·20 (1·15–1·26)	4·77 × 10^−16^	1·19 (1·14–1·24)	4·82 × 10^−15^	1·19 (1·16–1·23)	1·57 × 10^−29^
2	242 698 640	rs34290285	*D2HGDH*	G	A	A	25·74%	rs34290285	0·82 (0·78–0·87)	1·41 × 10^−14^	0·85 (0·81–0·89)	1·16 × 10^−10^	0·84 (0·81–0·87)	2·24 × 10^−23^
5	110 401 872	rs1837253	*TSLP*	T	C	T	25·84%	rs1837253	1·24 (1·18–1·30)	8·49 × 10^−18^	1·14 (1·08–1·20)	1·75 × 10^−7^	1·19 (1·15–1·23)	1·95 × 1^−22^
5	110 467 499	rs1438673	*WDR36*	C	T	T	49·22%	rs1438673	0·87 (0·84–0·91)	2·35 × 10^−9^	0·91 (0·87–0·95)	1·33 × 10^−5^	0·89 (0·86–0·92)	3·29 × 10^−13^
5	131 799 626	rs3749833	*C5orf56*	T	C	C	26·08%	rs3749833	1·17 (1·12–1·23)	1·14 × 10^−10^	1·11 (1·06–1·16)	2·69 × 10^−5^	1·14 (1·10–1·18)	5·60 × 10^−14^
5	131 887 986	rs1986009	*RAD50*	C	A	A	18·71%	rs1986009	1·18 (1·11–1·24)	1·39 × 10^−8^	1·16 (1·10–1·23)	4·11 × 10^−8^	1·17 (1·13–1·22)	2·43 × 10^−15^
6	32 581 739	rs776111176	*HLA-DQA1*	A	AAT	A	14·85%	rs3997872 (0·82)	0·82 (0·79–0·88)	1·81 × 10^−8^	0·85 (0·81–0·90)	2·62 × 10^−9^	0·84 (0·81–0·88)	2·61 × 10^−16^
6	32 627 250	rs9273410	*HLA-DQB1*	C	A	C	44·70%	rs9273410	1·26 (1·20–1·32)	1·07 × 10^−24^	1·16 (1·11–1·21)	2·14 × 10^−10^	1·21 (1·17–1·25)	5·62 × 10^−32^
6	91 001 332	rs367983479	*BACH2*	CA	C	C	38·50%	rs1504215 (0·89)	0·88 (0·85–0·93)	7·12 × 10^−8^	0·92 (0·88–0·96)	1·12 × 10^−4^	0·90 (0·87–0·93)	6·30 × 10^−11^
8	81 266 924	rs71266076	*MIR5708*	C	CT	C	36·93%	rs13274067 (0·97)	0·87 (0·83–0·91)	4·21 × 10^−9^	0·91 (0·87–0·95)	1·57 × 10^−5^	0·89 (0·86–0·92)	6·53 × 10^−13^
9	6 208 030	rs144829310	*IL33*	G	T	T	16·40%	rs144829310	1·23 (1·16–1·30)	3·68 × 10^−12^	1·19 (1·13–1·26)	1·12 × 10^−9^	1·21 (1·16–1·26)	2·29 × 10^−20^
10	9 043 404	rs61840192	*LOC101928272*	G	A	A	42·70%	rs1775555 (0·98)	0·85 (0·81–0·88)	8·42 × 10^−14^	0·86 (0·82–0·89)	1·14 × 10^−12^	0·85 (0·83–0·88)	8·33 × 10^−25^
11	76 293 726	rs7936312	*C11orf30*	G	T	T	47·42%	rs7936312	1·14 (1·10–1·19)	1·09 × 10^−9^	1·19 (1·14–1·24)	3·38 × 10^−16^	1·17 (1·13–1·20)	6·18 × 10^−24^
12	56 449 875	rs7305461	*RPS26*	A	C	A	44·61%	rs1131017 (0·98)	0·88 (0·84–0·92)	1·65 × 10^−8^	0·94 (0·90–0·98)	2·51 × 10^−3^	0·91 (0·88–0·94)	1·01 × 10^−9^
12	57 497 005	rs703816	*STAT6*	T	C	C	43·41%	rs703816	1·16 (1·11–1·21)	1·18 × 10^−11^	1·08 (1·03–1·13)	4·31 × 10^−4^	1·12 (1·09–1·15)	3·69 × 10^−13^
15	61 068 704	rs10519068	*RORA*	G	A	A	12·75%	rs10519068	0·85 (0·79–0·90)	4·81 × 10^−7^	0·85 (0·79–0·90)	5·76 × 10^−7^	0·85 (0·81–0·89)	1·84 × 10^−12^
15	67 441 750	rs72743461	*SMAD3*	C	A	A	23·60%	rs72743461	1·18 (1·12–1·24)	1·03 × 10^−10^	1·11 (1·06–1·17)	2·35 × 10^−5^	1·14 (1·11–1·19)	4·52 × 10^−14^
16	11 230 703	rs7203459	*CLEC16A*	T	C	C	24·56%	rs7203459	0·81 (0·77–0·86)	7·83 × 10^−16^	0·90 (0·85–0·94)	2·33 × 10^−5^	0·86 (0·83–0·89)	4·37 × 10^−18^
17	37 910 368	rs2941522	*IKZF3*	C	T	T	48·29%	rs2941522	1·13 1·08–1·18)	1·46 × 10^−8^	1·10 (1·05–1·14)	1·97 × 10^−5^	1·11 (1·08–1·15)	2·32 × 10^−12^
17	47 439 302	rs112502960	*ZNF652*	G	A	A	35·92%	rs12952581 (0·98)	1·14 (1·09–1·20)	6·05 × 10^−9^	1·08 (1·04–1·13)	3·92 × 10^−4^	1·11 (1·08–1·15)	4·12 × 10^−11^

Results from case-control analyses for the variants that were significant in stage 1 and stage 2, showing the same direction of effect and reached genome-wide significance in the meta-analysis of stages 1 and 2. MAF corresponds to that from the stage 1 study and we give the OR per copy of the coded allele. rs1438673 was conditioned on rs1837253, rs1986009 was conditioned on rs3749833, and rs776111176 was conditioned on rs9273410. Stage 1 p values have genomic control applied.rs61816761 (FLG) was excluded and not included here following sensitivity analyses. r^2^ between stage 2 and stage 1 variants are given if a proxy was used in stage 2. MAF=minor allele frequency. rsid.ukb=rs number of variant used in stage 2 analyses using UK Biobank imputed data. OR=odds ratio.

To identify if the novel signals for moderate-to-severe asthma were also associated with all asthma including mild disease, we checked the association results from a large independent genome-wide association study.^5^ We found both the *KIAA1109* and *GATA3* signals were significantly associated with all asthma, including mild asthma (*KIAA1109* [proxy rs72687036 coded allele G]: OR 1·06, 95% CI 1·04–1·08; p=3·96 × 10^−7^; and *GATA3* [rs10905284, risk allele C]: OR 1·04, 1·02–1·06; p=2·72 × 10^−5^). No association was identified between the *MUC5AC* signal and all asthma (rs11603634, coded allele G: OR 1·00, 0·97–1·02; p=0·809; appendix).

In our systematic investigation of all previously described signals associated with asthma published to date (appendix),^5^, ^6^, ^7^, ^8^, ^9^, ^10^, ^11^, ^12^, ^13^, ^14^, ^15^, ^16^, ^17^, ^18^, ^19^, ^20^ we found that 60 (75%) of 80 previously reported SNPs showed an association with moderate-to-severe asthma (p<6 × 10^−4^ after Bonferroni correction), and a further ten (8%) SNPs showed a nominally significant association in our stage 1 cohort (p<0·05; appendix). The effect estimates (ORs) for previously described asthma signals in this study range from 1·08–1·24 (appendix). Similarly, we investigated previously reported SNPs associated with allergic diseases in two large studies^23^, ^34^ (appendix). Of the 136 signals identified in the study by Ferreira and colleagues,^24^ 87 (64%) were nominally associated (p<0·05) with moderate-to-severe asthma, 40 (29%) signals had an association (ie, p<6 × 10^−4^ after Bonferroni correction). Similarly, for the 38 signals identified in the study by Zhu and colleagues,^23^ 35 (92%) were nominally associated with moderate-to-severe asthma, with 28 (74%) signals meeting a Bonferroni corrected threshold.

**Figure 4:**
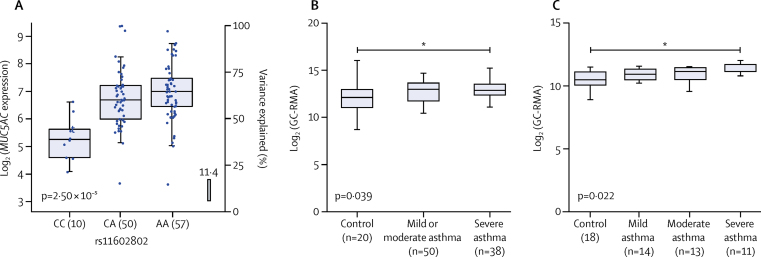
rs11603634 is an eQTL for *MUC5AC* in bronchial epithelial brush samples and *MUC5AC* mRNA expression is increased in bronchial epithelial cells from severe asthma patients (A) *MUC5AC* mRNA expression stratified by rs11602802 genotype. The boxes show the mean and SD and the whiskers show the IQR for each genotype. Generated from bronchial epithelial brush samples (n=117) collected as part of the U-BIOPRED study. rs11603634 was not directly genotyped, so the proxy rs11602802 was used. The rs11603634 asthma risk allele, G, is correlated with rs11602802, A, allele. (B) mRNA expression of *MUC5AC* in the GSE43696 dataset.^41^ Boxes showing the median and IQR, and the whiskers showing the minimum and maximum data, stratified by subject group. Bronchial epithelial brush samples were from controls (n=20), and patients with mild or moderate (n=50) and severe (n=38) asthma from the GSE43696 dataset and GC-RMA data for *MUC5AC. MUC5AC* levels were significantly higher in patients with severe asthma than in controls. (C) mRNA expression of *MUC5AC* in the GSE89809^42^ dataset. The boxes show the median and IQR, and the whiskers showing the minimum and maximum data, stratified by subject group. Bronchial epithelial brush samples were from controls (n=18), and patients with mild (n=14), moderate (n=13), and severe (n=11) asthma from the GSE89809 dataset and GC-RMA data for *MUC5AC* was extracted. *MUC5AC* RNA concentrations were significantly higher in patients with severe asthma than in controls. More details of datasets and analyses are in the appendix. eQTL=expression quantitative trait loci. GC-RMA=GeneChip robust multi-array average. U-BIOPRED=Unbiased BIOmarkers in PREDiction of respiratory disease outcomes. *p<0·05 by Kruskal-Wallace test.

**Figure 5:**
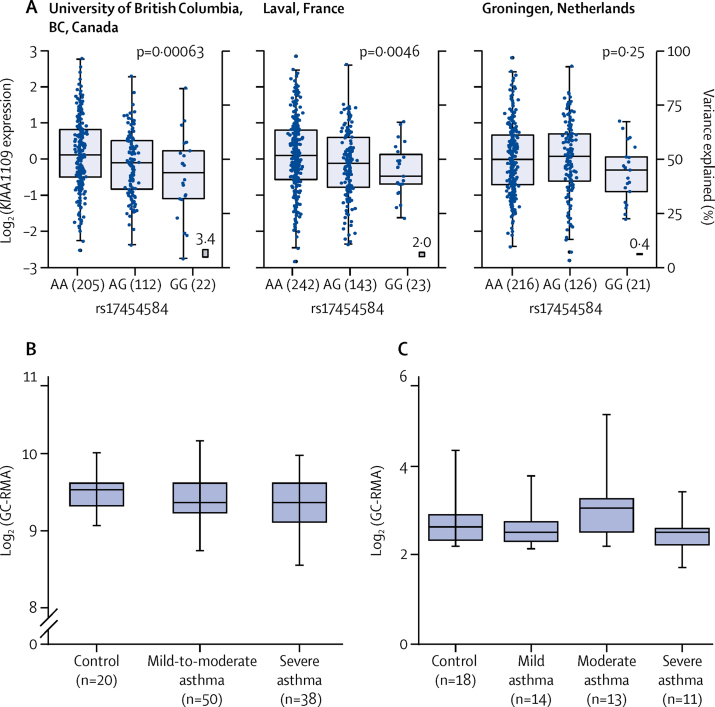
rs560026225 is an eQTL for *KIAA1109* in lung tissue and *KIAA1109* mRNA expression levels in bronchial epithelial cells from asthma patients (A) rs560026225 asthma risk allele, GATT, correlated with the rs17454584, G, allele. Data are for *KIAA1109* expression for each recruitment centre,^35^ stratified by rs17454584 genotype. The boxes show the mean and SD and whiskers show the IQR for each genotype. Non-tumour lung tissue was isolated from 1110 individuals who had undergone lung resection across three centres to generate the eQTL dataset. rs560026225 was not directly genotyped, so rs17454584 was used as a proxy. (B) *KIAA1109* expression levels in bronchial epithelial cells from asthma patients from the GSE43696 dataset.^41^ The boxes show the median and IQR, and whiskers the minimum and maximum data, stratified by subject group. Bronchial epithelial brush samples were from controls (n=20), and patients with mild or moderate (n=50) and severe (n=38) asthma in the GSE43696 dataset, and GC-RMA data for *KIAA1109*. No significant differences in *KIAA1109* expression levels between groups were observed (Kruskal-Wallace test). (C) *KIAA1109* expression levels in bronchial epithelial cells from asthma patients from the GSE89809 dataset.^42^ The boxes show the median and IQR, and whiskers the minimum and maximum data, stratified by subject group. Bronchial epithelial brush samples were from controls (n=18), and patients with mild (n=14), moderate (n=13), and severe (n=11) asthma from the GSE89809 dataset, and GC-RMA data for *KIA1109*. No significant differences in *KIAA1109* expression levels between groups were observed. See appendix for more details of datasets and analyses. eQTL=expression quantitative trait loci. GC-RMA=GeneChip robust multi-array average.

In our eQTL analyses of the three novel signals we found rs11603634 (*MUC5AC*) was an eQTL for *MUC5AC* in bronchial epithelial brush cells (via proxy SNP rs11602802, r^2^=0·46; appendix). The rs11603634 asthma risk allele (G) was correlated with rs11602802 (A) allele, which was associated with increased levels of *MUC5AC* mRNA (figure 4). Although they did not meet the 5% false discovery rate, *MUC5B* mRNA levels showed an opposite association with the rs11602802 SNP (appendix). Similarly, the proxy SNP rs17454584 (r^2^=0·57) for rs560026225 (*KIAA1109*) was an eQTL for *KIAA1109* in lung tissue with the asthma risk allele (GATT) associated with decreased expression of *KIAA1109* (rs560026225 GATT allele correlated with rs17454584 G allele; figure 5). No significant eQTL association was observed for the rs10905284 (*GATA3*) signal. We identified significant eQTL associations for 16 (76%) of 21 previously reported asthma signals in the lungs or blood, or both (appendix). We identified a large number of potential candidate causal genes in the type 2 inflammatory pathway, including the following subset: adaptive and innate immune response genes; *CD247, IL1RL1, IL18R1, TSLP*, human leukocyte antigen genes, *BACH2, IL33*, and *STAT6*, and genes that might be important in airway structural cell homoeostasis, integrity, or function—eg, *MUC5AC, D2HGDH, ING5, WDR36, RAD50, SLC22A5, SMAD3, ORMDL3, GSDMA*, and *GSDMB*.

We found *MUC5AC* and *KIAA1109* expression is present in airway epithelium and localised to the cytoplasm and membrane (appendix); therefore, we hypothesised that concentrations of *MUC5AC* and *KIAA1109* could be altered in airway epithelium in patients with severe asthma. We found high levels of *MUC5AC* mRNA in patients with severe asthma (figure 4) but did not identify any differential expression of *KIAA1109* in the same bronchial epithelial datasets (figure 5).

Using HaploReg, we identified a large number of potentially functional consequences of *MUC5AC, KIAA1109*, and *GATA3* sentinel SNPs and SNPs in linkage disequilibrium (appendix). In the *MUC5AC* locus, multiple SNPs including the lead SNP (rs11603634) altered Fox family transcription factors (appendix). For the *KIA1109* and *GATA3* signals, many potentially functional changes were apparent (appendix). Using DeepSEA, the moderate-to-severe asthma risk allele (G) at the sentinel SNP rs11603634 near *MUC5AC* was predicted to result in a log_2_ fold change of more than 1·5 in function at Forkhead Box A1 (FOXA1) and Forkhead Box A2 (FOXA2) binding sites in airway epithelium (appendix). For the rs10905284 (*GATA3*), the asthma risk allele (C; proxy rs3802597; r^2^=0·93) had an effect on upstream stimulatory factor 1 (USF1) and 2 (USF2) binding in various cell types, including airway epithelium (appendix). rs560026225 (*KIAA1109*; proxy rs17389644; r^2^=0·57) had a functional effect on a DNase hypersensitivity site in human microvascular endothelial and human umbilical vein endothelial cell types (appendix).

GRASP and GWAS catalog analyses of the three novel signals, and variants in linkage disequilibrium identified genome-wide significant associations. The rs560026225 (*KIAA1109*) asthma risk allele (GATT; allele associated with increased risk of moderate-to-severe asthma) was associated with risk of allergic sensitisation (proxy rs17454584, with the rs560026225 GATT allele correlated with rs17454584 G allele),^22^ the rs10905284 (*GATA3*) asthma risk allele (C) was associated with an increased number of eosinophils in the blood,^47^ and the rs11603634 (*MUC5AC*) asthma risk allele (G; proxy rs4077759, rs11603634 G allele correlated with rs4077759 T allele) was associated with risk of pulmonary fibrosis (appendix).^48^

## Discussion

To our knowledge, we present the largest genetic-association study of moderate-to-severe asthma to date, with 10 549 cases and 47 146 controls, identifying 24 signals that reach genome-wide significance in the meta-analysis of stages 1 and 2. 21 (88%) of 24 signals have previously been reported in studies that predominantly assessed samples from patients with mild asthma, suggesting a substantial shared genetic architecture between mild and moderate-to-severe asthma. Our findings suggest that additional factors might drive the development of more severe forms of asthma—eg, environmental exposures or epigenetics, and the presence of comorbidities. We also provide increased insight into the identification of candidate casual genes in many of these loci, including several genes associated with type 2 inflammation. Three signals we identify here have not previously been reported for asthma in genome-wide association studies: rs11603634 in the *MUC5AC* region (coded allele G, frequency 50·4%, risk), rs1090584 in *GATA3* (coded allele A, frequency 57·06%, protective), and rs560026225 (coded allele GATT, frequency 23·60%, risk) in a locus covering *KIAA1109.* The rs11603634 signal is specific to moderate-to-severe asthma and we identified that *MUC5AC* is increased in bronchial epithelial cells of carriers of the risk allele, with *MUC5B* being decreased in carriers of the risk allele, albeit not reaching our significance threshold. This genotype specific expression might be caused by alterations in FOXA transcription activity. For the *GATA3* and *KIAA1109* signals we saw an association with all asthma, while *GATA3* has been previously reported to be associated with increased concentrations of blood eosinophil^47^ and *KIAA1109* has been reported as being associated with self-reported allergy.^21^ Therefore, we provide additional insight into the genetic architecture of moderate-to-severe asthma and we report the first evidence that genetic variants associated with the risk of developing moderate-to-severe asthma regulate mucin production.

In this study, we identified 21 previously reported signals as being associated with asthma, including some previously associated with asthma that is severe or difficult to treat: *RAD50* and *HLA-DR/HLA-DQ*,^25^ and *17q21* (*ORMDL3/GSDMB/ZPB2*).^19^ Using eQTL analyses, we identified candidate causal genes for moderate-to-severe asthma; however, the level of evidence for each potential candidate gene based on linkage disequilibrium with sentinel SNP, relevant tissue and cell type, and statistical significance varied from highly supportive to suggestive. Overall, these signals highlight the role of innate and adaptive immunity and type 2 inflammation in moderate-to-severe asthma including: *CD247*, which encodes T-cell receptor ζ; trans-acting T-cell specific transcription factor GATA-3 (GATA3), an important transcription factor in T cells; interleukin-18 receptor 1 (IL18R1) and interleukin-1 receptor-like 1 (IL1RL1), receptors for key cytokines interleukin-18 and interlukin-33, respectively; thymic stromal lymphopoietin (TSLP), which drives type 2 inflammation; human leukocyte antigen genes that encode the major histocompatibility complex; transcription regulator protein BACH2 (BACH2), a transcriptional regulator in type 2 inflammation; interleukin-33 (IL33), an innate cytokine; and signal transducer and activator of transcription 6 (STAT6), a signalling molecule downstream of interleukin 4 and 13, which are drivers of type 2 inflammation. The other genes identified highlight roles in homoeostasis of airway cells, including D-2-hydroxyglutarate dehydrogenase (D2HGDH), which regulates α-ketoglutarate concentrations, influencing histone and DNA methylation;^49^ sodium/hydrogen exchanger 2 (SLC9A2), a sodium-hydrogen exchanger involved in the regulation of cell pH and volume;^50^ inhibitor of growth protein 5 (ING5), a transcription factor involved in epithelial to mesenchymal transition;^51^ DNA repair protein RAD50 (RAD50), which is involved in DNA double-strand break repair; solute carrier family 22 member 5 (SLC22A5), an organic cation transporter with a role in epithelial cells; protein CLEC16A (CLEC16A), a regulator of autophagy;^52^ gasdermin-B (GSDMB), which is linked with airway smooth-muscle contraction;^53^ and ORM1-like protein 3 (ORMDL3), which is linked with airway remodelling.^54^

This is the first report, to our knowledge, of the *MUC5AC* locus being specifically associated with increased susceptibility to development of moderate-to-severe asthma in a genome-wide association study. We showed this association in both stages 1 and 2 of our investigation, and in our meta-analyses; however, the higher severity of asthma in the cases (and associated power) in stage 1 than among those in stage 2 could explain the different significance levels between the stages—ie, cases in stage 1 had lower lung function than those in stage 2 and reported higher use of oral corticosteroids than those in stage 2. The proportion of cases with allergic comorbidities (allergic rhinitis or eczema, or both) or smoking history did not differ significantly between stages 1 and 2. The association between the asthma risk allele (G) of rs11603634 and increased concentrations of *MUC5AC* mRNA in bronchial epithelial brush samples, and the predicted effect on FOXA transcription factors provide putative mechanisms because FOXA2 regulates mucin-5AC (MUC5AC) production.^55^ We found this eQTL association to be the most significant for *MUC5AC* in the bronchial epithelial cell dataset. This signal of moderate-to-severe asthma has also previously been identified as associated with pulmonary fibrosis (proxy rs4077759, r^2^=0·42);^48^ however, rs11603634 reported in our study and rs35705950 (the main idiopathic pulmonary fibrosis signal) are not in linkage disequilibrium (r^2^=0·01), suggesting distinct signals. Similarly, the signal we reported is independent from that reported (rs1132440) in a candidate gene study for asthma.^56^ We also identified an association between rs11603634 on *MUC5B* mRNA levels in bronchial epithelial brush samples, with carriers of risk alleles having lower concentrations than those who are not carriers, although this association did not meet our significance threshold. MUC5AC protein concentrations are increased in the sputum of patients with asthma during exacerbations compared with patients with stable asthma and controls, whereas concentrations of mucin-5B (MUC5B) are decreased among patients with asthma—ie, alterations in the ratio of MUC5AC to MUC5B are a feature of asthma.^57^ This altered ratio of MUC5AC to MUC5B in asthma might be partially explained by the opposite effect of our novel asthma risk allele (G) of rs11603634 on MUC5AC and MUC5B production. MUC5AC has pathogenic roles and has been linked to airway hyper-responsiveness and mucus plugging during exacerbation.^58^ *MUC5AC* deficient mice develop allergic airway disease; however, the severity and abundance of mucus plugging is attenuated.^59^ Loss of MUC5B in a mouse knock-out study led to airway inflammation, suggesting a role in homoeostasis.^60^ Overall, these data suggest targeting of specific mucins could be a therapeutic opportunity for moderate-to-severe asthma.

The *KIAA1109* (rs72687036) novel signal we identified has previously been associated with self-reported allergy^21^ and allergic sensitisation,^22^ type 1 diabetes,^61^ ulcerative colitis,^62^ mean platelet volume,^47^ and with allergic disease (asthma, hayfever, allergic rhinitis, or eczema).^23^ The region is rich in candidate genes (eg, *IL2* and *IL21*); however, our eQTL data suggest that the potential causal gene is *KIAA1109*. Little is known about *KIAA1109*. Mice deficient in *KIAA1109* have preweaning lethality (International Mouse Phenotyping Consortium) and a suggested role in synaptic vesicle recycling in Drosophila has been identified.^63^

The third novel signal we identified, rs1090584 in *GATA3*, asthma risk allele C, is also associated with rheumatoid arthritis (proxy rs3824660; r^2^=0·86)^64^ and increased concentrations of blood eosinophils,^47^ a known effector cell in asthma. This signal has been associated with allergic disease (asthma, hayfever, allergic rhinitis, or eczema).^23^ The rs1090584 signal in *GATA3* identified in this study is independent to those previously described for asthma, including rs10508372 in Japanese individuals,^15^ rs2589561 in European or multi-ancestry individuals,^11^ and rs12413578 in European individuals.^5^ The second signal in *GATA3* (rs61840192) we report is in linkage disequilibrium with rs12413578 and rs2589561 (r^2^=0·16). These data suggest that multiple genetic signals within the *GATA3* locus might contribute to asthma. We identified potential effects of rs1090584 on USF1 and USF2 in airway cells; however, we did not identify an eQTL association. USF1 is important in regulating GATA family genes, including *GATA5*.^65^ GATA3 is a transcriptional regulator associated with differentiation—eg, in type-2 innate lymphoid cell differentiation,^66^ an effector cell in type 2 inflammation.

We also investigated all previously reported asthma signals to date, with general replication of previous results. The previously described signals that did not replicate in our dataset were associated with a specific asthma phenotype (eg, *PDE4D* and mild-to-moderate childhood asthma with bronchial hyper-responsiveness^16^) or were reported in people of non-European ancestry (eg, *NOTCH4*^15^). The effect sizes for previously described asthma signals in stage 1 of this moderate-to-severe asthma cohort (OR range 1·08–1·24) are comparable with those reported in large studies of asthma.^5^, ^11^ Similarly, we investigated allergic disease signals identified in two large genome-wide association studies,^23^, ^24^ and found a large proportion of these signals were associated with moderate-to-severe asthma; however, our case-control design used moderate-to-severe asthma cases and controls excluding individuals with asthma, rhinitis, eczema, and allergy diagnosed by a doctor, and so the ability to identify genetic signals associated with allergic comorbidities in the asthma population will be enhanced compared with these previous studies.

Our study had several limitations. Regarding the design of the study, we considered alternative approaches when planning the analysis, such as using patients with mild asthma as the control group. However, we opted to compare patients with moderate-to-severe asthma with healthy controls in the initial discovery analysis because we felt that this comparison would minimise the risk of misclassification between mild and moderate asthma and so be a more powerful strategy for a genetic study. We included a large genome-wide association study of asthma^5^ to specifically address the issue of specificity of the signals we found in our cohort to moderate-to-severe asthma. Cases in stage 1 have more severe asthma than those in stage 2 because patients from GASP and U-BIOPRED in stage 1 were predominantly recruited from secondary care. This potential difference between cases in stage 1 and 2 might have contributed to the attenuated association with, for example, the *MUC5AC* signal in the stage 2 cohort; however, this difference also potentially provided additional power for stage 1. We also acknowledge that we defined asthma severity on the basis of medication use alone and additional measures including symptoms, exacerbation frequency, and other markers would have enhanced the definition, although these data were not available. Importantly, all cases in our analyses required a doctor diagnosis of asthma for inclusion before stratification on the basis of medication. Severe asthma is defined by the requirement for high dose of inhaled corticosteroid or maintenance oral corticosteroid, with persistent poor control or a high risk of developing poor control if these therapies are stepped down. Similarly, the Global Initiative for Asthma treatment steps are used as surrogates of severity. Thus, using the treatment step as a measure of severity is in keeping with current guidelines and has the advantage that it provides maximum sample sizes because medication data are available for a greater number of patients than, for instance, symptom scores. Additionally, for cases who were prescribed oral corticosteroids, we did not undertake individual case reviews to exclude the possibility that some patients might have been receiving oral corticosteroids for other comorbid conditions, and we did not record additional information on current asthma control or exacerbation frequency, which would have been informative. Advances in imputation mean that the widely used threshold for genome-wide significance of a p value of less than 5 × 10^−8^, historically defined on the basis of 1 million independent tests, could be considered too lenient for an analysis of 33 million SNPs (representing >1 million independent tests). Had we applied a threshold p value of less than 5 × 10^−9^, recommended as an appropriate threshold for studies of whole-genome sequence data from European populations,^67^ the signals at *GATA3* (p=1·76 × 10^−10^) and *KIAA1109* (p=3·06 × 10^−9^) would still have been significant. Overall, the *MUC5AC* signal was weaker in terms of significance compared with other signals identified—eg, in stage 2, this signal only met nominal significance not Bonferroni correction and was just within the threshold for genome-wide significance in the meta-analysis. Finally, asthma is a complex disease involving both genetic and environmental influences and we have not formally assessed the role of the environment, which could be critical for the development of more severe asthma. Accumulating data suggest a role for several factors in the development of severe disease (eg, comorbidities such as atopic dermatitis in severe asthma^68^ and environmental or epigenetic mechanisms^69^) but further work is needed.

In summary, to our knowledge, this is the largest genome-wide association study of moderate-to-severe asthma published to date, in which we have identified that the genetic architecture of moderate-to-severe asthma is similar to mild disease, three novel genome-wide significant associations, and potential candidate causal genes underlying these signals. These findings provide new understanding for this difficult to treat population and adds to the accumulating evidence that strategies to target mucins might have therapeutic value. Similarly, our findings add to evidence that targeting type 2 inflammation in asthma could be particularly useful for moderate-to-severe asthma, potentially in carriers of genetic variants in genes of relevance to innate or adaptive immunity.
