# Supplementary material for: Moderate-to-severe asthma in individuals of European ancestry: a genome-wide association study
Source: Lancet Respir Med. 2019 Jan;7(1):20–34. doi: 10.1016/S2213-2600(18)30389-8 (PMC6314966; doi:10.1016/S2213-2600(18)30389-8)
Supplement: Supplementary appendix [file mmc1.pdf]

THE LANCET
Respiratory Medicine

Supplementary appendix

This appendix formed part of the original submission and has been peer reviewed. We post it as supplied by the authors.

Supplement to: Shrine N, Portelli MA, John C, et al. Moderate-to-severe asthma in individuals of European ancestry: a genome-wide association study. *Lancet Respir Med* 2018; published online Dec 11. http://dx.doi.org/10.1016/S2213-2600(18)30389-8.

Supplementary Material

Stage 1 case and control selection

Genotyping quality control

In each genotyping batch samples were excluded: (i) if their genetically inferred sex didn't match their sex in the sample manifest; (ii) if they had outlying heterozygosity within the batch (outside either 2 or 3 standard deviations from the mean depending on batch); (iii) if they had a call rate < 95% across genotyped variants; (iv) if cryptically related to another sample (2nd degree related or higher using IBD estimates calculated by PLINK 1.90), 1 sample of the pair was removed; (v) if the sample shows significant deviation from European ancestry as determined by a plot of the first two principal components calculated using EIGENSOFT 6.1.4.

Genetics of Asthma Phenotypes & Severity (GASP) cases for stage 1

Moderate-severe asthma cases were recruited as part of GASP defined as moderate-severe asthma as per the British Thoracic Society (BTS) guidelines (2014) and the British National Formulary (September 2015) essentially as previously described[1]. We recruited adult subjects with asthma (age 16-60) from eight UK centres; Nottingham (recruited 1990-2015), Belfast (recruited 2008-2009), Birmingham (2005-2014), Cumbria (recruited 2013-2015), Manchester (recruited 2008-2014), Southampton (recruited 2003-2014), Glasgow (recruited 2002-2014) and Leicester (recruited 2004-2015). 2,976 GASP samples were genotyped, 744 using the Affymetrix Axiom® UK BiLEVE array and 2,232 with the Affymetrix Axiom® UK Biobank array. 688 samples failed genotyping quality control, leaving 2,288 samples in the genotype imputation. Out of the 2,288 samples in the genotype imputation 1 was excluded for relatedness and 429 did not meet the criteria for moderate-severe asthma leaving 1,858 cases in the analysis.

Unbiased BIOmarkers in PREDiction of respiratory disease outcomes (U-BIOPRED) cases and controls for stage 1

Recruitment to the U-BIOPRED cohort involved 16 clinical centres in 11 European countries and has been described in detail[2] (recruited 2013-2015). 755 U-BIOPRED samples were genotyped on the Affymetrix Axiom® UK Biobank array. Following quality control, 281 U-BIOPRED cases (as per criteria described for GASP) and 75 controls were included in stage 1.

Selection of UK Biobank cases for stage 1

Genotyping data was available from UK Biobank for 152,729 of the 502,682 individuals recruited by UK Biobank (May 2015 interim release). 480 individuals were removed as recommended by UK Biobank for outlying genome-wide heterozygosity or having more than 5% missing genetic data. 6,656 individuals were excluded as non-European ancestry (data field 21000 – values other than 1, 1001, 1002, 1003).

Moderate-severe asthma subjects were selected from UK Biobank under the following criteria; Asthma – doctor diagnosis (data field 6152), Exclude doctor diagnosed Emphysema/chronic bronchitis (data field 6152) and moderate-severe asthma based on British Thoracic Society (BTS) guidelines (2014) and the British National Formulary (September 2015) (data field 20003). From 146,073 European ancestry individuals available in the first release of UK Biobank genetic data, 18,010 subjects recorded yes to doctor diagnosed asthma and 3,105 met the criteria for moderate-severe asthma, 2,996 of which passed genotyping quality control.

Defining moderate-severe asthma

For the GASP and U-BIOPRED cohorts recruited from secondary care in specialist respiratory centres moderate-severe asthma definition was as per the BTS guidelines (2014) and the British National Formulary (September 2015) and was defined by the collaborating centre, i.e. BTS stage 3-5 criteria; i.e. for Stage 3 taking - long acting β_2 agonist, plus inhaled corticosteroid (ICS), Stage 4 – higher dose ICS and addition of a fourth drug e.g. leukotriene receptor antagonist, SR theophylline, and Stage 5 – oral corticosteroid (OCS) and/or Omalizumab.

In UK Biobank cases were defined by (i) having doctor diagnosed asthma, (ii) taking medication for asthma, (iii) exclusion of doctor diagnosed emphysema/chronic bronchitis and (iv) meeting the definition of moderate-severe asthma using British Thoracic Society (BTS) criteria. Cases were therefore selected from individuals for whom medication information was available and who met BTS stage 3-5 criteria as outlined.

Medicines relevant to all asthma subjects and those used to identify moderate-severe asthma in UK Biobank are listed below.

Medicines relevant to all asthma diagnosed subjects including UKB id code

1140855298	salbulin 2mg tablet
1140855302	salbulin 2mg/2ml liquid
1140855304	salbuvent 2mg tablet
1140855308	salbuvent 2mg/5ml syrup
1140855372	asmaven 100micrograms inhaler
1140855374	cobutolin 100micrograms inhaler
1140855376	aerolin-400 100micrograms inhaler
1140855380	salbuvent 100micrograms inhaler
1140855384	salbuvent 5mg/ml respirator solution
1140855390	monovent 5mg tablet
1140855466	bextasol 100micrograms inhaler
1140855500	asma-vydrin spray
1140855508	bronchilator inhaler
1140855520	duo-autohaler inhaler
1140855524	iso-brovon pressurised inhaler
1140855528	medihaler duo inhaler
1140855534	rybar no-1 inhaler
1140855536	rybar no-2 inhaler
1140855538	taumasthman tablet
1140861996	salbulin 100micrograms inhaler
1140861998	ventolin 100micrograms inhaler
1140862008	steri-neb salamol 2.5mg/2.5ml nebulisation unit
1140862016	ventodisks 200micrograms/blister disks+diskhaler
1140862060	ventolin 2mg tablet
1140862066	volmax 4mg m/r tablet
1140862070	ventolin cr 4mg m/r tablet
1140862086	salamol 100micrograms inhaler
1140862110	berotec 100 inhaler
1140862118	pirbuterol
1140862120	exirel 10mg capsule
1140862124	exirel 200micrograms inhaler
1140862134	bronchodil 500micrograms inhaler
1140862140	pulmadil 200micrograms inhaler
1140862380	becloforte 250micrograms inhaler
1140862382	becotide 50 inhaler
1140862406	filair 50 inhaler
1140862476	beclazone 50 inhaler
1140862526	sodium cromoglycate
1140862532	intal 5mg inhaler
1140862560	ventide inhaler
1140862572	budesonide
1140862574	pulmicort ls 50micrograms inhaler
1140862584	pulmicort ls 50micrograms spacer inhaler
1140862600	cromogen 5mg inhaler
1140862610	aerocrom inhaler
1140881856	salbutamol
1141157418	budesonide product
1141157486	salbutamol product
1141195280	novolizer budesonide 200micrograms/dose cartridge+inhaler

Medicines identifying moderate-severe asthma including UKB id code

1140883548	ipratropium
1141182628	tiotropium
1140855320	isoetharine hydrochloride*
1140855322	numotac 10mg m/r tablet
1140855328	aleudrin 20mg tablet**
1140855330	aleudrin 1% spray for nebuliser**
1140855332	iso-autohaler 80micrograms inhaler**
1140855358	theodrox tablet
1140855360	sabidal sr-270 424mg m/r tablet
1140855366	pro-vent 300mg m/r capsule
1140855400	bronchodil 20mg tablet
1140855424	theograd 350mg m/r tablet
1140855426	biophylline 350mg m/r tablet
1140855442	adrenaline+atropine compound spray
1140855496	alupent expectorant 20mg tablet*
1140855504	bricanyl compound tablet
1140855506	bricanyl expectorant elixir
1140855530	nethaprin dospan m/r tablet
1140855540	tedral tablet
1140855542	tedral elixir
1140855540	tedral tablet
1140855542	tedral elixir
1140862092	maxivent 100micrograms inhaler
1140862144	salmeterol
1140862148	serevent 25mcg inhaler
1140862162	bricanyl 5mg tablet
1140862168	bricanyl 250mcg inhaler
1140862222	brelomax 2mg tablet
1140862224	respacal 2mg tablet
1140862236	atrovent 20micrograms inhaler
1140862260	aminophylline
1140862266	phyllocontin continus 225mg m/r tablet
1140862274	pecram 225mg m/r tablet
1140862280	bambec 10mg tablet
1140862290	adrenaline product
1140862292	medihaler-epi 280micrograms inhaler
1140862294	adrenaline acid tartrate 280micrograms inhaler
1140862306	cam 4mg/5ml s/f mixture
1140862310	medihaler-iso 80micrograms inhaler
1140862320	alupent 20mg tablet*
1140862336	amnivent 225 sr m/r tablet
1140862346	choline theophyllinate
1140862348	choledyl 100mg tablet
1140862362	duovent inhaler
1140862364	franol tablet
1140862374	ephedrine hydrochloride+theophylline 11mg/120mg tablet
1140862412	theophylline product
1140862414	nuelin 125mg tablet
1140862418	lasma 300mg m/r tablet
1140862424	slo-phyllin 60mg m/r capsule
1140862432	theo-dur 200mg m/r tablet
1140862438	uniphyllin continus 200mg m/r tablet
1140862474	aerobec 50mcg autohaler
1140868364	prednisone
1140868370	decortisyl 5mg tablet

1140881862	oxivent 100mcg inhaler
1141157264	salmeterol product
1141157402	prednisolone product
1141173346	cortisone
1141176832	seretide 50 evohaler
1141182632	spiriva 18micrograms inhalation capsule
1141195224	formoterol
1141195232	budesonide+formoterol
1141157126	montelukast product
1141157132	singulair 10mg tablet
1141168340	zafirlukast
1141168344	accolate 20mg tablet

* Historic first-line bronchodilator. Now only used in severe asthma for when the patient is on standard therapy ** Historic bronchodilator

Selection of UK Biobank controls for stage 1

Controls were selected from UK Biobank under the following criteria; disease exclusion 1 (doctor diagnosed asthma, rhinitis, eczema, allergy, Emphysema/chronic bronchitis) (data field 6152), disease exclusion 2: individuals taking medicine for lung related conditions (data field 20003), exclude if one or more admission for asthma (ICD10 codes J45-J46) or COPD & bronchiectasis (ICD10 codes J40-J44, J47). From 146,073 European ancestry individuals available in the first release of UK Biobank genetic data, this selection gave 97,267 individuals available prior to age and sex matching to cases. 80,629 selected controls passed genotyping quality control and from these we selected 25,600 samples to give a 1:5 case-control ratio to balance statistical power and computational time.

Stage 2 case and control selection

Selection of moderate-severe asthma patients from UK Biobank for stage 2 and 2a

The second release of UK Biobank included 488,377 individuals of which 460,223 were of self-reported white ethnicity (data field 21000 - codes 1, 1001, 1002, 1003). From those not already included in stage 1, we identified 5,756 potential cases using the same criteria as stage 1 of which 5,499 passed genotyping quality control. 85 related individuals (2nd degree or closer) were excluded (68 related to stage 1 samples; 17 related to other stage 2 samples) leaving 5,414 severe asthma cases for replication.

Selection of UK Biobank controls for stage 2 and 2a

In the full release of UK Biobank genotype data 314,431 individuals of self-reported white ethnicity who were not genotyped in the interim release from which stage 1 sample were selected. From these we identified 275,820 eligible controls by excluding individuals with self-reported doctor-diagnosed asthma or emphysema/chronic bronchitis (data field 6152). 275,144 samples passed genotyping quality control from which we excluded 28,504 samples that were 2nd degree or closer related to stage 1 or stage 2. From the remaining 246,640 we selected 27,082 age and gender matched controls to give a 1:5 case-control ratio, and from these we additionally excluded 5,611 individuals with self-reported rhinitis, eczema and allergy in data field 6152 leaving 21,471 controls in the stage 2 analysis. For stage 2a (sensitivity analyses) we used all 27,082 age and gender matched controls prior to exclusion of self-reported rhinitis, eczema and allergy in data field 6152.

Phasing and imputation

To improve accuracy, phasing and imputation was done across all samples in the Stage 1 genotyping batches which included the case and control samples for this study. Accordingly, after quality control there were 102,247 samples included in the phasing and imputation. Variants with minor allele frequency <1%, Hardy-Weinberg $P < 10^{-6}$ and call rate <95% were excluded leaving 589,812 quality controlled variants to be phased and used as the imputation input.

Phasing was performed using SHAPEIT v3[3] and imputation was performed using IMPUTE v2.3.2[4] using combined 1000 genomes phase 3 and UK10K[5] reference panels (merged with IMPUTE v2.3.2) comprising 81.6 million variants. After imputation we retained 33.6 million well-imputed variants (imputation INFO score > 0.5 and minor allele count >= 3) for association testing.

In stage 2 we used the genotypes provided with the UK Biobank final release comprising 39 million variants imputed to the Haplotype Reference Consortium (HRC) [Bycroft 2017 https://doi.org/10.1101/166298].

Stage 1 genome-wide analyses

Genome-wide association testing was run with SNPTEST v2.5[6] using a logistic model to associate moderate-severe asthma risk with imputed genotype dose (continuous from 0-2 reflecting imputation uncertainty), including the first 10 ancestry principal components as the only covariates (samples were matched in age-sex strata during selection) and using the fast score test option for computational speed.

We applied genomic control using the LD score regression intercept (LDSC software)[7] as the inflation factor λ to appropriately correct test statistics for inflation due to confounding (Supplementary Figure 1).

Selection of signals for stage 2

We used a threshold of $P < 10^{-6}$ to select signals for follow up in stage 2. In order to define our initial set of associated loci, we selected the most significant variant as the sentinel variant and all variants within ±500kb as belonging to the locus represented by this sentinel. We then select the next most significant variant outside the newly defined locus as the next sentinel and so on until there are no more variants with $P < 10^{-6}$ not already attributed to a locus. We then performed stepwise conditional analysis on the variants within each locus, conditioning on the sentinel variant initially, to identify independent signals within each 1 Mb locus, thereby obtaining a set of independently associated signals for follow up in stage 2.

Checking for array effects in stage 1

In stage 1 1707 cases and 8329 controls (1:4.88 case-control ratio) were genotyped on the Affymetrix Axiom® UK BiLEVE array[8] and 3428 cases and 17346 controls on the Affymetrix Axiom® UK Biobank array (1:5.06 case-control ratio) with moderate-severe asthma case status independent of array (Fisher exact test P=0.27). We performed a sensitivity analysis of the stage 1 association testing to any array effects by repeating the association test with array as a covariate for our 32 signals followed up in stage 1. There was no significant change in effect size (Supplementary Figure 2) or association P value between adjusted and unadjusted results.

Stage 2 case-control analyses and meta-analyses

The stage 2 and 2a (sensitivity) replication association testing was performed in an identical manner to stage 1. Stage 1 and stage 2 were meta-analysed using inverse-variance weighted meta-analysis. In cases where a variant was not available or poorly imputed in stage 2, a proxy was chosen (rsid.ukb in Supplementary Table 2) and used in the meta-analysis of stage 1 and stage 2.

Expression quantitative trait loci (eQTL)

The variants identified as associated with susceptibility to moderate-severe asthma as well as variants in LD ($r^2 > 0.4$) were investigated in two eQTL datasets; a blood eQTL database[9] and a lung eQTL database[10,11]. The blood cell resource contained eQTL data for variants showing any association with peripheral blood samples from 5,311 individuals. Subjects were genotyped on various platforms and imputed using HapMap. The lung eQTL database consisted of non-tumour lung tissue from 1,110 individuals who had undergone lung resection and genotyped on the Illumina Human1M-Duo BeadChip array. A FDR threshold of 10% was used for eQTLs in these two databases.

To complement these eQTL resources we also had access to an additional five eQTL datasets generated as part of the U-BIOPRED initiative including; blood n=345, sputum n=91, biopsy n=84, bronchial brushing n=117 and nasal brushing n=75. To maximise power we included all subjects from U-BIOPRED in the eQTL analyses, i.e. a: severe non-smoking asthma, b: severe smoking asthma, c: mild-moderate asthma and d: healthy controls.

Traits	Blood (n)	Sputum (n)	Biopsy (n)	Bronchial brushing (n)	Nasal brushing (n)
Cohort(a/b/c/d)	161/65/59/60	44/19/16/12	36/10/22/16	41/14/29/33	26/9/19/21

The U-BIOPRED cohort was genotyped using the Affymetrix Axiom® UK Biobank array and after imputation (SHAPEIT2 for pre-phasing) and IMPUTE 2, using 1000 Genome-phase3 as reference panel) and quality control, there were 2,536,796 SNPs for association testing. Transcriptomic analysis was performed with the Affymetrix HT HG-U133 1 PM GeneChip (Affymetrix, Santa Clara, Calif) on extracted RNA. Technical and biological quality checks were performed according to Affymetrix recommendations with only RNA samples of high purity (RNA integrity number > 6.5) used for amplification [12,13]. A FDR threshold of 5% was used for

the smaller U-BIOPRED eQTL resources. For the MUC5AC rs11603634 eQTL (via proxy rs11602802) identified in bronchial brushings we also repeated analyses in asthma subjects alone with similar findings.

mRNA analyses in control and asthma subject biological samples

To determine the relative expression of *MUC5AC* and *KIAA1109* in bronchial epithelial cell brush samples from asthma and control subjects we used two freely available gene expression datasets from the Gene Expression Omnibus (GEO) resource (https://www.ncbi.nlm.nih.gov/geo/). The datasets included; GSE43696 which includes Agilent Human GE 4×44K V2 Gene Expression data for 20 control, 50 mild and 38 severe asthma subjects[14]. Asthma severity was defined based on; mild/moderate-severe asthma had an FEV_1 of >60%predicted, with/without low–moderate dose inhaled CS and severe asthma subjects had continuous use of high-dose inhaled CS and/or frequent use of oral CS with continuing symptoms and/or chronic airflow limitation. We also used,GSE89809 which includes Affymetrix HT HG-U133+ PM GeneChip data for 18 control, 14 mild, 13 moderate and 11 severe asthma subjects[15]. Asthma severity was defined based on; mild patients with asthma on β_2-agonists alone, 17 subjects with moderate asthma on ICS and 14 subjects with severe asthma with persistent symptoms despite high-dose ICS (n =14) and oral corticosteroids (n = 4).

References

1. Wan YI, Shrine NR, Soler Artigas M, et al. Genome-wide association study to identify genetic determinants of severe asthma. *Thorax* 2012; **67**(9): 762-8.
2. Shaw DE, Sousa AR, Fowler SJ, et al. Clinical and inflammatory characteristics of the European U-BIOPRED adult severe asthma cohort. *Eur Respir J* 2015; **46**(5): 1308-21.
3. O'Connell J, Sharp K, Shrine N, et al. Haplotype estimation for biobank-scale data sets. *Nature Genetics* 2016; **48**: 817.
4. Howie BN, Donnelly P, Marchini J. A Flexible and Accurate Genotype Imputation Method for the Next Generation of Genome-Wide Association Studies. *PLOS Genetics* 2009; **5**(6): e1000529.
5. Huang J, Howie B, McCarthy S, et al. Improved imputation of low-frequency and rare variants using the UK10K haplotype reference panel. *Nature Communications* 2015; **6**: 8111.
6. Marchini J, Howie B, Myers S, McVean G, Donnelly P. A new multipoint method for genome-wide association studies by imputation of genotypes. *Nature Genetics* 2007; **39**: 906.
7. Bulik-Sullivan BK, Loh P-R, Finucane HK, et al. LD Score regression distinguishes confounding from polygenicity in genome-wide association studies. *Nature Genetics* 2015; **47**: 291.
8. Wain LV, Shrine N, Miller S, et al. Novel insights into the genetics of smoking behaviour, lung function, and chronic obstructive pulmonary disease (UK BiLEVE): a genetic association study in UK Biobank. *Lancet Respir Med* 2015; **3**(10): 769-81.
9. Westra HJ, Peters MJ, Esko T, et al. Systematic identification of trans eQTLs as putative drivers of known disease associations. *Nat Genet* 2013; **45**(10): 1238-43.
10. Hao K, Bosse Y, Nickle DC, et al. Lung eQTLs to help reveal the molecular underpinnings of asthma. *PLoS Genet* 2012; **8**(11): e1003029.
11. Lamontagne M, Couture C, Postma DS, et al. Refining susceptibility loci of chronic obstructive pulmonary disease with lung eqtls. *PLoS One* 2013; **8**(7): e70220.
12. Hekking PP, Loza MJ, Pavlidis S, et al. Pathway discovery using transcriptomic profiles in adult-onset severe asthma. *J Allergy Clin Immunol* 2017.
13. Lefaudeux D, De Meulder B, Loza MJ, et al. U-BIOPRED clinical adult asthma clusters linked to a subset of sputum omics. *J Allergy Clin Immunol* 2017; **139**(6): 1797-807.
14. Voraphani N, Gladwin MT, Contreras AU, et al. An airway epithelial iNOS-DUOX2-thyroid peroxidase metabolome drives Th1/Th2 nitrative stress in human severe asthma. *Mucosal Immunol* 2014; **7**(5): 1175-85.
15. Singhania A, Wallington JC, Smith CG, et al. Multi-tissue Transcriptomics Delineates the Diversity of Airway T Cell Functions in Asthma. *Am J Respir Cell Mol Biol* 2017.

Supplementary Figures and Tables

Supplementary Figure 1: Quantile-quantile (QQ) plot for Stage 1: the unadjusted genomic inflation factor λ was 1.124. After correcting for polygenicity using LD score regression[7] the residual inflation factor used for genomic control was 1.029.

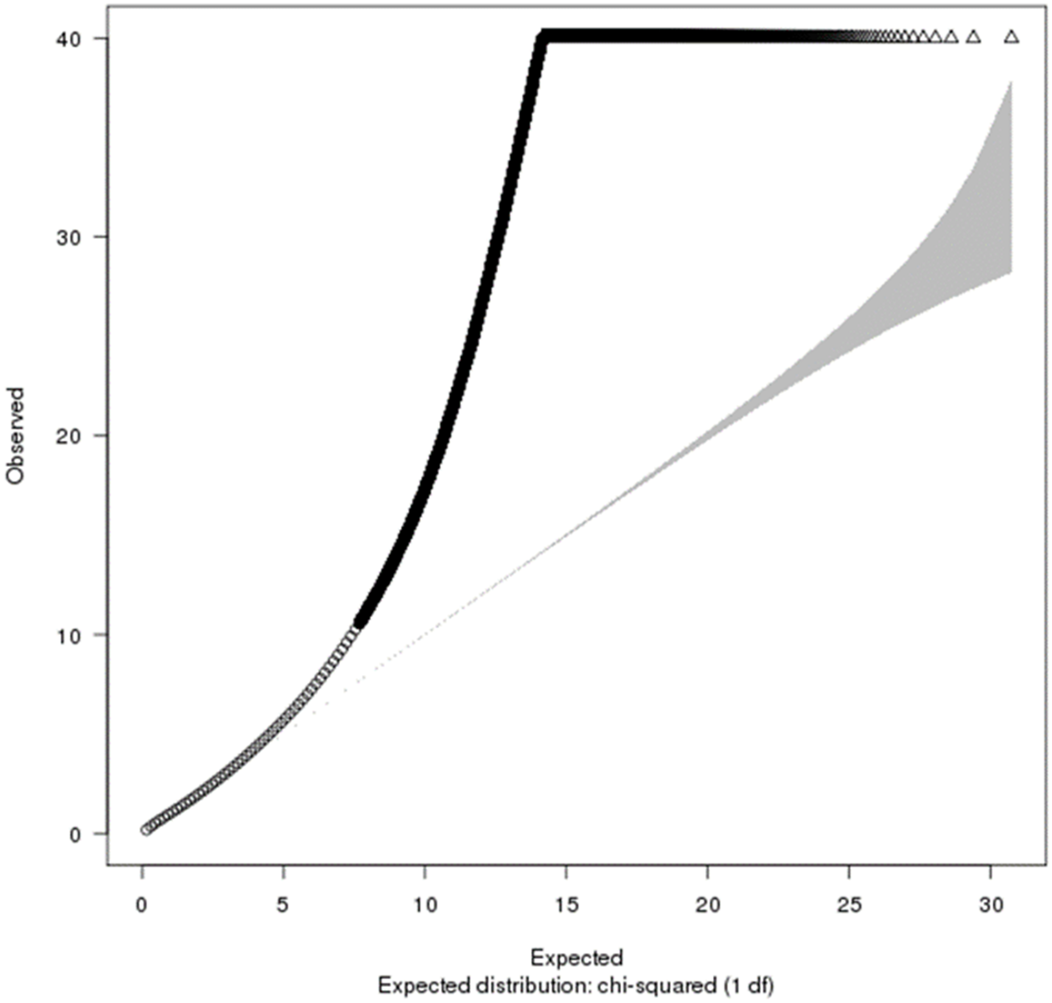

Supplementary Figure 2: Sensitivity of association testing to array effects. The effect sizes for the 32 signals followed up from stage 1 are plotted, betas (log odds ratios) adjusted for array effects vs. unadjusted betas. Error bars are the standard errors of the effect estimates.

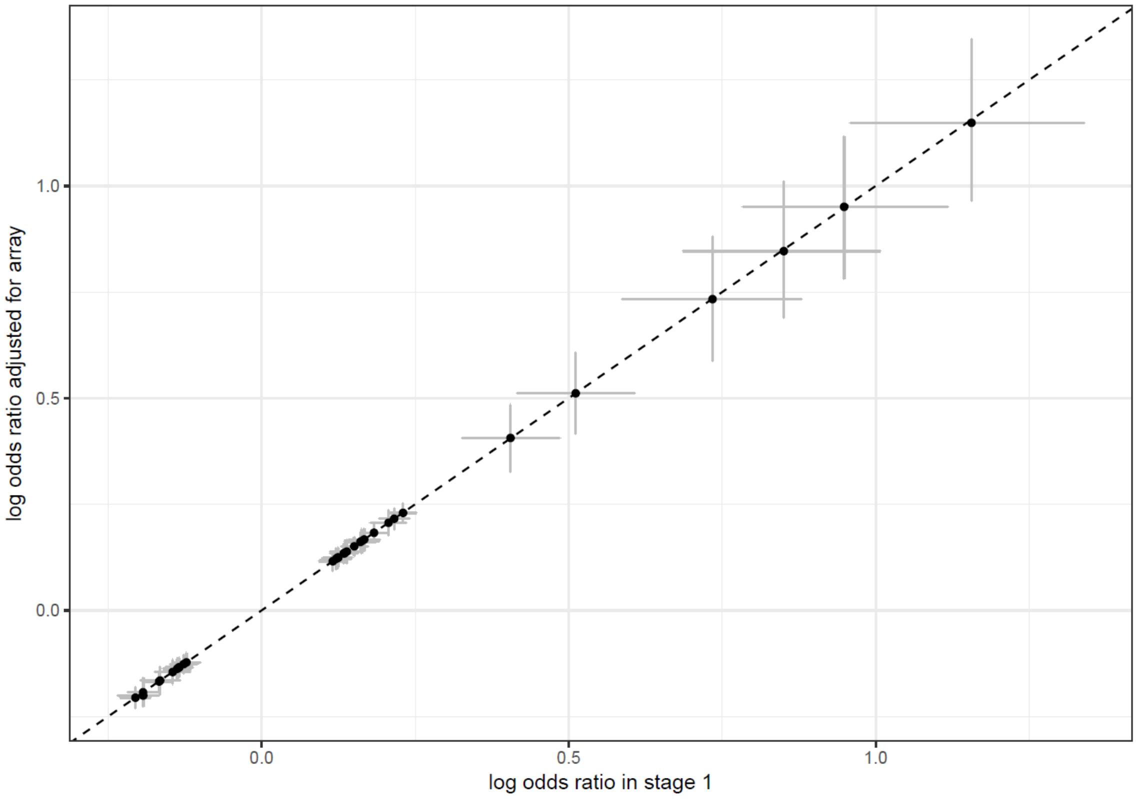

Supplementary Figure 3: Regional association plots for 32 signals taken forward in stage 1. Significance of each single nucleotide polymorphism (SNP) on the –log10 scale as a function of chromosome position (NCBI build 37). The sentinel SNP at each locus is shown by the blue peak, and data points are colour coded to show the correlations (r^2) of each of the surrounding SNPs to the sentinel SNP. The green line indicates criteria for inclusion in stage 2 ($P < 1\times10^{-6}$) and the red line indicates genome-wide significance ($P < 5\times10^{-8}$).

rs301819 *RERE*

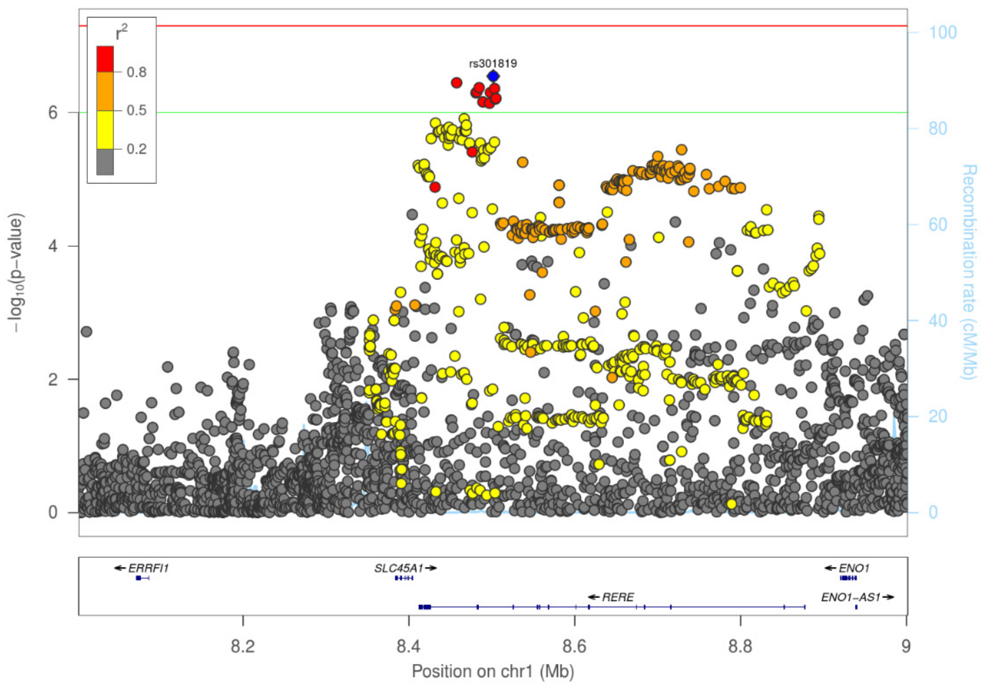

rs137907825 *TNFRSF8*

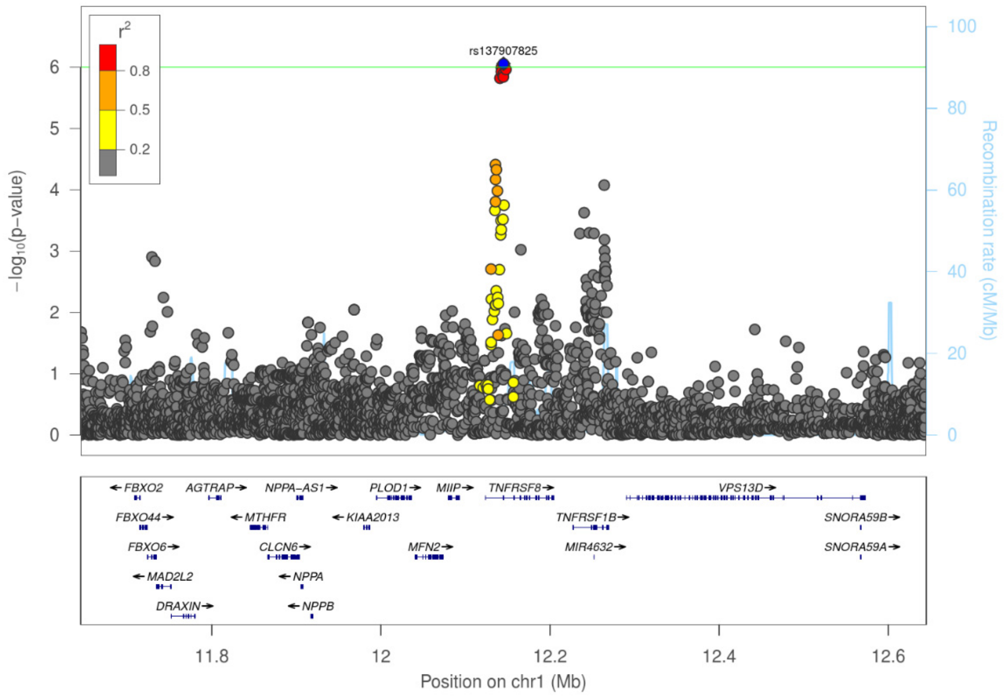

rs147639436 *PIFO*

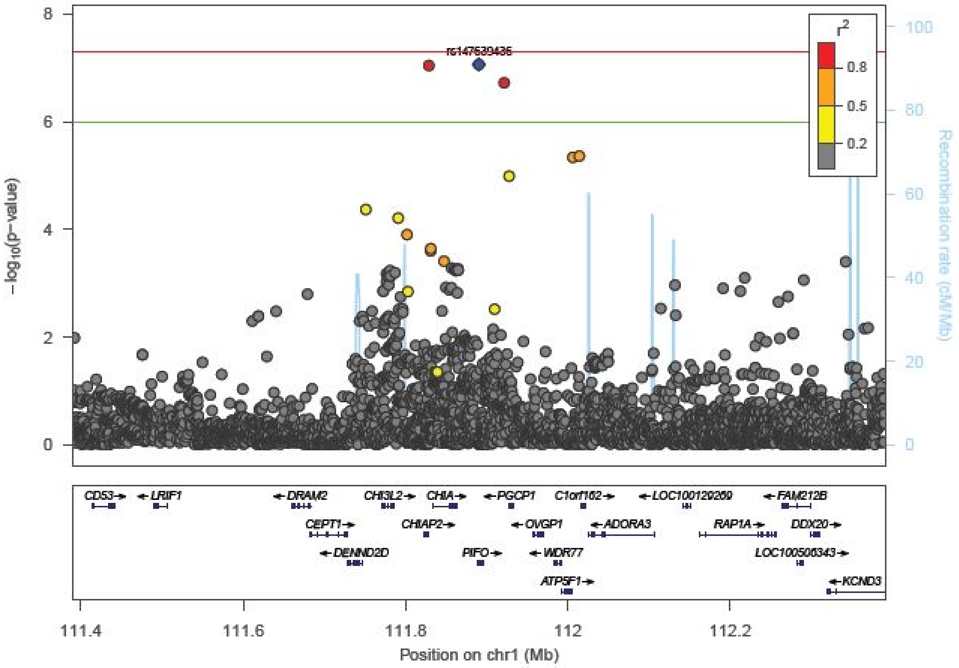

rs61816761 *FLG*

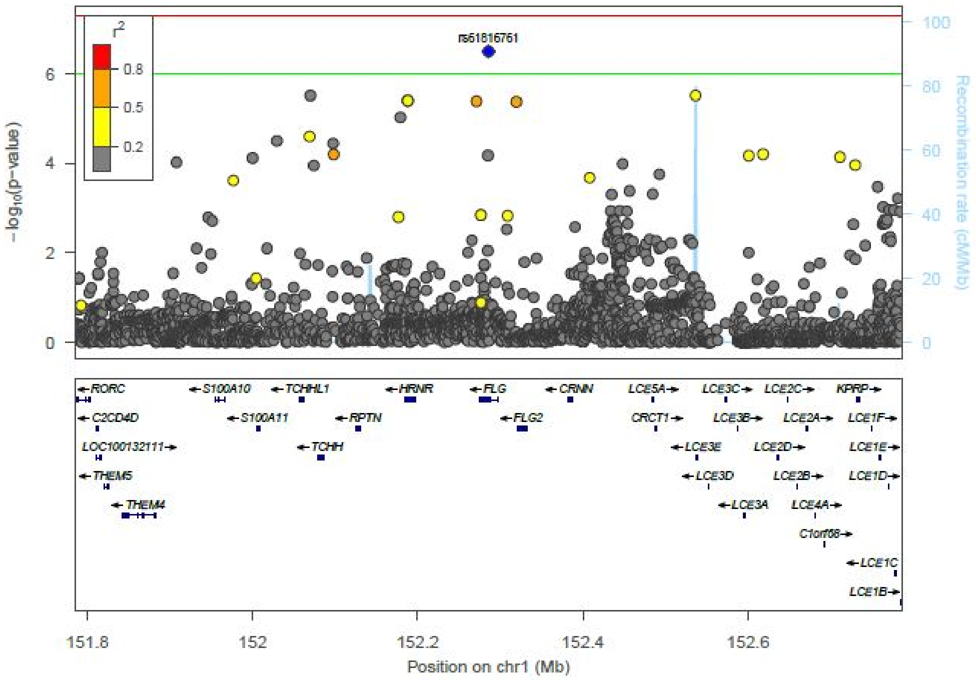

rs7523907 *CD247*

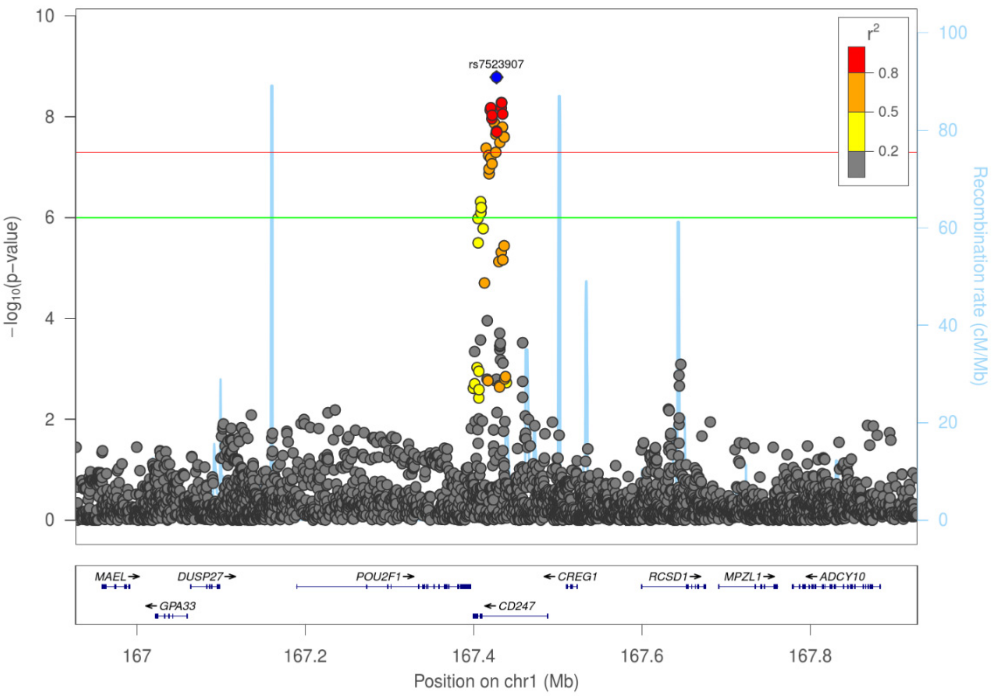

rs12479210 *IL1RL1*

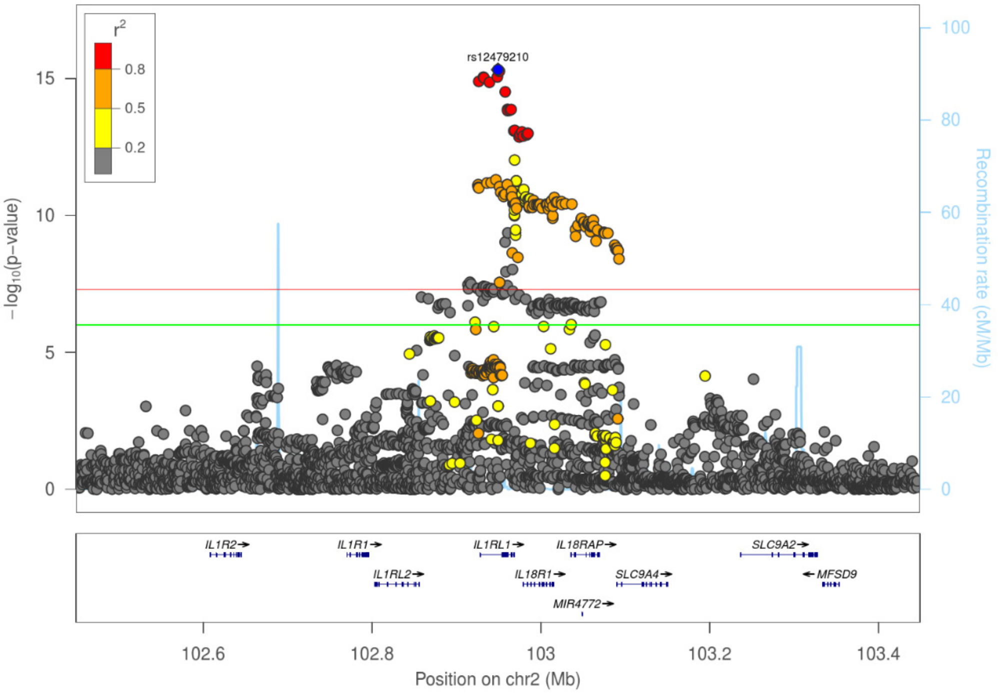

rs34290285 *D2HGDH*

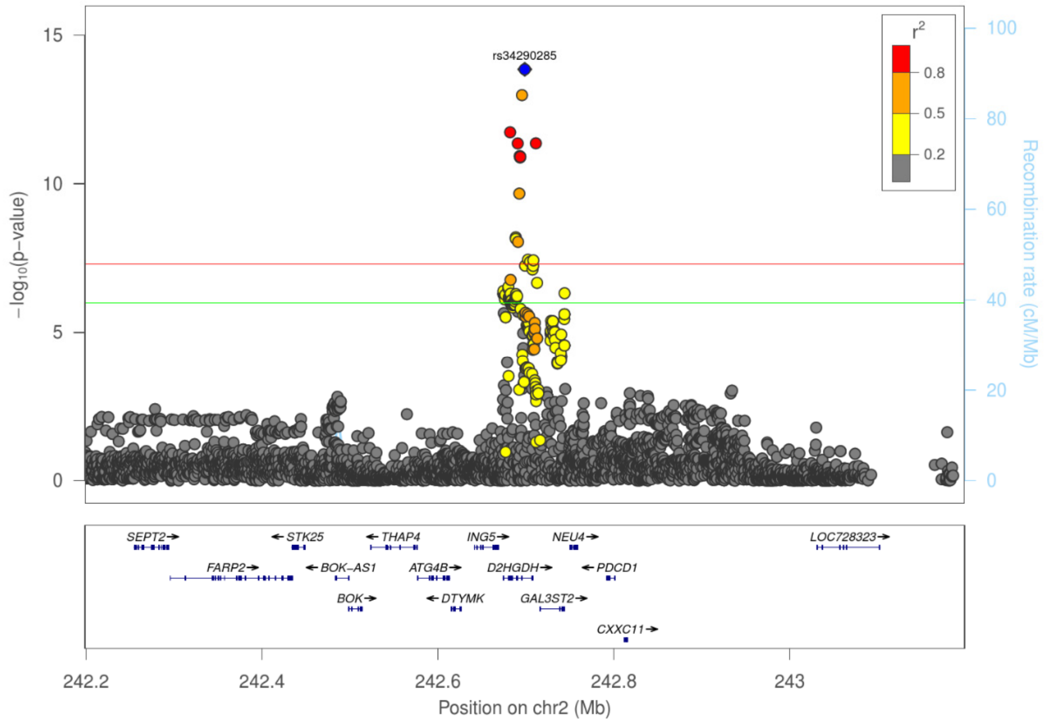

rs560026225 (4:123055701:G:GATT) *KIAA1109*

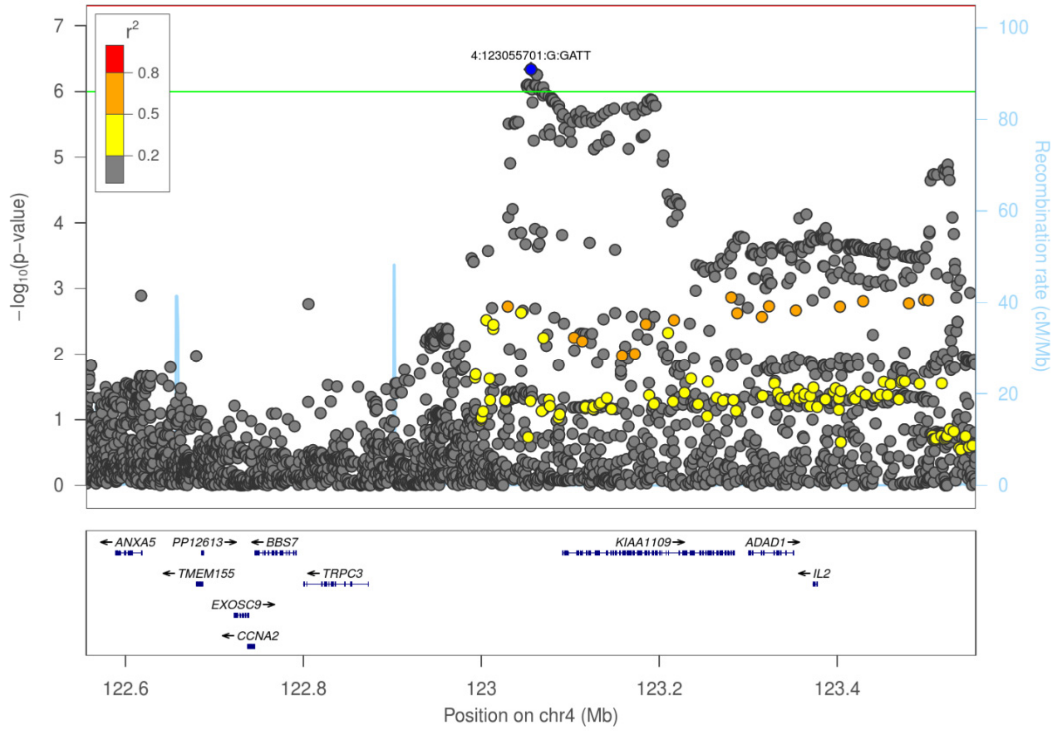

rs1837253 (Affx-25366072) *TSLP*

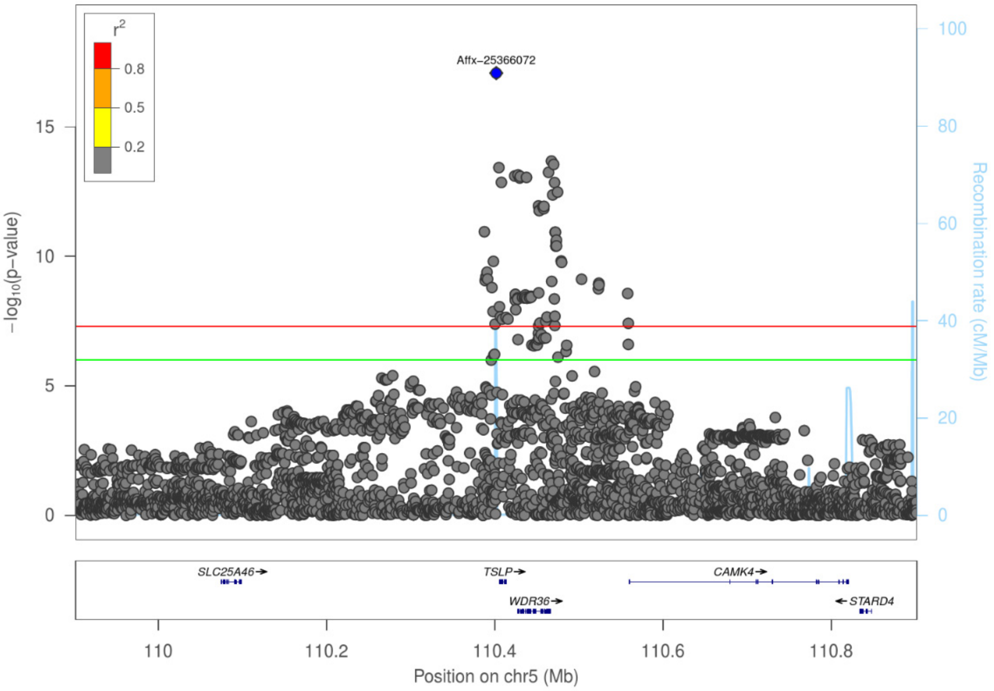

rs1438673 *WDR36* (conditioned on rs1837253)

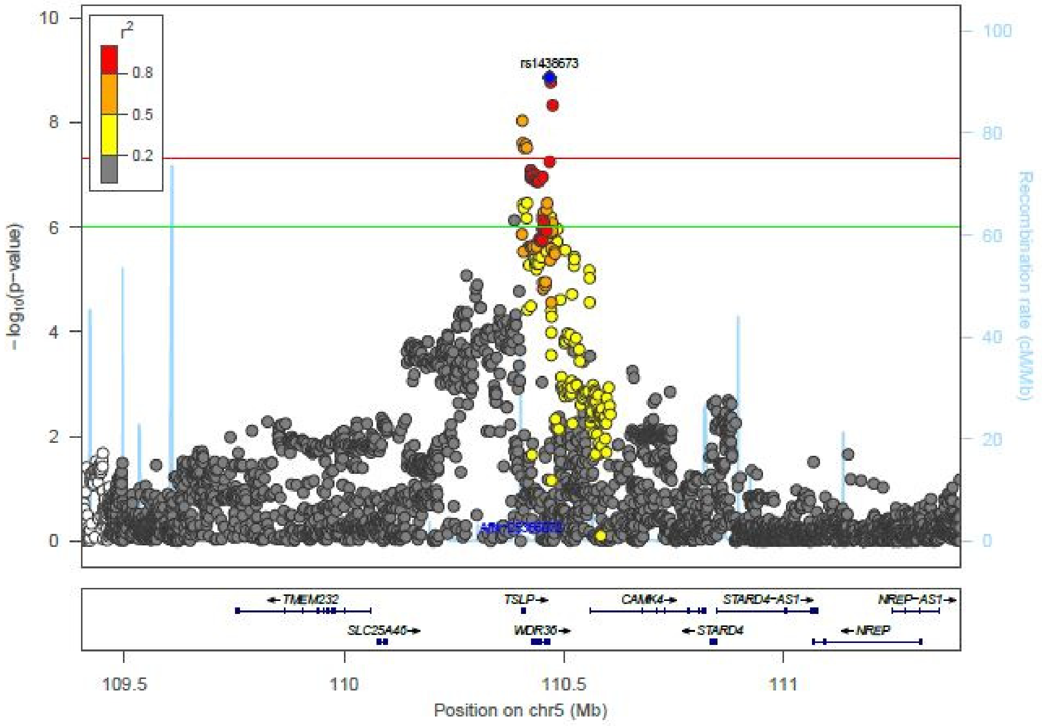

rs3749833 *C5orf56*

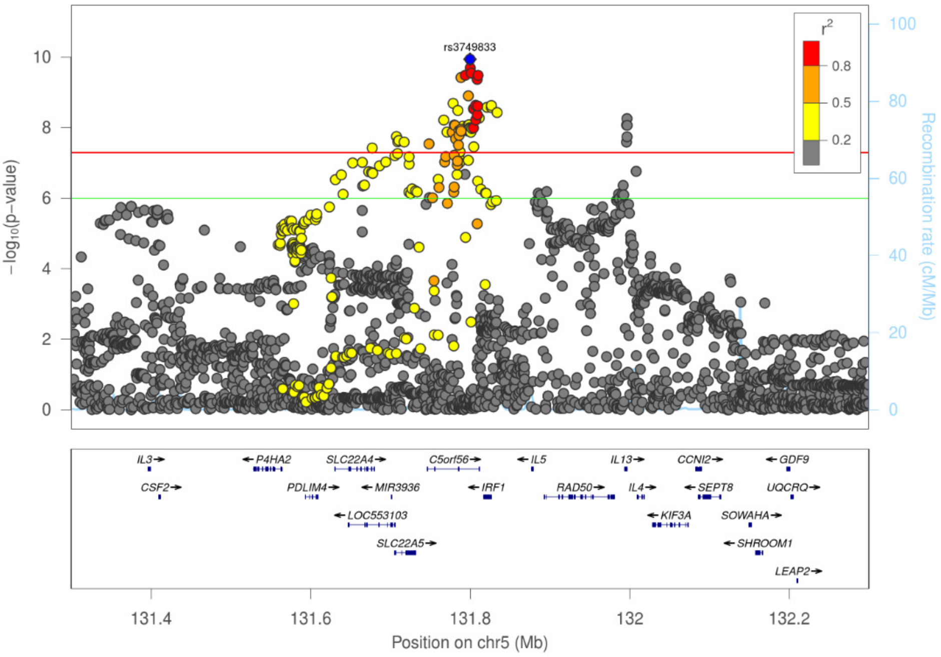

rs1986009 *RAD50* (conditioned on rs3749833)

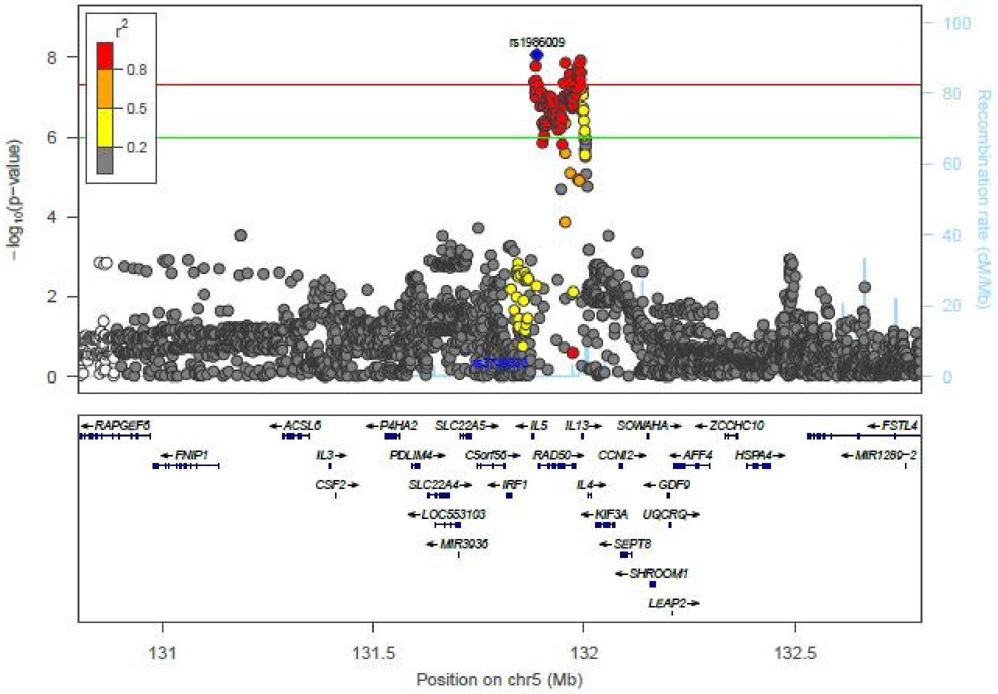

rs551660440 (5:180078030:G:A) *FLT4*

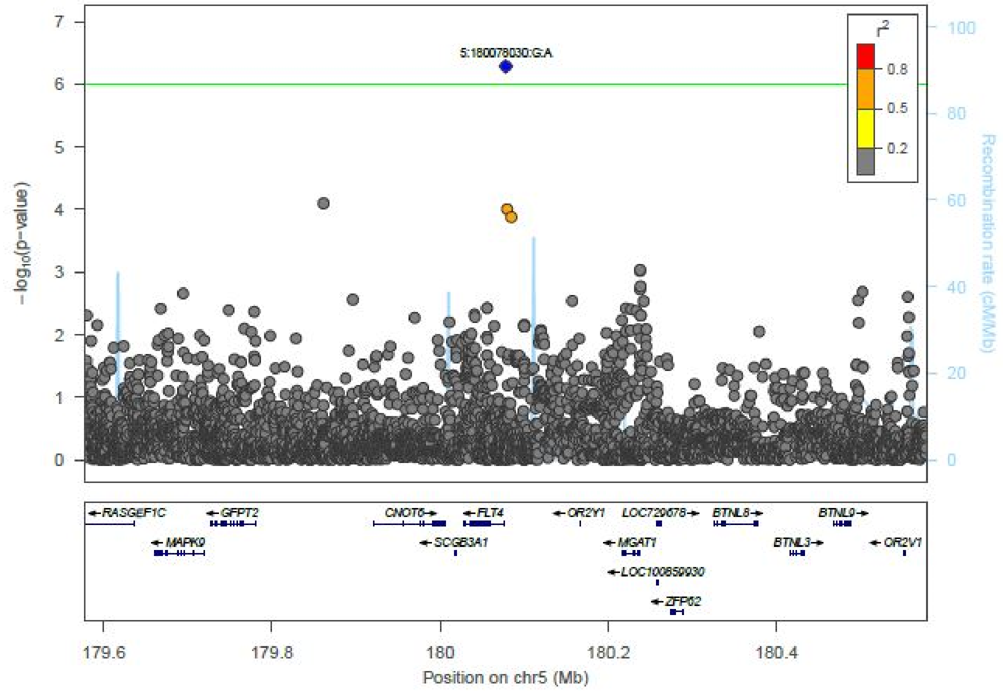

rs9273410 *HLA-DQB1*

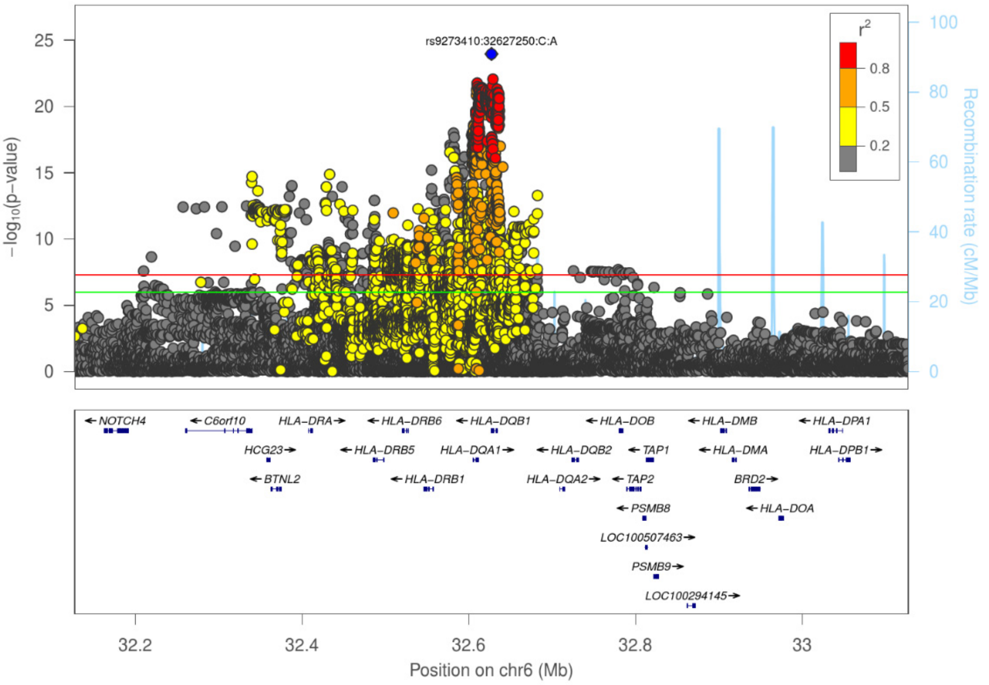

rs776111176 (chr6:32581739) *HLA-DQA1* (conditioned on rs9273410)

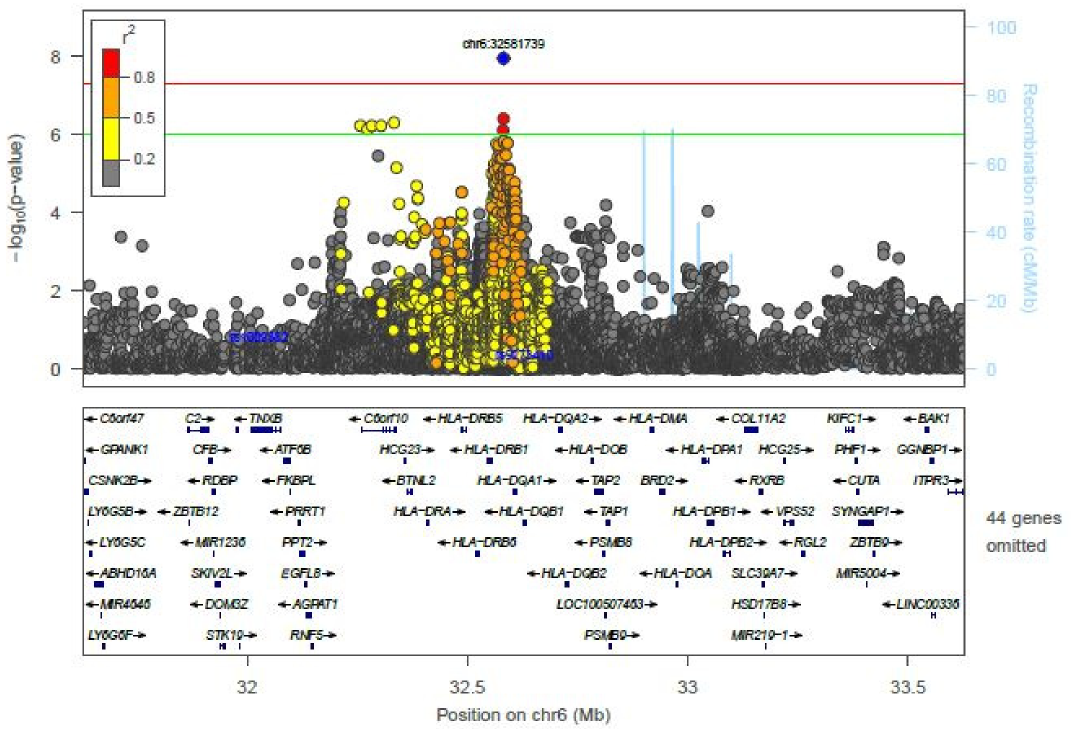

rs367983479 *BACH2*

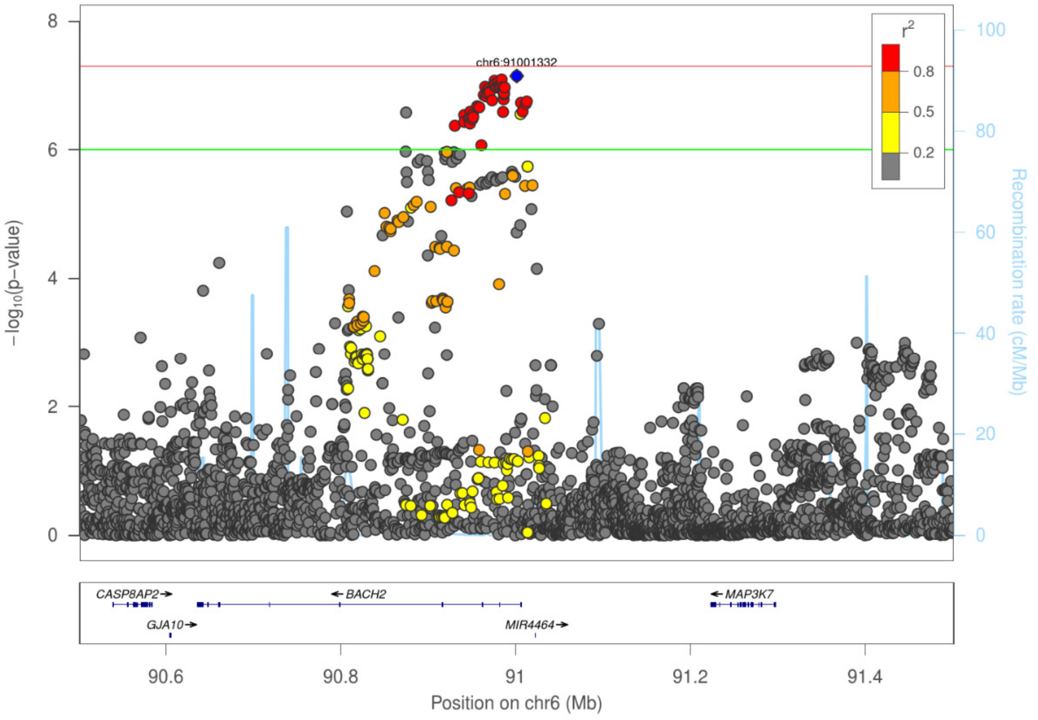

rs56192946 *TNS3*

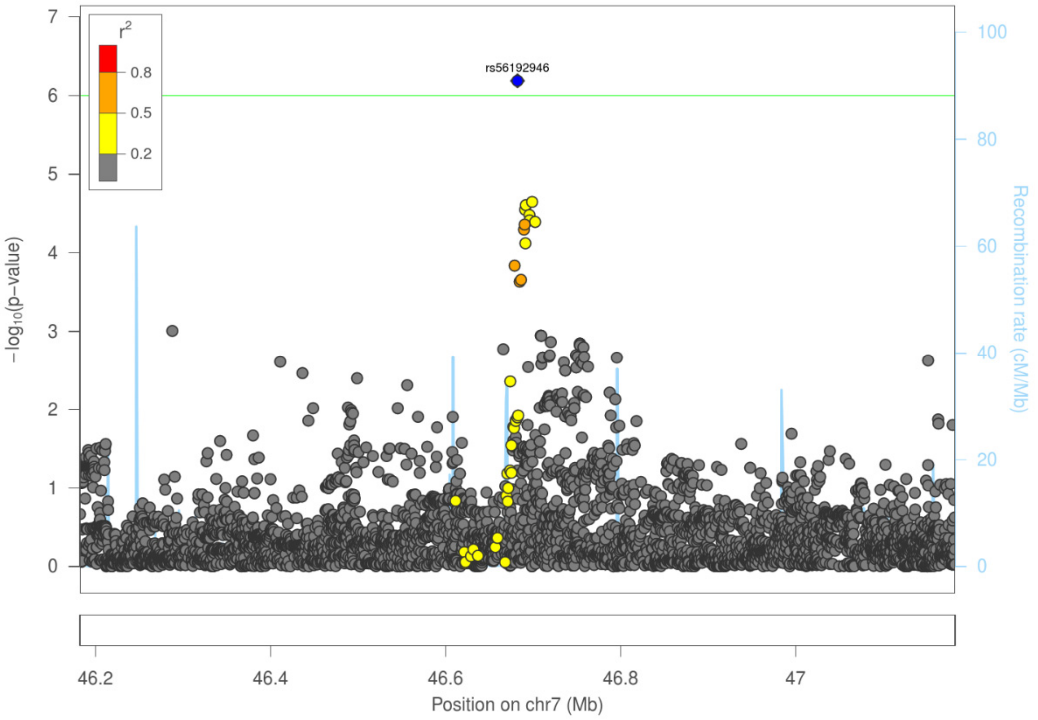

rs71266076 *MIR5708*

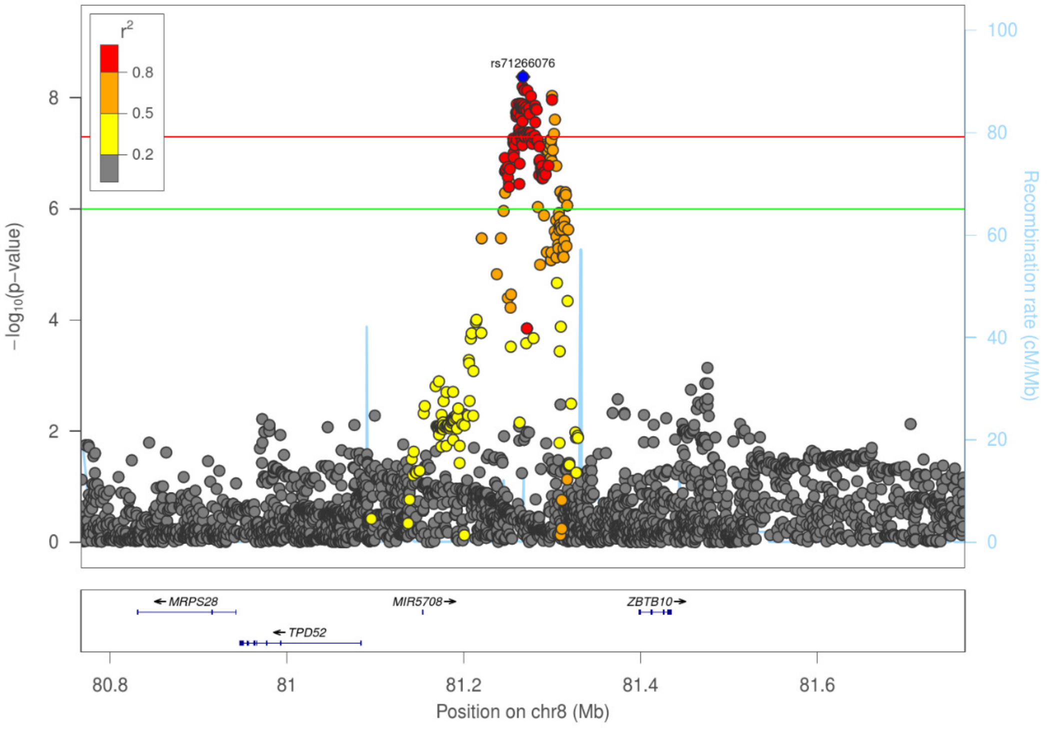

rs144829310 *IL33*

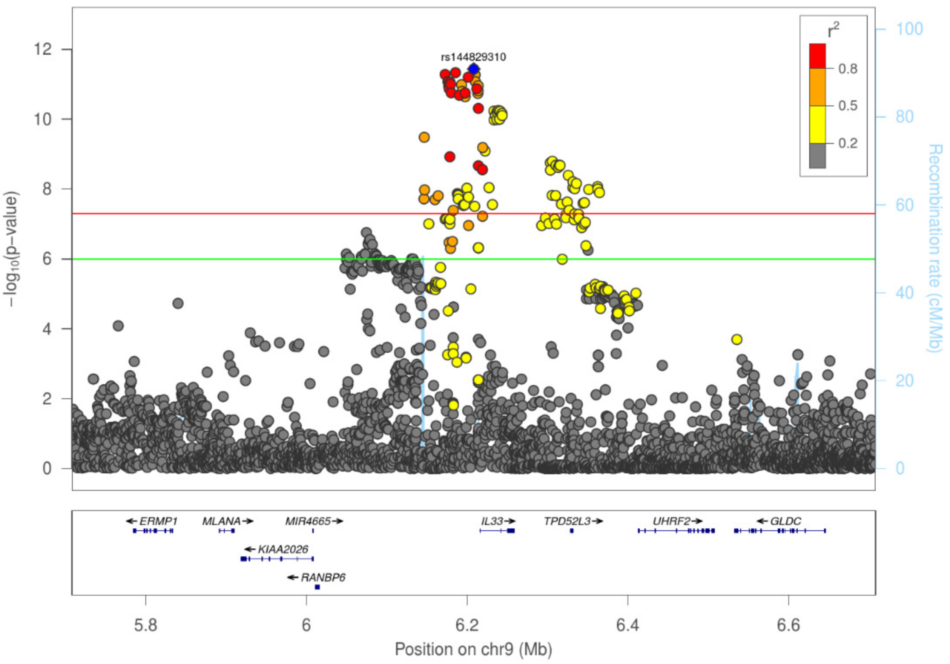

rs551372748 *CACNA1B*

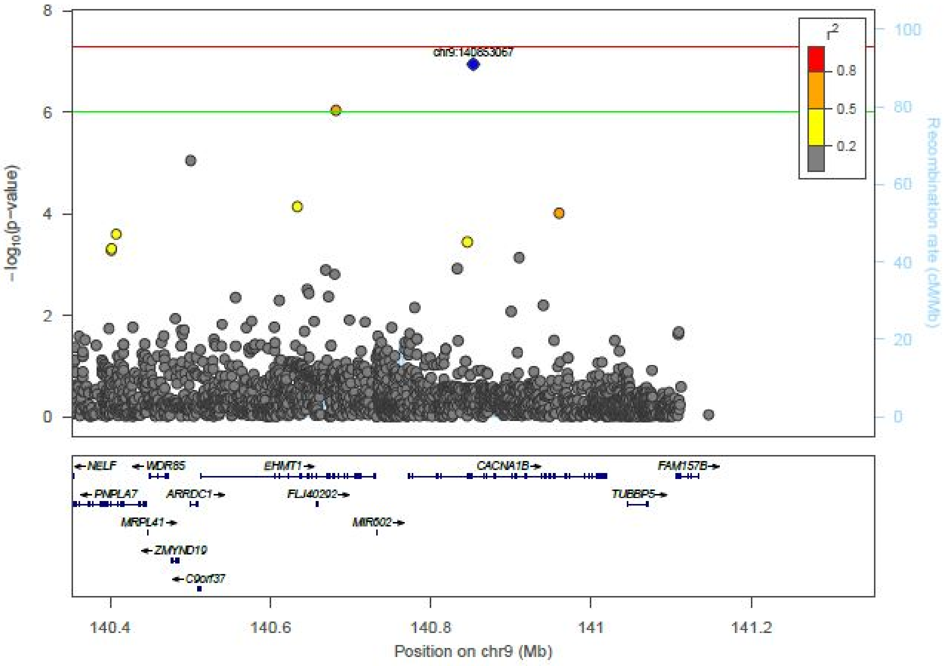

rs10905284 *GATA3*

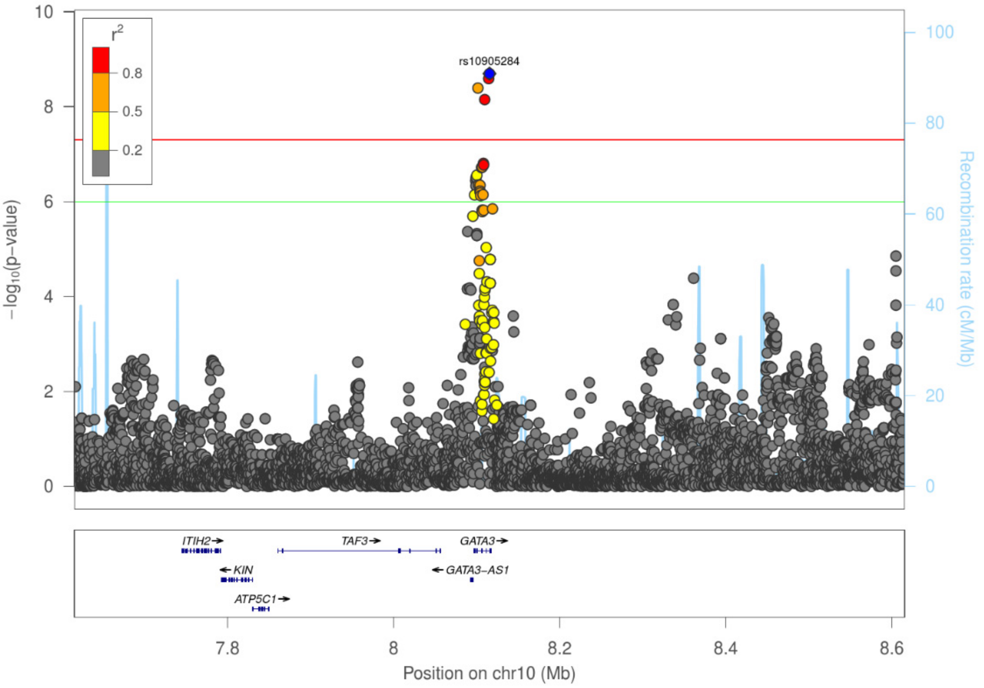

rs61840192 *LOC101928272*

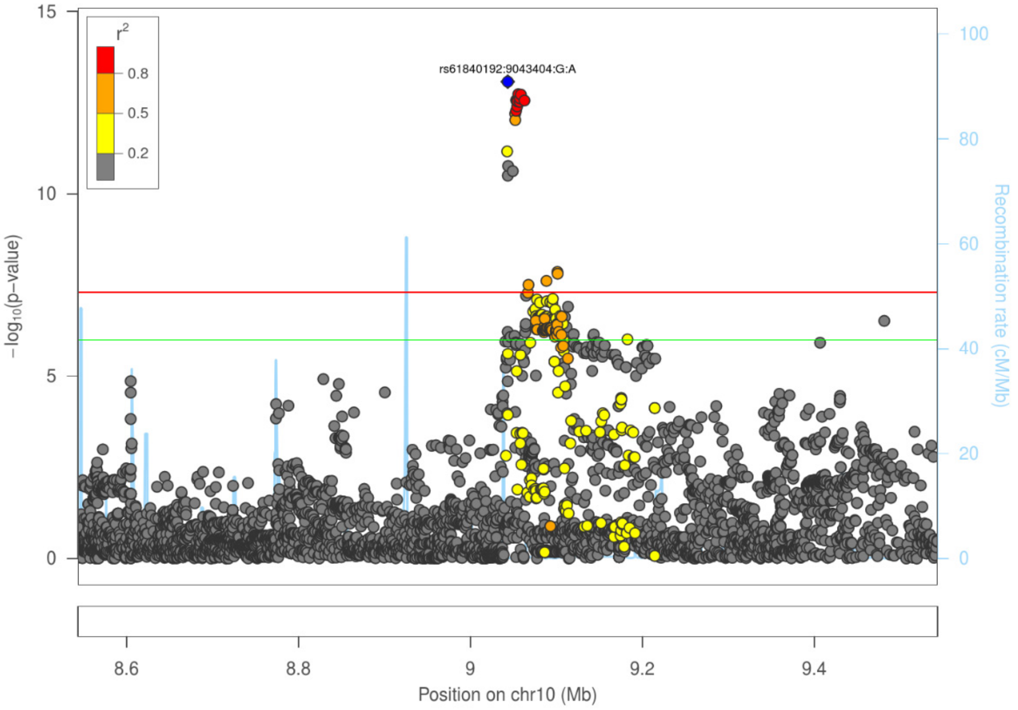

rs11603634 *MUC5AC*

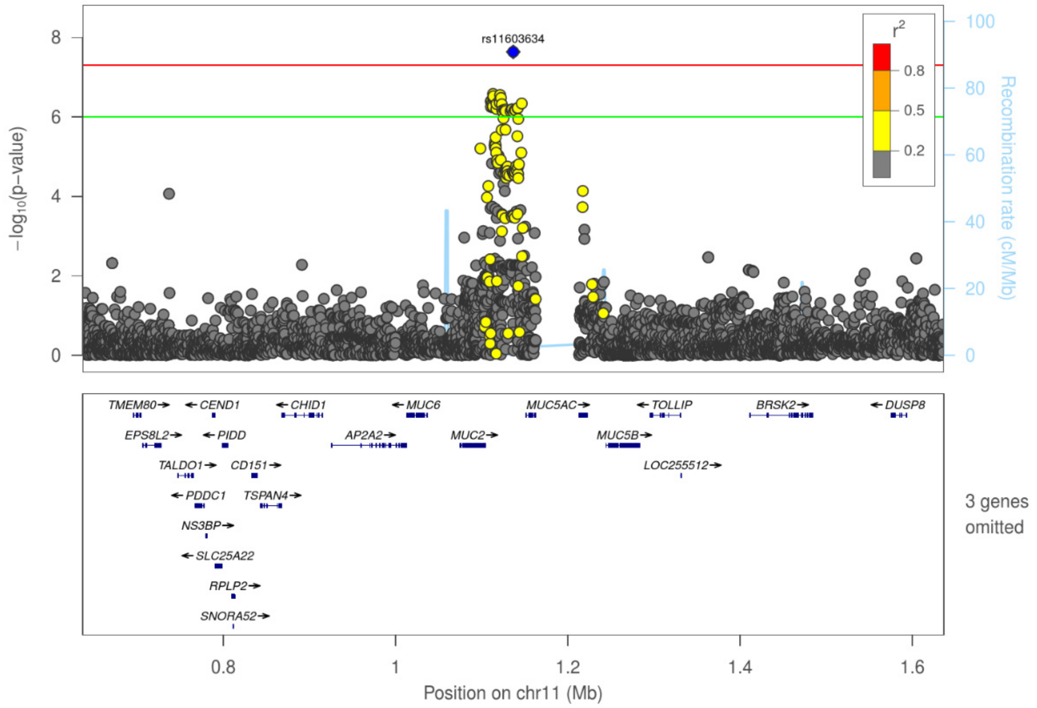

rs7936312 *C11orf30*

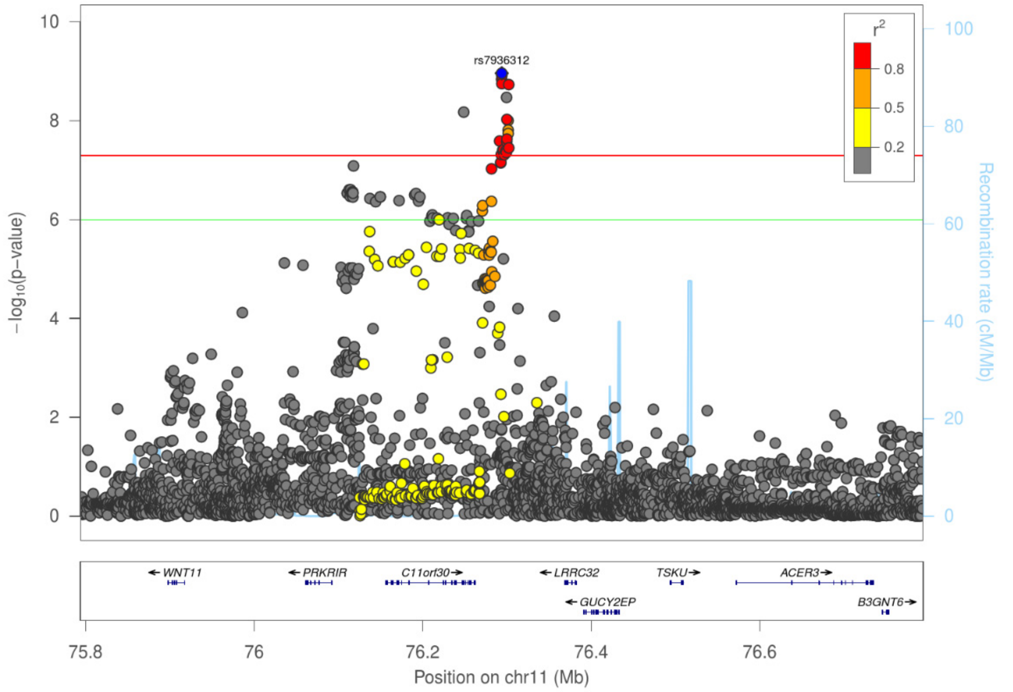

rs7305461 *RPS26*

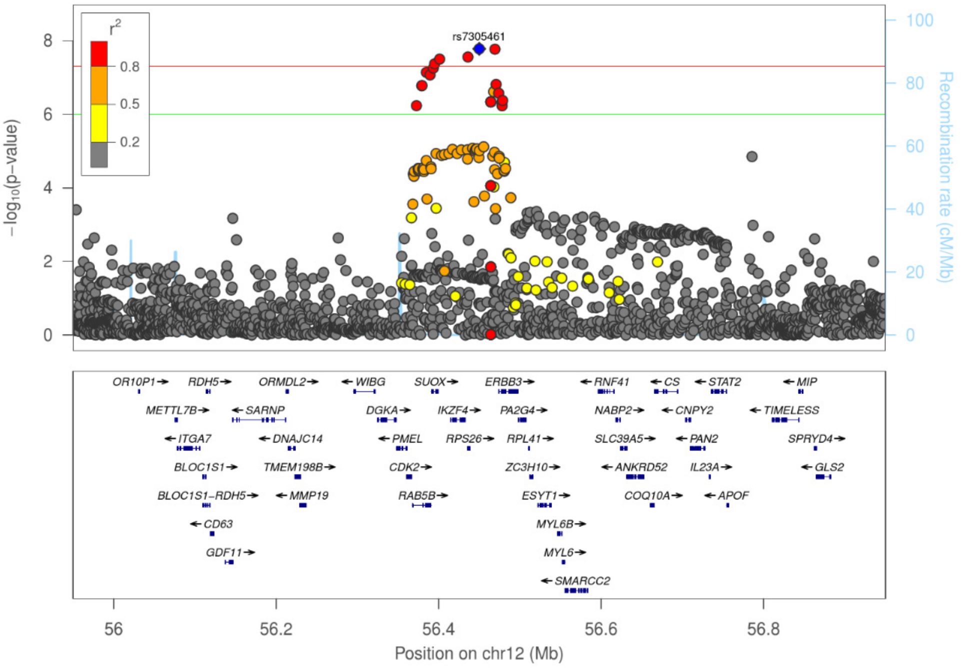

rs703816 *STAT6*

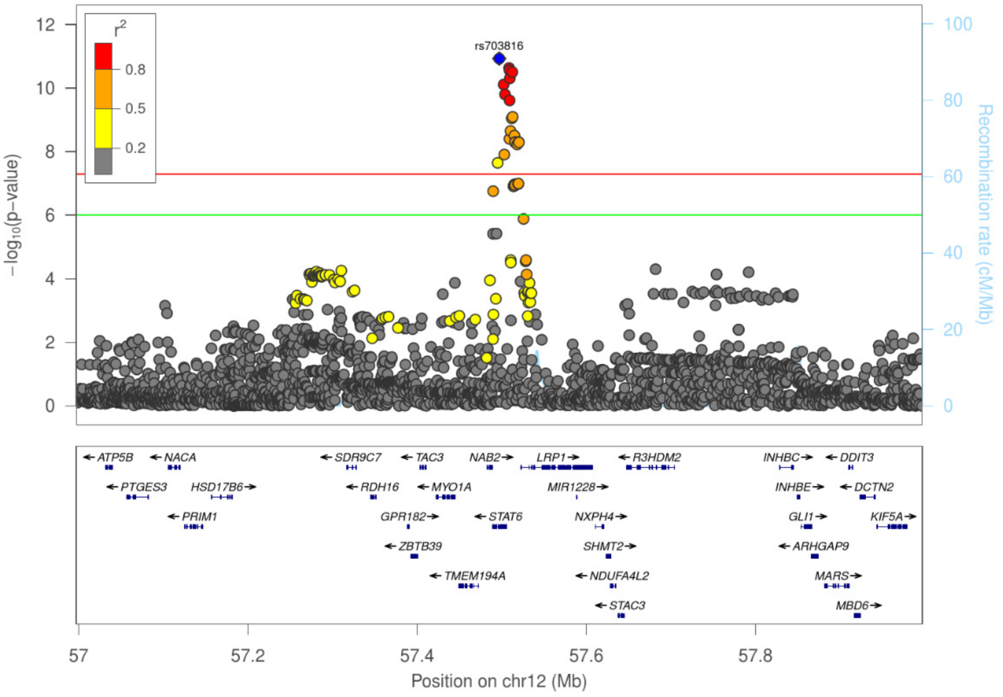

rs9517738 *LINC01232*

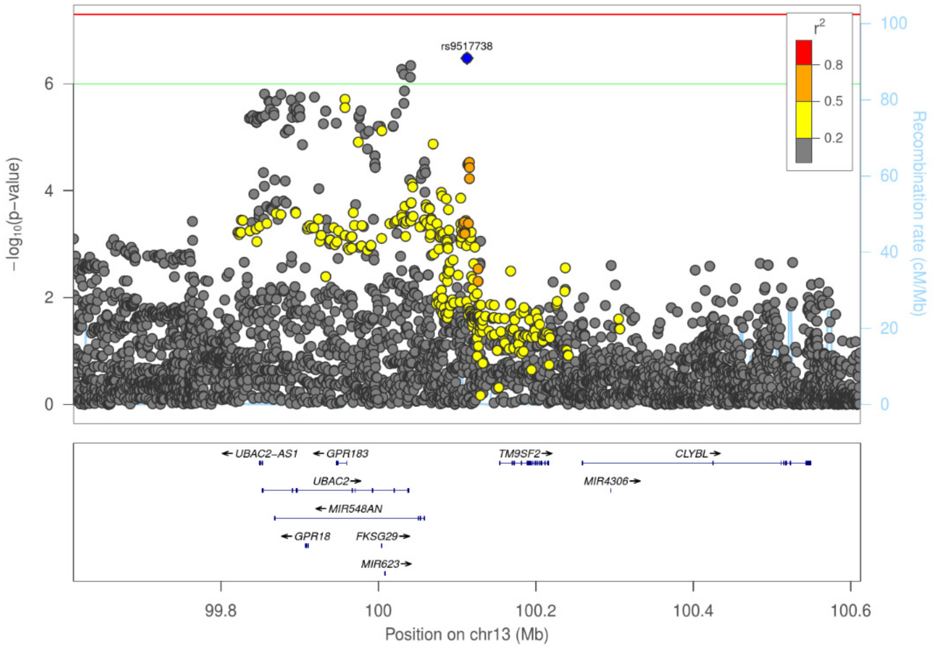

rs10519068 *RORA*

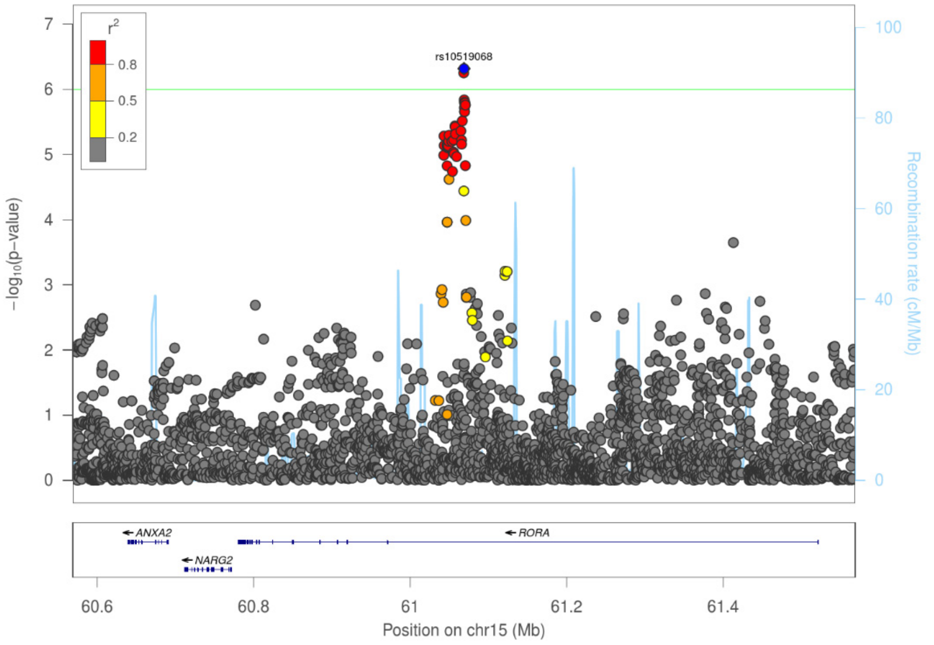

rs72743461 *SMAD3*

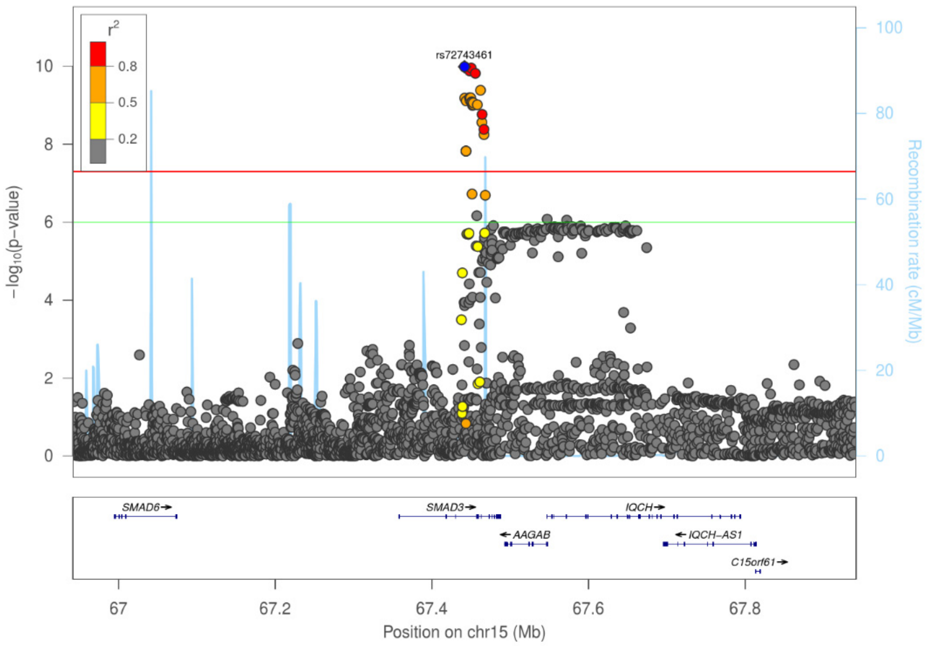

rs7203459 *CLEC16A*

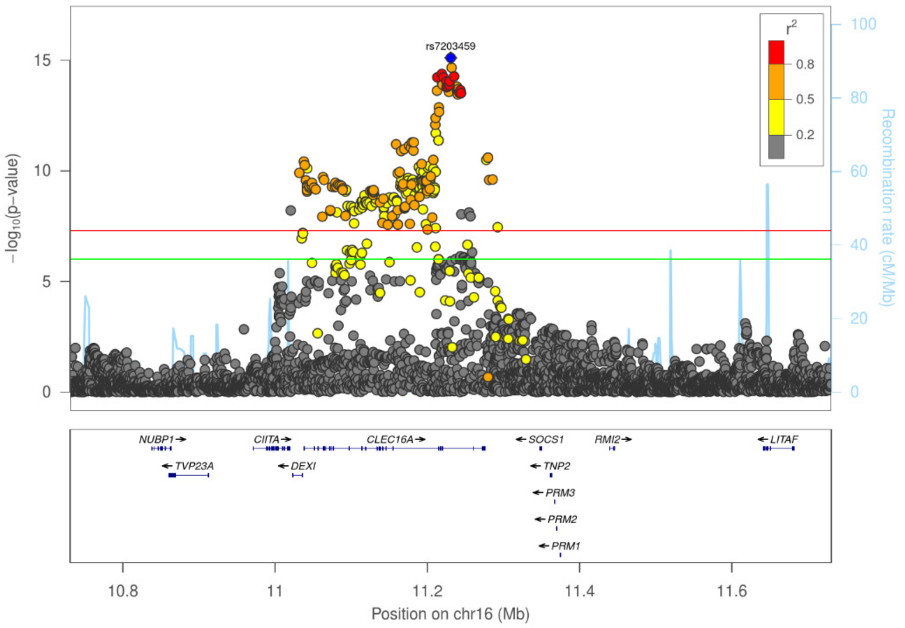

rs2941522 *IKZF3*

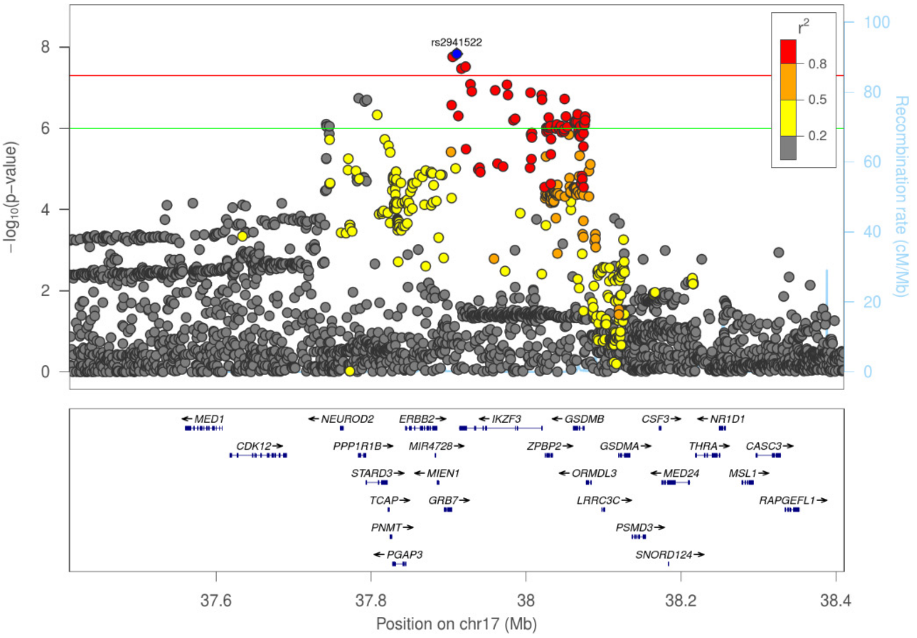

rs112502960 *ZNF652*

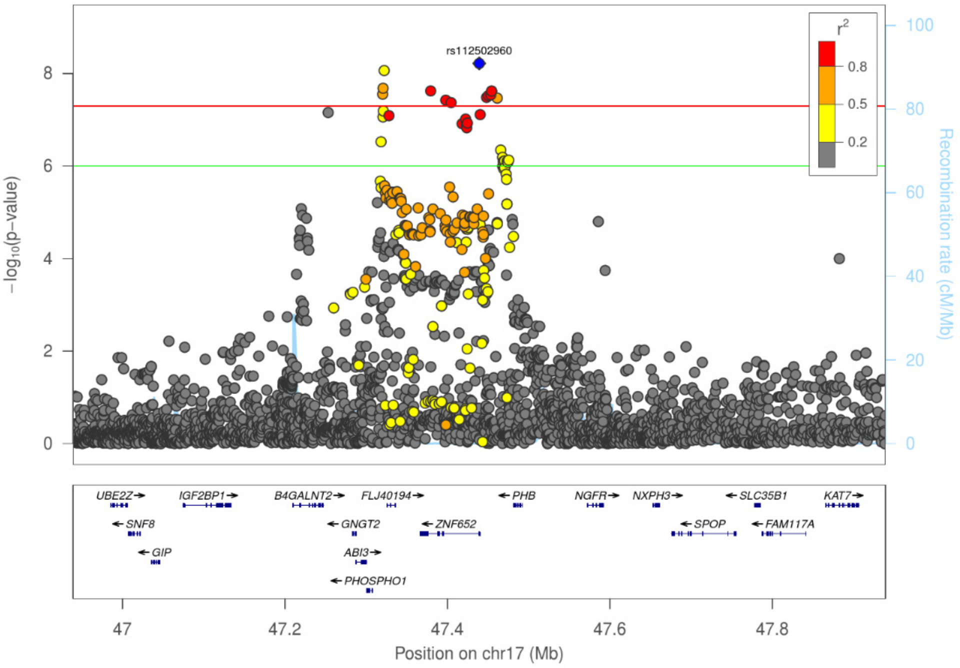

Supplementary Figure 4: rs11603634 shows a non-significant association with *MUC5B* mRNA expression in bronchial epithelial brush samples. Bronchial epithelial brush samples (n=117) were collected as part of the U-BIOPRED study, DNA was extracted and genotypes generated using the Affymetrix Axiom® UK Biobank array and imputation, RNA was extracted and transcriptomic analysis was performed using the Affymetrix HT HG-U133 1 PM GeneChip. rs11603634 was not directly genotyped, therefore proxy rs11602802 was used. The rs11603634 asthma risk allele (G) is correlated with rs11602802 (A) allele. The graph shows the data for MUC5B mRNA expression stratified by rs11602802 genotype, the box and whiskers show the mean and IQR for each genotype. P= 0.295 (FDR).

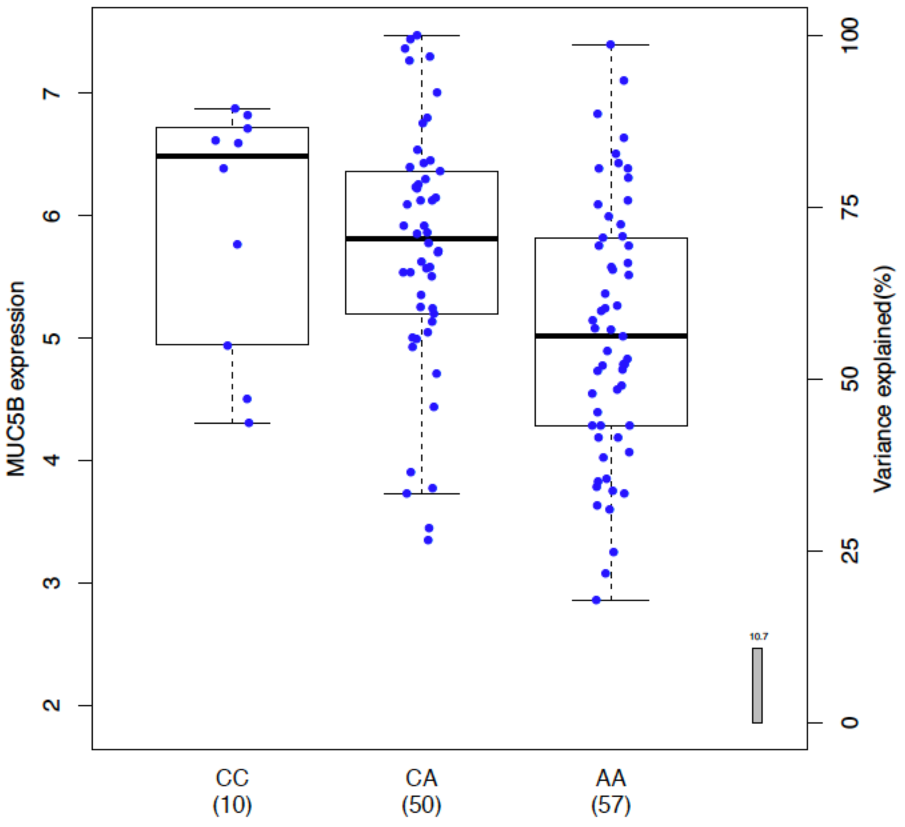

Supplementary Figure 5: KIA1109 expression in the human lung. Bronchial epithelial cells show low level expression that is cytoplasmic/membranous. Figure from Protein Atlas (see https://www.proteinatlas.org/ENSG00000138688-KIAA1109/tissue/bronchus#img).

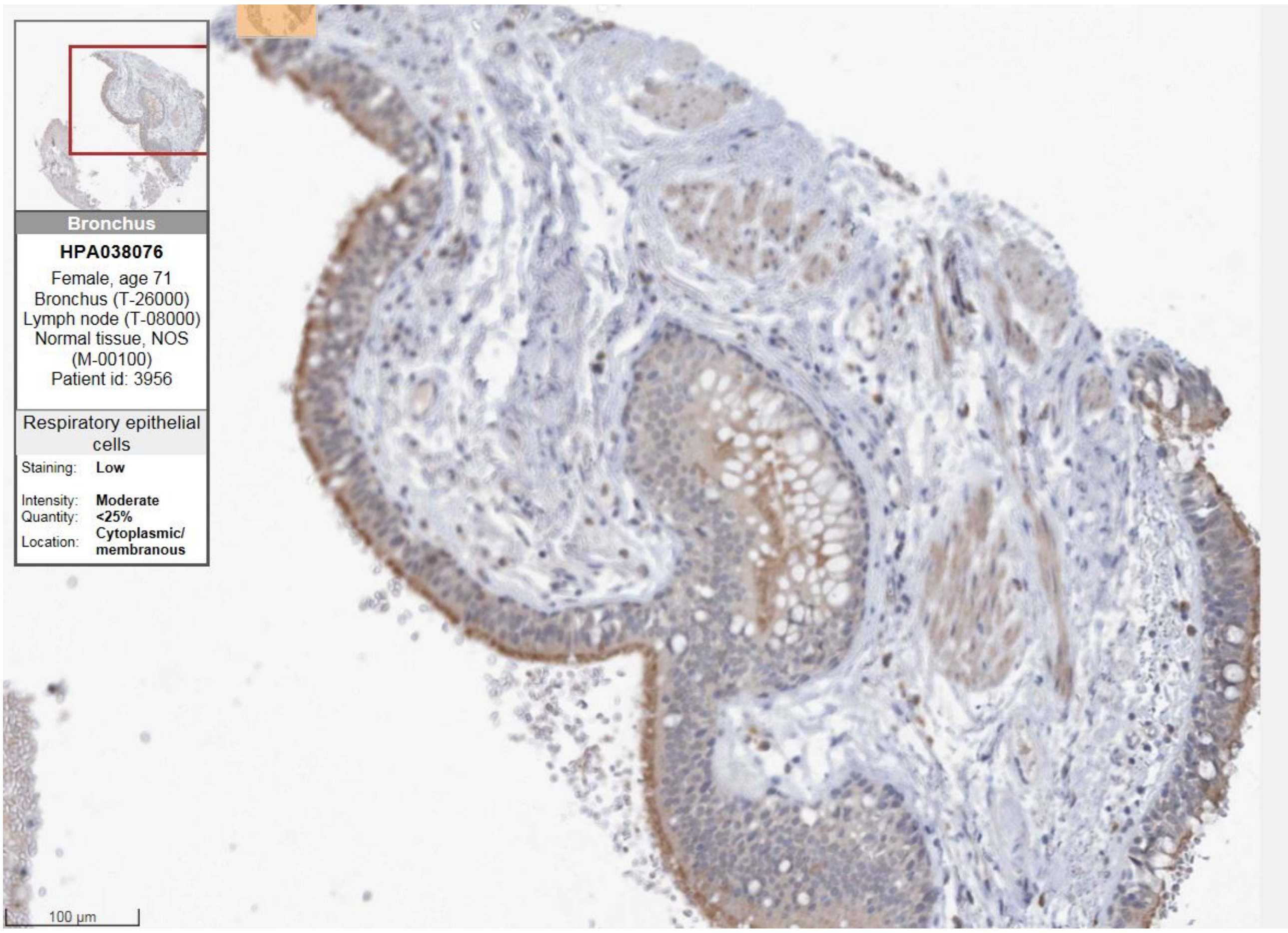

Supplementary Figure 6: MUC5AC expression in the human lung. Bronchial epithelial cells show high level expression that is cytoplasmic/membranous. Figure from Protein Atlas (see https://www.proteinatlas.org/ENSG00000215182-MUC5AC/tissue/bronchus#img).

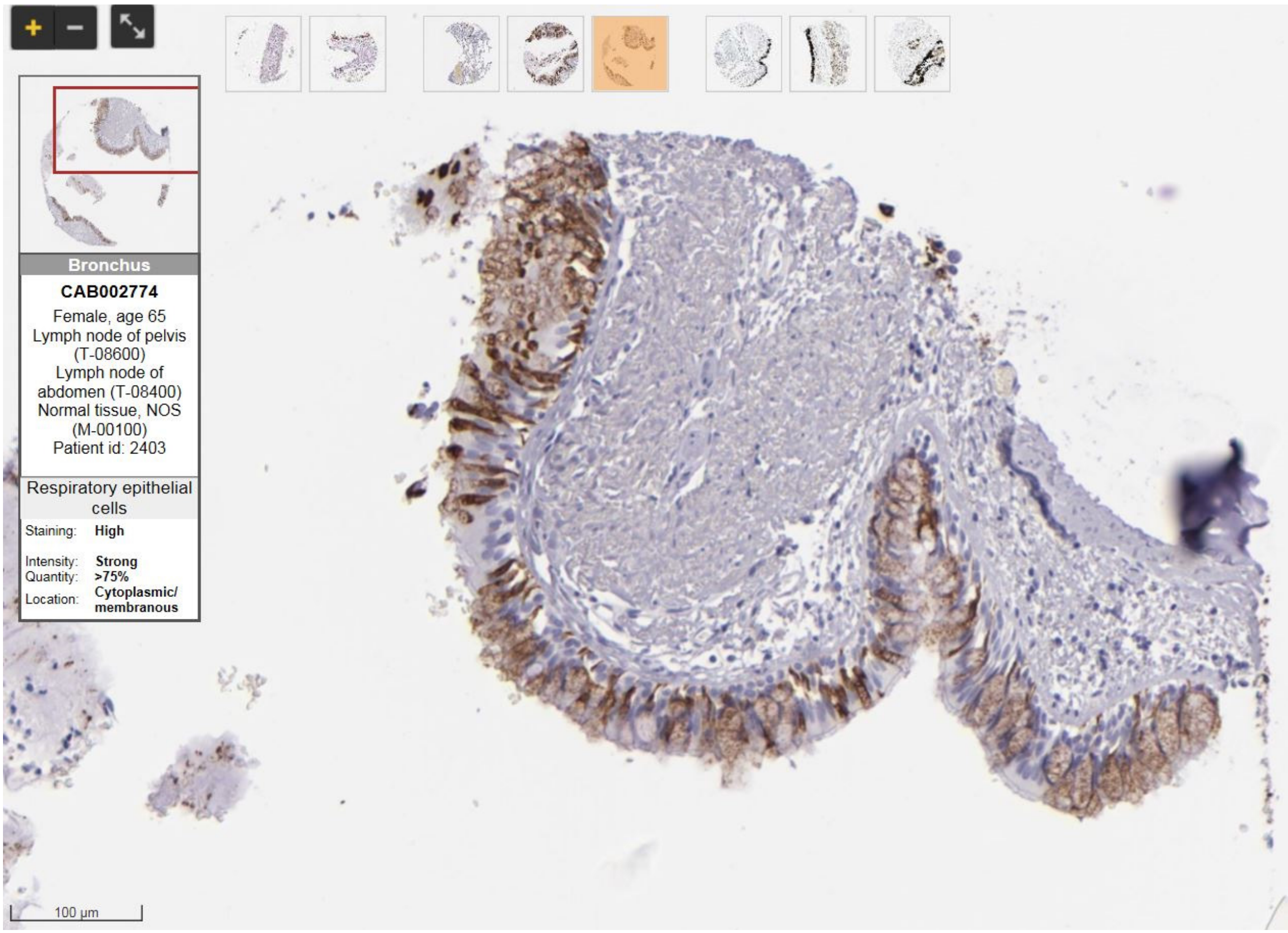

Supplementary Table 1: Summary of results for 32 variants associated with susceptibility to moderate-severe asthma in stage 1 discovery GWAS ($P < 10^{-6}$) and analysed in Stage 2 and Meta-analysis. In Stage 1 the P value is bold if it is genome-wide significant ($P < 5\times10^{-8}$; 21 variants). In the Stage 2 columns the OR is bold if there is a consistent direction of effect with stage 1 (30 variants) and the P value is bold if it is nominally significant ($P < 0.05$; 26 variants). The meta-analysis P value is bold if it is genome-wide significant ($P < 5\times10^{-8}$; 25 variants).

									**Stage 1**		**Stage 2**		**Meta**	
**Chr**	**Position**	**Variant**	**Locus**	**Non-coded**	**coded**	**Minor allele**	**condition**	**MAF**	**OR [95% CI]**	**P**	**OR [95% CI]**	**P**	**OR [95% CI]**	**P**
**Novel**														
1	8,501,786	rs301819	*RERE*	A	G	A		40.90%	1.12 [1.07-1.17]	2.84E-07	**1.05 [1.00-1.10]**	**2.89E-02**	1.08 [1.05-1.12]	2.66E-07
1	12,144,801	rs137907825	*TNFRSF8*	CTG	C	C		27.89%	1.13 [1.08-1.18]	8.78E-07	**1.03 [0.98-1.08]**	2.78E-01	1.08 [1.04-1.11]	2.38E-05
1	111,890,568	rs147639436	*PIFO*	A	G	G		1.43%	1.67 [1.38-2.02]	1.31E-07	0.96 [0.80-1.16]	6.94E-01	1.25 [1.10-1.43]	7.43E-04
1	152,285,861	rs61816761	*FLG*	G	A	A		2.37%	1.50 [1.28-1.76]	4.70E-07	**1.26 [1.09-1.45]**	**2.18E-03**	1.36 [1.22-1.51]	**1.18E-08**
4	123,055,701	rs560026225	*KIAA1109*	G	GATT	GATT		23.60%	1.15 [1.09-1.21]	4.62E-07	**1.09 [1.04-1.15]**	**7.50E-04**	1.12 [1.08-1.16]	**3.06E-09**
5	180,078,030	rs551660440	*FLT4*	G	A	A		0.79%	2.08 [1.56-2.79]	7.46E-07	**1.01 [0.80-1.28]**	9.31E-01	1.35 [1.12-1.62]	1.45E-03
7	46,682,176	rs56192946	*TNS3*	C	T	T		18.27%	0.87 [0.82-0.92]	6.50E-07	**0.97 [0.92-1.03]**	3.25E-01	0.92 [0.88-0.96]	3.22E-05
9	140,853,067	rs551372748	*CACNA1B*	A	G	G		0.72%	2.34 [1.70-3.22]	1.72E-07	0.90 [0.67-1.20]	4.67E-01	1.39 [1.12-1.72]	2.80E-03
10	8,115,362	rs10905284	*GATA3*	C	A	C		42.94%	0.87 [0.84-0.91]	**2.01E-09**	**0.94 [0.90-0.98]**	**2.25E-03**	0.90 [0.88-0.93]	**1.76E-10**
11	1,136,478	rs11603634	*MUC5AC*	A	G	A		49.64%	1.13 [1.08-1.18]	**2.30E-08**	**1.05 [1.01-1.10]**	**1.82E-02**	1.09 [1.06-1.12]	**2.32E-08**
13	100,112,522	rs9517738	*LINC01232*	G	A	G		29.76%	1.13 [1.08-1.19]	3.32E-07	**1.04 [0.99-1.09]**	1.31E-01	1.08 [1.05-1.12]	3.24E-06
**Previous**														
1	167,427,247	rs7523907	*CD247*	C	T	C		45.92%	1.14 [1.10-1.20]	**1.64E-09**	**1.05 [1.01-1.10]**	**2.11E-02**	1.10 [1.06-1.13]	**4.82E-09**
2	102,949,161	rs12479210	*IL1RL1*	C	T	T		38.73%	1.20 [1.15-1.26]	**4.77E-16**	**1.19 [1.14-1.24]**	**4.82E-15**	1.19 [1.16-1.23]	**1.57E-29**
2	242,698,640	rs34290285	*D2HGDH*	G	A	A		25.74%	0.82 [0.78-0.87]	**1.41E-14**	**0.85 [0.81-0.89]**	**1.16E-10**	0.84 [0.81-0.87]	**2.24E-23**
5	110,401,872	rs1837253	*TSLP*	T	C	T		25.84%	1.24 [1.18-1.30]	**8.49E-18**	**1.14 [1.08-1.20]**	**1.75E-07**	1.19 [1.15-1.23]	**1.95E-22**
5	110,467,499	rs1438673	*WDR36*	C	T	T	rs1837253	49.22%	0.87 [0.84-0.91]	**2.35E-09**	**0.91 [0.87-0.95]**	**1.33E-05**	0.89 [0.86-0.92]	**3.29E-13**
5	131,799,626	rs3749833	*C5orf56*	T	C	C		26.08%	1.17 [1.12-1.23]	**1.14E-10**	**1.11 [1.06-1.16]**	**2.69E-05**	1.14 [1.10-1.18]	**5.60E-14**
5	131,887,986	rs1986009	*RAD50*	C	A	A	rs3749833	18.71%	1.18 [1.11-1.24]	**1.39E-08**	**1.16 [1.10-1.23]**	**4.11E-08**	1.17 [1.13-1.22]	**2.43E-15**
6	32,581,739	rs776111176	*HLA-DQA1*	A	AAT	A	rs9273410	14.85%	0.82 [0.77-0.88]	**1.81E-08**	**0.85 [0.81-0.90]**	**2.62E-09**	0.84 [0.81-0.88]	**2.61E-16**
6	32,627,250	rs9273410	*HLA-DQB1*	C	A	C		44.70%	1.26 [1.20-1.32]	**1.07E-24**	**1.16 [1.11-1.21]**	**2.14E-10**	1.21 [1.17-1.25]	**5.62E-32**
6	91,001,332	rs367983479	*BACH2*	CA	C	C		38.50%	0.88 [0.85-0.93]	7.12E-08	**0.92 [0.88-0.96]**	**1.12E-04**	0.90 [0.87-0.93]	**6.30E-11**
8	81,266,924	rs71266076	*MIR5708*	C	CT	C		36.93%	0.87 [0.83-0.91]	**4.21E-09**	**0.91 [0.87-0.95]**	**1.57E-05**	0.89 [0.86-0.92]	**6.53E-13**
9	6,208,030	rs144829310	*IL33*	G	T	T		16.40%	1.23 [1.16-1.30]	**3.68E-12**	**1.19 [1.13-1.26]**	**1.12E-09**	1.21 [1.16-1.26]	**2.29E-20**
10	9,043,404	rs61840192	*LOC101928272*	G	A	A		42.70%	0.85 [0.81-0.88]	**8.42E-14**	**0.86 [0.82-0.89]**	**1.14E-12**	0.85 [0.83-0.88]	**8.33E-25**
11	76,293,726	rs7936312	*C11orf30*	G	T	T		47.42%	1.14 [1.10-1.19]	**1.09E-09**	**1.19 [1.14-1.24]**	**3.38E-16**	1.17 [1.13-1.20]	**6.18E-24**
12	56,449,875	rs7305461	*RPS26*	A	C	A		44.61%	0.88 [0.84-0.92]	**1.65E-08**	**0.94 [0.90-0.98]**	**2.51E-03**	0.91 [0.88-0.94]	**1.01E-09**
12	57,497,005	rs703816	*STAT6*	T	C	C		43.41%	1.16 [1.11-1.21]	**1.18E-11**	**1.08 [1.03-1.13]**	**4.31E-04**	1.12 [1.09-1.15]	**3.69E-13**
15	61,068,704	rs10519068	*RORA*	G	A	A		12.75%	0.85 [0.79-0.90]	4.81E-07	**0.85 [0.79-0.90]**	**5.76E-07**	0.85 [0.81-0.89]	**1.84E-12**
15	67,441,750	rs72743461	*SMAD3*	C	A	A		23.60%	1.18 [1.12-1.24]	**1.03E-10**	**1.11 [1.06-1.17]**	**2.35E-05**	1.14 [1.11-1.19]	**4.52E-14**
16	11,230,703	rs7203459	*CLEC16A*	T	C	C		24.56%	0.81 [0.77-0.86]	**7.83E-16**	**0.90 [0.85-0.94]**	**2.33E-05**	0.86 [0.83-0.89]	**4.37E-18**
17	37,910,368	rs2941522	*IKZF3*	C	T	T		48.29%	1.13 [1.08-1.18]	**1.46E-08**	**1.10 [1.05-1.14]**	**1.97E-05**	1.11 [1.08-1.15]	**2.32E-12**
17	47,439,302	rs112502960	*ZNF652*	G	A	A		35.92%	1.14 [1.09-1.20]	**6.05E-09**	**1.08 [1.04-1.13]**	**3.92E-04**	1.11 [1.08-1.15]	**4.12E-11**

Supplementary Table 2: Summary of results for stage 2a sensitivity analysis (controls with atopy) for variants associated with susceptibility to moderate-severe asthma in stage 1 discovery GWAS ($P < 10^{-6}$). In the Stage 2a columns the OR is bold if there is a consistent direction of effect with stage 1 (30 variants) and the P value is bold if it is nominally significant (P <0.05; 25 variants). The meta-analysis P value is bold if it is genome-wide significant ($P < 5\times10^{-8}$; 24 variants).

										**Stage 2a**		**Meta-analysis**	
**Chr**	**Position**	**Variant**	**Locus**	**Non-coded**	**coded**	**Minor allele**	**condition**	**MAF**	**rsid.ukb (proxy r^2)**	**OR [95% CI]**	**P**	**OR [95% CI]**	**P**
**Novel**													
1	8,501,786	rs301819	*RERE*	A	G	A		40.90%	rs301819	**1.04 [1.00-1.09]**	6.85E-02	1.08 [1.05-1.11]	1.15E-06
1	12,144,801	rs137907825	*TNFRSF8*	CTG	C	C		27.89%	rs12133231 (1.00)	**1.03 [0.98-1.08]**	2.18E-01	1.08 [1.04-1.11]	1.68E-05
1	111,890,568	rs147639436	*PIFO*	A	G	G		1.43%	rs147639436	0.95 [0.79-1.13]	5.68E-01	1.24 [1.09-1.41]	1.39E-03
1	152,285,861	rs61816761	*FLG*	G	A	A		2.37%	rs61816761	**1.19 [1.04-1.37]**	**1.17E-02**	1.32 [1.19-1.46]	1.81E-07
4	123,055,701	rs560026225	*KIAA1109*	G	GATT	GATT		23.60%	rs72687036 (0.66)	**1.08 [1.02-1.13]**	**3.54E-03**	1.11 [1.07-1.15]	**2.59E-08**
5	180,078,030	rs551660440	*FLT4*	G	A	A		0.79%	rs143214597 (0.70)	**0.98 [0.78-1.23]**	8.42E-01	1.31 [1.09-1.57]	3.51E-03
7	46,682,176	rs56192946	*TNS3*	C	T	T		18.27%	rs56192946	**0.97 [0.92-1.03]**	3.07E-01	0.92 [0.89-0.96]	3.23E-05
9	140,853,067	rs551372748	*CACNA1B*	A	G	G		0.72%	rs551372748	0.93 [0.70-1.24]	6.30E-01	1.40 [1.13-1.73]	1.78E-03
10	8,115,362	rs10905284	*GATA3*	C	A	C		42.94%	rs10905284	**0.94 [0.90-0.98]**	**3.69E-03**	0.91 [0.88-0.94]	**4.15E-10**
11	1,136,478	rs11603634	*MUC5AC*	A	G	A		49.64%	rs11603634	**1.05 [1.01-1.09]**	**2.54E-02**	1.09 [1.06-1.12]	**4.48E-08**
13	100,112,522	rs9517738	*LINC01232*	G	A	G		29.76%	rs9517738	**1.03 [0.98-1.08]**	2.38E-01	1.08 [1.04-1.11]	1.14E-05
**Previous**													
1	167,427,247	rs7523907	*CD247*	C	T	C		45.92%	rs7523907	**1.05 [1.00-1.09]**	**3.65E-02**	1.09 [1.06-1.12]	**1.46E-08**
2	102,949,161	rs12479210	*IL1RL1*	C	T	T		38.73%	rs12479210	**1.17 [1.12-1.22]**	**2.39E-13**	1.18 [1.15-1.22]	**1.14E-27**
2	242,698,640	rs34290285	*D2HGDH*	G	A	A		25.74%	rs34290285	**0.86 [0.82-0.91]**	**3.36E-09**	0.84 [0.81-0.87]	**6.71E-22**
5	110,401,872	rs1837253	*TSLP*	T	C	T		25.84%	rs1837253	**1.15 [1.09-1.20]**	**3.87E-08**	1.19 [1.15-1.23]	**2.70E-23**
5	110,467,499	rs1438673	*WDR36*	C	T	T	rs1837253	49.22%	rs1438673	**0.91 [0.87-0.94]**	**3.33E-06**	0.89 [0.86-0.92]	**7.65E-14**
5	131,799,626	rs3749833	*C5orf56*	T	C	C		26.08%	rs3749833	**1.10 [1.05-1.15]**	**7.37E-05**	1.13 [1.10-1.17]	**2.56E-13**
5	131,887,986	rs1986009	*RAD50*	C	A	A	rs3749833	18.71%	rs1986009	**1.14 [1.08-1.20]**	**8.27E-07**	1.16 [1.11-1.20]	**7.96E-14**
6	32,581,739	rs776111176	*HLA-DQA1*	A	AAT	A	rs9273410	14.85%	rs3997872 (0.82)	**0.83 [0.79-0.88]**	**1.21E-12**	0.83 [0.80-0.86]	**1.29E-19**
6	32,627,250	rs9273410	*HLA-DQB1*	C	A	C		44.70%	rs9273410	**1.15 [1.10-1.21]**	**1.35E-10**	1.21 [1.17-1.24]	**4.58E-32**
6	91,001,332	rs367983479	*BACH2*	CA	C	C		38.50%	rs1504215 (0.89)	**0.92 [0.88-0.96]**	**3.22E-04**	0.90 [0.88-0.93]	**2.35E-10**
8	81,266,924	rs71266076	*MIR5708*	C	CT	C		36.93%	rs13274067 (0.97)	**0.91 [0.87-0.95]**	**2.75E-05**	0.89 [0.87-0.92]	**1.38E-12**
9	6,208,030	rs144829310	*IL33*	G	T	T		16.40%	rs144829310	**1.20 [1.13-1.26]**	**9.15E-11**	1.21 [1.16-1.26]	**2.57E-21**
10	9,043,404	rs61840192	*LOC101928272*	G	A	A		42.70%	rs1775555 (0.98)	**0.86 [0.83-0.90]**	**7.24E-12**	0.86 [0.83-0.88]	**5.93E-24**
11	76,293,726	rs7936312	*C11orf30*	G	T	T		47.42%	rs7936312	**1.18 1.13-1.23]**	**9.98E-15**	1.16 [1.13-1.20]	**1.05E-22**
12	56,449,875	rs7305461	*RPS26*	A	C	A		44.61%	rs1131017 (0.98)	**0.94 [0.90-0.98]**	**4.96E-03**	0.91 [0.88-0.94]	**3.02E-09**
12	57,497,005	rs703816	*STAT6*	T	C	C		43.41%	rs703816	**1.07 [1.03-1.12]**	**9.57E-04**	1.12 [1.08-1.15]	**1.44E-12**
15	61,068,704	rs10519068	*RORA*	G	A	A		12.75%	rs10519068	**0.86 [0.80-0.92]**	**4.31E-06**	0.85 [0.82-0.89]	**9.72E-12**
15	67,441,750	rs72743461	*SMAD3*	C	A	A		23.60%	rs72743461	**1.09 [1.04-1.15]**	**2.34E-04**	1.13 [1.10-1.17]	**1.17E-12**
16	11,230,703	rs7203459	*CLEC16A*	T	C	C		24.56%	rs7203459	**0.91 [0.87-0.96]**	**3.22E-04**	0.86 [0.83-0.89]	**2.30E-16**
17	37,910,368	rs2941522	*IKZF3*	C	T	T		48.29%	rs2941522	**1.09 [1.04-1.13]**	**8.01E-05**	1.11 [1.08-1.14]	**1.30E-11**
17	47,439,302	rs112502960	*ZNF652*	G	A	A		35.92%	rs12952581 (0.98)	**1.08 [1.03-1.13]**	**5.16E-04**	1.11 [1.08-1.14]	**6.71E-11**

Supplementary Table 3: Summary of stage 1 results for variants previously associated with asthma susceptibility in GWAS (2007-2018)

Reported SNP	CHR	BP	Phenotype	Gene reported	Ancestry	#rsid	R2	noncoded	coded	coded freq.	INFO	OR [95% CI]	P	GWAS reported
rs662064	1	10,557,251	Asthma	*PEX14*	European	rs662064	1	T	C	67.82%	0.982	0.98 [0.93-1.02]	0.34173941	Pickrell 2016
rs4129267	1	154,426,264	Asthma	*IL6R*	European	Affx-5389767	1	C	T	40.86%	1	1.04 [1.00-1.08]	0.08688645	Ferreira 2011
*rs1102000	1	158,932,907	Asthma	*PYHIN1*	African American	ND	ND	ND	ND	ND	ND	ND	ND	Torgerson 2011
rs4233366	1	161,159,147	Asthma	*ADAMTS4*	European	rs4233366	1	C	T	26.48%	0.995	1.08 [1.03-1.14]	0.0012221	Pickrell 2016
rs1723018	1	167,433,420	Asthma	*CD247*	European	rs1723018	1	A	G	40.68%	0.997	0.88 [0.84-0.92]	**5.27E-09**	Pickrell 2016
rs6691738	1	173,152,036	Asthma	*TNFSF18*	European	rs6691738	1	å	G	30.05%	0.892	1.08 [1.03-1.14]	0.00131315	Pickrell 2016
rs2786098	1	197,325,908	Moderate-severe childhood asthma	*DENND1B*	European	Affx-6456370	1	T	G	77.97%	1	1.00 [0.95-1.05]	0.91947932	Sleiman 2010
rs6683383	1	203,100,504	Asthma	*ADORA1*	European	rs6683383	1	T	A	32.94%	0.991	0.97 [0.93-1.02]	0.23183104	Pickrell 2016
rs13412757	2	8,458,080	Asthma	*ID2*	European	rs13412757	1	G	A	33.85%	0.982	0.91 [0.87-0.96]	**9.92E-05**	Pickrell 2016
rs1558641	2	102,765,865	Childhood asthma + exacerbations	*IL1R1*	European	rs1558641	1	G	A	15.86%	0.972	0.89 [0.84-0.94]	**7.39E-05**	Bonnelykke 2014
rs202011557	2	102,913,642	Asthma	*IL1RL1*	European	rs202011557:102913642:A:AAAAC	1	A	AAAAC	13.18%	0.96	0.83 [0.78-0.89]	**3.38E-08**	Pickrell 2016
rs13408661	2	102,955,082	Asthma	*IL1RL1/IL18R1*	European	rs13408661	1	G	A	13.67%	0.993	0.84 [0.79-0.90]	**7.06E-08**	Ramasamy 2012
rs10173081	2	102,957,348	Asthma	*IL1RL1*	European	rs10173081	1	C	T	13.79%	0.999	0.84 [0.79-0.90]	**6.61E-08**	Torgerson 2011
rs1420101	2	102,957,716	Asthnma	*IL1RL1*	European/Multiancestry	Affx-17140840	1	C	T	38.37%	1	1.19 [1.14-1.25]	**3.21E-15**	Demenais 2018
rs1420101	2	102,957,716	Asthma	*IL1RL1*	European	Affx-17140840	1	C	T	38.37%	1	1.19 [1.14-1.25]	**3.07E-15**	Gudbjartsson 2009
rs10197862	2	102,966,549	Asthma + hay fever	*IL1RL1*	European	rs10197862	1	A	G	13.76%	0.996	0.84 [0.79-0.89]	**5.83E-08**	Ferreira 2014
rs3771166	2	102,986,222	Asthma	*IL1RL1/IL18R1*	European	rs3771166	1	G	A	38.10%	0.993	0.88 [0.82-0.90]	**2.54E-11**	Moffatt 2010
rs34290285	2	242,698,640	Asthma	*D2HGDH*	European	rs34290285	1	G	A	25.74%	0.995	0.82 [0.79-0.87]	**1.41E-14**	Pickrell 2016
rs73196739	3	188,402,471	Asthma	*LPP*	European	Affx-21876745	1	C	T	16.59%	1	0.94 [0.88-0.99]	0.02294229	Pickrell 2016
rs5743618	4	38,798,648	Asthma	*TLR1*	European	rs5743618:38798648:C:A	1	C	A	22.87%	0.988	0.93 [0.88-0.98]	0.00461438	Pickrell 2016
rs4833095	4	38,799,710	Asthma + hay fever	*TLR1*	European	Affx-24449268	1	T	C	20.13%	1	0.92 [0.87-0.97]	0.00209226	Ferreira 2014
rs7686660	4	144,003,159	Asthma	*USP38*	Japanese	Affx-23554961	1	T	G	25.60%	1	0.94 [0.90-0.99]	0.0139806	Hirota 2011
rs1588265	5	59,369,794	Mild-moderate childhood asthma with BHR	*PDE4D*	European	Affx-26773647	1	A	G	31.76%	1	0.97 [0.92-1.01]	0.14276726	Himes 2009
rs1837253	5	110,401,872	Asthma	*TSLP*	European	Affx-25366072	1	T	C	74.16%	1	1.24 [1.18-1.30]	**8.49E-18**	Torgerson 2011
rs1837253	5	110,401,872	Asthma	*TSLP*	Japanese	Affx-25366072	1	T	C	74.16%	1	1.24 [1.18-1.30]	**8.49E-18**	Hirota 2011
rs1837253	5	110,401,872	Asthma + hay fever	*TSLP*	European	Affx-25366072	1	T	C	74.16%	1	1.24 [1.18-1.30]	**8.49E-18**	Ferreira 2014
rs1837253	5	110,401,872	Asthma	*TSLP*	European	Affx-25366072	1	T	C	74.16%	1	1.24 [1.18-1.30]	**8.49E-18**	Pickrell 2016
rs10455025	5	110,404,999	Asthma	*SLC225A46, TSLP*	European/Multiancestry	rs10455025	1	A	C	35.74%	0.9959	1.19 [1.14-1.24]	**3.74E-14**	Demenais 2018
rs2416257	5	110,435,490	Asthma	*WDR36*	European	Affx-25366478	1	C	T	13.89%	1	0.89 [0.84-0.95]	**0.0005117**	Gudbjartsson 2009
rs1438673	5	110,467,499	Asthma + hay fever	*WDR36*	European	rs1438673	1	C	T	49.22%	0.969	0.84 [0.81-0.88]	**2.11E-14**	Ferreira 2014
rs2244012	5	131,901,225	Asthma	*RAD50*	European	Affx-25638647	1	A	G	19.46%	1	1.13 [1.07-1.19]	**7.53E-06**	Pickrell 2016
rs6871536	5	131,969,874	Childhood asthma + exacerbations	*RAD50*	European	rs6871536	1	T	C	19.17%	0.995	1.13 [1.07-1.20]	**7.11E-06**	Bonnelykke 2014
rs20541	5	131,995,964	Asthma	*IL13, RAD50, IL4*	European/Multiancestry	Affx-25639687	1	A	G	81.50%	1	0.85 [0.80-0.88]	**8.47E-09**	Demenais 2018
rs7705042	5	141,492,419	Asthma	*NDFIP1, GNDPA1, SPRY4*	Multiancestry	rs7705042	1	C	A	61.85%	1	1.08 [1.04-1.13]	**4.43E-04**	Demenais 2018
*rs200634877	5	141,529,761	Asthma	*NDFIP1*	European	ND	ND	ND	ND	ND	ND	ND	ND	Pickrell 2016
rs1233578	6	28,712,247	Asthma	*GPX5, TRIM27*	European	rs1233578	1	A	G	17.51%	0.9994	1.04 [0.98-1.10]	1.76E-01	Demenais 2018
rs2428494	6	31,322,197	Asthma	*HLAC*	European	rs2428494:31322197:T:A	1	T	A	47.91%	0.997	1.15 [1.10-1.20]	**5.04E-10**	Pickrell 2016
rs404860	6	32,184,345	Asthma	*NOTCH4*	Japanese	Affx-28468816	1	T	C	17.77%	1	1.00 [0.95-1.06]	0.89916188	Hirota 2011
rs2855812	6	31472720	Asthma	*MICB, HCP5, MCCD1*	European/Multiancestry	Affx-28458189	1	G	T	26.06%	1	1.09 [1.04-1.15]	**3.72E-04**	Demenais 2018
rs3104367	6	32,603,487	Asthma	*HLADRB5*	European	rs3104367:32603487:T:C	1	T	C	62.08%	0.956	1.22 [1.17-1.28]	**1.47E-18**	Pickrell 2016
rs9272346	6	32,604,372	Asthma	*HLADRB1, HLADQA1*	European/Multiancestry	rs9272346	1	G	A	61.45%	0.9462	1.22 [1.16-1.27]	**1.17E-17**	Demenais 2018
rs9273373	6	32,626,601	Asthma + hay fever	*HLA-DQB1*	European	rs9273373:32626601:A:G	1	A	G	60.62%	0.995	1.24 [1.18-1.29]	**3.12E-21**	Ferreira 2014

Reported SNP	CHR	BP	Phenotype	Gene reported	Ancestry	#rsid	R2	noncoded	coded	coded freq.	INFO	OR [95% CI]	P	GWAS reported
rs987870	6	33,042,880	Childhood onset asthma	*HLA-DPA1*	Japanese	Affx-28513044	1	A	G	14.48%	1	1.03 [0.97-1.09]	0.42324422	Noguchi 2011
rs1776883	6	34,156,444	Asthma	*GRM4*	European	rs1776883	1	C	T	53.25%	0.973	0.98 [0.94-1.02]	0.32358767	Almoguera 2016
rs58521088	6	90,985,198	Asthma	*BACH2*	European	rs58521088	1	A	T	35.54%	0.997	0.88 [0.85-0.93]	**1.06E-07**	Pickrell 2016
rs2325291	6	90,986,686	Asthma	*BACH2, GJA10, MAP3K7*	European/Multiancestry	rs2325291	1	G	A	35.53%	0.9974	0.89 [0.85-0.93]	**1.32E-07**	Demenais 2018
rs6967330	7	105,658,451	Childhood asthma + exacerbations	*CDHR3*	European	Affx-29418884	1	G	A	16.64%	1	1.04 [0.98-1.10]	0.20843707	Bonnelykke 2014
rs6959584	7	105,676,505	Asthma	*CDHR3*	European	rs6959584	1	C	T	12.89%	0.989	1.06 [1.00-1.13]	0.06402029	Pickrell 2016
rs3019885	8	18,025,645	Childhood onset asthma	*SLC30A8*	Japanese	rs2047963	0.996	T	C	45.37%	0.999	0.97 [0.93-1.01]	0.10761244	Noguchi 2011
rs12543811	8	81,278,885	Asthma	*TPD52, ZBTB10*	European/Multiancestry	rs12543811	1	G	A	64.95%	0.9925	0.88 [0.84-0.92]	**5.39E-08**	Demenais 2018
rs10957978	8	81,285,139	Asthma	*ZBTB10*	European	rs10957978	1	G	T	64.49%	0.984	0.89 [0.85-0.93]	**2.46E-07**	Pickrell 2016
rs7009110	8	81,291,879	Asthma + hay fever	*ZBTB10*	European	rs7009110	1	T	C	61.65%	0.994	0.88 [0.85-0.92]	**6.92E-08**	Ferreira 2014
rs72699186	9	6,175,855	Asthma + hay fever	*IL33*	European	rs72699186	1	A	T	16.44%	0.991	1.23 [1.16-1.30]	**8.38E-12**	Ferreira 2014
rs1342326	9	6,190,076	Asthma	*IL33*	European	Affx-33938382	1	A	C	16.62%	1	1.22 [1.15-1.29]	**2.06E-11**	Moffatt 2010
rs2381416	9	6,193,455	Asthma	*IL33*	European	Affx-33938420	1	C	A	73.63%	1	0.85 [0.81-0.89]	**1.60E-11**	Torgerson 2011
rs144829310	9	6,208,030	Asthma	*IL33*	European	rs144829310	1	G	T	16.40%	0.991	1.23 [1.16-1.30]	**3.68E-12**	Pickrell 2016
rs992969	9	6,209,697	Asthma	*RANBP6, IL33*	European/Multiancestry	rs992969	1	A	G	74.68%	0.9901	0.84 [0.80-0.88]	**5.95E-12**	Demenais 2018
rs928413	9	6,213,387	Childhood asthma + exacerbations	*IL33*	European	rs928413	1	G	A	74.09%	0.984	0.84 [0.80-0.89]	**1.53E-11**	Bonnelykke 2014
rs72721168	9	27,308,288	Asthma	*EQTN*	European	rs72721168	1	A	C	3.71%	0.991	1.05 [0.94-1.18]	0.37310685	Almoguera 2016
rs10508372	10	8,972,018	Asthma	*GATA3*	Japanese	Affx-3813772	1	G	A	5.17%	1	0.95 [0.85-1.03]	0.18978182	Hirota 2011
rs2589561	10	9,046,645	Asthma	*GATA3, CELF2*	European/Multiancestry	rs2589561	1	A	G	80.26%	0.9176	0.87 [0.82-0.92]	**7.32E-07**	Demenais 2018
rs12413578	10	9,049,253	Asthma	*GATA3*	European	Affx-3823389	1	C	T	10.42%	1	0.77 [0.73-0.84]	**2.38E-11**	Pickrell 2016
rs7130588	11	76,270,683	Asthma	*LRRC32*	European	Affx-5959929	1	A	G	35.95%	1	1.12 [1.07-1.17]	**6.68E-07**	Ferreira 2011
rs7936323	11	76,293,758	Asthma	*C11orf30*	European	rs7936323	1	G	A	47.45%	0.998	1.14 [1.10-1.19]	**1.22E-09**	Pickrell 2016
rs2155219	11	76,299,194	Asthma + hay fever	*LRRC32*	European	Affx-5960597	1	G	T	50.01%	1	1.13 [1.08-1.18]	**4.40E-08**	Ferreira 2014
rs7927894	11	76,301,316	Asthma	*EMSY, LRRC32*	European/Multiancestry	Affx-5960642	1	C	T	38.94%	1	1.14 [1.09-1.19]	**1.51E-08**	Demenais 2018
rs1701704	12	56,412,487	Asthma	*IKZF4*	Japanese	Affx-8212375	1	T	G	34.24%	1	1.11 [1.06-1.16]	**1.16E-05**	Hirota 2011
rs167769	12	57,503,775	Asthma	*STAT6, NAB2, LRP1*	Multiancestry	Affx-8225554	1	C	T	40.13%	1	1.15 [1.11-1.21]	**1.58E-10**	Demenais 2018
rs3001426	12	57,509,055	Asthma	*STAT6*	European	rs3001426	1	T	C	45.67%	0.984	1.14 [1.09-1.19]	**3.94E-09**	Pickrell 2016
rs3784099	14	68,749,927	Asthma	*RAD51B*	European	Affx-10832504	1	G	A	27.81%	1	1.06 [1.01-1.11]	0.01229815	Pickrell 2016
rs10519068	15	61,068,704	Asthma	*RORA*	European	rs10519068	1	G	A	12.75%	0.997	0.85 [0.80-0.90]	**4.81E-07**	Pickrell 2016
rs11071558	15	61,069,421	Asthma	*RORA, NARG2, VPS13C*	European/Multiancestry	rs11071558	1	A	G	12.97%	0.9967	0.86 [0.81-0.91]	**1.61E-06**	Demenais 2018
rs11071559	15	61,069,988	Asthma	*RORA*	European	Affx-11799439	1	C	T	12.78%	1	0.85 [0.80-0.91]	**1.61E-06**	Moffatt 2010
rs744910	15	67,446,785	Asthma	*SMAD3*	European	Affx-11880511	1	G	A	51.41%	1	0.90 [0.86-0.94]	**2.05E-06**	Moffatt 2010
rs56375023	15	67,448,363	Asthma	*SMAD3*	European	rs56375023	1	G	A	23.57%	0.997	1.18 [1.12-1.24]	**1.32E-10**	Pickrell 2016
rs2033784	15	67,449,660	Asthma	*SMAD3, SMAD6*	European/Multiancestry	Affx-11880551	1	A	G	29.79%	1	1.16 [1.11-1.21]	**6.43E-10**	Demenais 2018
rs17294280	15	67,468,285	Asthma + hay fever	*SMAD3*	European	rs17294280	1	A	G	25.59%	0.903	1.15 [1.09-1.21]	**2.04E-07**	Ferreira 2014
rs17806299	16	11,199,980	Asthma	*CLEC16A, DEXI, SOCS1*	European/Multiancestry	rs17806299	1	G	A	19.92%	0.9988	0.85 [0.80-0.89]	**8.57E-10**	Demenais 2018
rs62026376	16	11,228,712	Asthma + hay fever	*CLEC16A*	European	rs62026376	1	C	T	25.11%	0.997	0.82 [0.78-0.86]	**1.34E-14**	Ferreira 2014
rs7203459	16	11,230,703	Asthma	*CLEC16A*	European	rs7203459	1	T	C	24.56%	0.996	0.81 [0.78-0.86]	**7.83E-16**	Pickrell 2016
rs2952156	17	37,876,835	Asthma	*ERBB2, PGAP3, MIEN1*	European/Multiancestry	rs2952156	1	A	G	69.08%	0.9973	0.90 [0.86-0.94]	**1.30E-05**	Demenais 2018
rs11655198	17	38,026,169	Asthma	*ZPBP2*	European	rs11655198	1	C	T	51.31%	1	0.90 [0.86-0.94]	**9.70E-07**	Pickrell 2016
rs2305480	17	38,062,196	Asthma	*GSDMB*	European	Affx-13850821	1	G	A	46.65%	1	0.91 [0.87-0.95]	**2.89E-05**	Moffatt 2010
rs2305480	17	38,062,196	Childhood asthma + exacerbations	*GSDMB*	European	Affx-13850821	1	G	A	46.65%	1	0.91 [0.87-0.95]	**2.89E-05**	Bonnelykke 2014
rs11078927	17	38,064,405	Asthma	*GSDMB*	European	rs11078927	1	C	T	46.63%	1	0.91 [0.87-0.95]	**2.99E-05**	Torgerson 2011
rs7216389	17	38,069,949	Childhood asthma	*ORMDL3*	European	Affx-13850945	1	C	T	48.44%	1	1.11 [1.07-1.16]	**1.58E-06**	Moffatt 2007

Reported SNP	CHR	BP	Phenotype	Gene reported	Ancestry	#rsid	R2	noncoded	coded	coded freq.	INFO	OR [95% CI]	P	GWAS reported
rs4794820	17	38,089,344	Moderate-severe asthma	*ORMDL3/GSDMB*	European	rs4794820	1	A	G	54.97%	0.984	1.08 [1.03-1.13]	**0.00052197**	Wan 2012
rs3894194	17	38,121,993	Asthma	*GSDMA*	European	Affx-13851698	1	G	A	45.10%	1	1.06 [1.02-1.11]	0.00459175	Moffatt 2010
rs7212938	17	38,122,680	Asthma + hay fever	*GSDMA*	European	Affx-13851705	1	G	T	50.30%	1	0.95 [0.91-0.99]	0.01268569	Ferreira 2014
rs17637472	17	47,461,433	Asthma	*ZNF652, PHB*	European/Multiancestry	rs17637472	1	G	A	39.79%	0.9556	1.14 [1.09-1.19]	**2.78E-08**	Demenais 2018
rs2284033	22	37,534,034	Asthma	*IL2RB*	European	Affx-19547973	1	G	A	43.45%	1	0.95 [0.91-0.99]	0.0141752	Moffatt 2010

- Studies focused to reported GWAS for asthma with >500 cases and controls and SNPs meeting genome wide significance (P<5x10-8)
- Sentinel SNP reported in each study was analysed.
- Gene reported are those commented on in the original manuscript.
- *SNPs not evaluated (not determined)
- 80 SNPs tested overall (Bonferroni Correction P<0.0006), SNPs meeting criteria shown in bold (60 individual SNPs).
- Of 24 SNPs that did not meet correction, 10/24 showed nominal association (P<0.05).

Supplementary Table 4: Summary of stage 1 results for variants previously associated with allergic diseases in the Study of Ferreira et. al 2017. 87 signals with P<0.05 for moderate-severe asthma have their P value in bold; 40 signals had P<3.68×10^{-4} for moderate-severe asthma (Bonferroni correction for 136 tests).

				**Allergic diseases (Ferreira et al. 2017)**			**Moderate-severe asthma (this study)**		
**Genecontext**	**rsid**	**Position (b37)**	**Effect/ other allele**	**Effect allele frequency**	**OR (95% CI)**	**P-value**	**Effect allele frequency**	**OR (95% CI)**	**P**
TNFRSF14-[]-FAM213B	rs10910095	1:2510755	G/A	84%	1.04 (1.03, 1.06)	2.70E-08	86%	1.04 (0.9, 1.11)	1.62E-01
[RERE]	rs301806	1:8482078	T/C	54%	1.05 (1.04, 1.06)	1.80E-20	58%	1.12 (1.07, 1.17)	**3.47E-07**
[RUNX3]	rs760805	1:25251923	T/A	58%	1.04 (1.03, 1.05)	6.40E-13	57%	1.07 (1.03, 1.12)	**1.06E-03**
SFPQ-[]-ZMYM4	rs76167968	1:35681738	T/C	93%	1.06 (1.04, 1.08)	1.30E-08	92%	1.01 (0.92, 1.09)	8.67E-01
C1orf54-[]-MRPS21	rs7512552	1:150265704	C/T	52%	1.03 (1.02, 1.04)	1.40E-09	51%	1.04 (0.99, 1.08)	1.14E-01
[FLG]	rs61816761	1:152285861	A/G	1%	1.22 (1.17, 1.28)	7.40E-21	2%	1.5 (1.28, 1.75)	**3.04E-07**
RPTN-[]-HRNR	rs12123821	1:152179152	T/C	5%	1.11 (1.08, 1.14)	6.80E-17	5%	1.25 (1.13, 1.38)	**9.67E-06**
[RORC]	rs11204896	1:151796742	C/G	90%	1.06 (1.05, 1.08)	2.40E-12	90%	1.02 (0.92, 1.09)	6.43E-01
[IL6R]	rs2228145	1:154426970	C/A	35%	1.04 (1.03, 1.05)	4.30E-13	41%	1.04 (1, 1.09)	7.00E-02
NDUFS2-[]-FCER1G	rs2070901	1:161185058	T/G	26%	1.04 (1.03, 1.05)	1.30E-11	27%	1.08 (1.03, 1.14)	**1.11E-03**
[CD247]	rs2988277	1:167431352	C/T	66%	1.04 (1.03, 1.05)	4.00E-14	60%	1.14 (0.84, 1.19)	**1.08E-08**
TNFSF18--[]-TNFSF4	rs4090390	1:173146921	A/C	22%	1.05 (1.04, 1.06)	1.30E-15	24%	1.09 (1.04, 1.15)	**6.48E-04**
FASLG-[]--TNFSF18	rs1102705	1:172700868	G/A	11%	1.06 (1.04, 1.08)	3.10E-10	11%	1.06 (0.87, 1.14)	9.89E-02
[ITPKB]	rs697852	1:226914734	A/G	85%	1.04 (1.03, 1.05)	1.60E-09	82%	1.09 (1.03, 1.15)	**2.43E-03**
[LINC00299]	rs10174949	2:8442248	G/A	68%	1.07 (1.05, 1.08)	7.30E-31	71%	1.11 (0.86, 1.16)	**1.55E-05**
[LOC339807]	rs4671601	2:64836267	C/T	83%	1.04 (1.03, 1.05)	8.80E-09	80%	1.04 (0.91, 1.1)	1.56E-01
[IL18R1]	rs10865050	2:102941311	G/A	85%	1.13 (1.11, 1.15)	7.00E-61	86%	1.19 (0.79, 1.26)	**3.04E-08**
IL1RL2-[]-IL18R1	rs12470864	2:102926362	A/G	35%	1.06 (1.05, 1.07)	4.20E-26	39%	1.2 (1.15, 1.25)	**5.06E-16**
BCL2L11--[]--ANAPC1	rs4848612	2:112388538	A/G	76%	1.04 (1.03, 1.05)	2.30E-10	77%	1.03 (0.98, 1.09)	2.18E-01
BCL2L11--[]--ANAPC1	rs13403656	2:112269127	A/T	16%	1.05 (1.03, 1.06)	2.20E-08	13%	1.04 (0.9, 1.11)	1.85E-01
[IL1B]	rs1143633	2:113590467	C/T	70%	1.03 (1.02, 1.04)	1.70E-10	64%	1.03 (0.93, 1.08)	1.92E-01
KYNU-[]-ARHGAP15	rs74847330	2:143831599	A/G	88%	1.05 (1.03, 1.06)	1.80E-09	87%	1.05 (0.9, 1.11)	1.59E-01
[PLCL1]	rs1064213	2:198950240	G/A	47%	1.04 (1.03, 1.04)	5.40E-12	52%	1.02 (0.94, 1.06)	3.74E-01
CCL20-[]-DAW1	rs13384448	2:228707862	T/C	76%	1.04 (1.03, 1.05)	2.80E-12	76%	1.08 (1.02, 1.13)	**3.61E-03**
[INPP5D]	rs1057258	2:234115629	C/T	80%	1.05 (1.03, 1.06)	1.40E-10	82%	1.06 (0.89, 1.12)	**4.58E-02**
[D2HGDH]	rs34290285	2:242698640	G/A	73%	1.08 (1.07, 1.1)	4.00E-33	74%	1.21 (0.79, 1.27)	**5.79E-15**
[GLB1]	rs6776757	3:33069091	G/A	47%	1.03 (1.02, 1.04)	3.10E-10	51%	1.08 (0.89, 1.12)	**9.13E-04**
LINC00870--[]-RYBP	rs61192126	3:72394852	T/C	69%	1.04 (1.03, 1.05)	8.90E-11	71%	1.02 (0.94, 1.06)	5.36E-01
FAM172BP-[]-TRMT10C	rs13088318	3:101242751	A/G	62%	1.03 (1.02, 1.04)	8.60E-09	66%	1.05 (0.91, 1.1)	**3.36E-02**
[SLC15A2]	rs75557865	3:121652141	G/A	57%	1.03 (1.02, 1.04)	1.60E-08	52%	1.05 (0.92, 1.09)	**4.24E-02**
[RASA2]	rs10663129	3:141321836	ACT/A	33%	1.04 (1.03, 1.05)	1.10E-13	33%	1.06 (1.01, 1.11)	**1.36E-02**
[LPP]	rs60946162	3:188133336	T/C	42%	1.04 (1.03, 1.05)	8.60E-15	45%	1.05 (1.01, 1.1)	**1.83E-02**
[LPP]	rs17607589	3:188402586	C/T	84%	1.05 (1.04, 1.07)	1.80E-14	83%	1.07 (0.88, 1.14)	**1.57E-02**
BCL6--[]--LPP-AS2	rs519973	3:187633268	A/G	35%	1.03 (1.02, 1.04)	4.50E-10	34%	1.04 (1, 1.09)	8.13E-02
BCL6--[]-LPP-AS2	rs2030030	3:187793833	T/C	86%	1.04 (1.03, 1.05)	1.00E-08	84%	1.08 (0.88, 1.14)	**1.46E-02**

Genecontext	rsid	Position (b37)	Effect/ other allele	Allergic diseases (Ferreira et al. 2017) Effect allele frequency	OR (95% CI)	P-value	Moderate-severe asthma (this study) Effect allele frequency	OR (95% CI)	P
FBXO45-[]-CEP19	rs80064395	3:196372546	C/T	94%	1.07 (1.05, 1.09)	1.60E-12	91%	1.14 (0.81, 1.23)	**6.48E-04**
STX18--[]-MSX1	rs10033073	4:4775401	G/A	35%	1.04 (1.03, 1.05)	1.20E-10	37%	1.01 (0.96, 1.05)	7.71E-01
[TLR1]	rs5743618	4:38798648	C/A	70%	1.1 (1.09, 1.11)	3.30E-58	77%	1.08 (0.88, 1.13)	**4.01E-03**
[MANBA]	rs227275	4:103593898	C/A	56%	1.03 (1.02, 1.04)	3.70E-11	53%	0.99 (0.95, 1.04)	7.71E-01
[ADAD1]	rs4145717	4:123316076	T/G	34%	1.06 (1.05, 1.07)	9.20E-27	33%	1.05 (1, 1.1)	**4.10E-02**
IL2-[]-IL21	rs150254607	4:123454110	ATAT/A	7%	1.08 (1.06, 1.1)	4.60E-14	10%	1.15 (1.07, 1.24)	**1.62E-04**
[FAM105A]	rs16903574	5:14610309	G/C	8%	1.07 (1.05, 1.09)	1.40E-12	49%	1.04 (0.99, 1.09)	8.83E-02
[IL7R]	rs7717955	5:35862841	C/T	71%	1.07 (1.06, 1.08)	9.10E-36	72%	1.1 (0.87, 1.15)	**8.58E-05**
DAB2---[]--PTGER4	rs7714574	5:40492655	T/C	53%	1.03 (1.02, 1.04)	5.90E-10	60%	1.04 (0.99, 1.08)	9.74E-02
WDR36-[]-CAMK4	rs6594499	5:110470137	C/A	48%	1.08 (1.06, 1.09)	4.60E-46	50%	1.18 (0.81, 1.23)	**1.33E-14**
SLC25A46-[]--TSLP	rs6869502	5:110166083	T/A	18%	1.08 (1.07, 1.09)	6.40E-29	16%	1.12 (1.05, 1.18)	**2.42E-04**
SLC25A46--[]-TSLP	rs1837253	5:110401872	C/T	78%	1.07 (1.06, 1.08)	1.60E-31	74%	1.24 (1.18, 1.3)	**2.81E-18**
SLC25A46-[]--TSLP	rs1814576	5:110159879	C/T	9%	1.12 (1.1, 1.15)	1.40E-22	4%	1.05 (0.93, 1.18)	4.42E-01
[TNFAIP8]	rs250308	5:118684297	T/C	37%	1.03 (1.02, 1.04)	4.00E-09	39%	1.05 (0.91, 1.1)	**3.36E-02**
[IL13]	rs848	5:131996500	A/C	24%	1.07 (1.05, 1.08)	1.50E-24	18%	1.17 (0.81, 1.24)	**1.15E-08**
[C5orf56]	rs3749833	5:131799626	C/T	30%	1.04 (1.03, 1.05)	3.30E-11	26%	1.18 (1.12, 1.23)	**5.43E-11**
RAD50-[]-IL13	rs3091307	5:131989136	G/A	20%	1.06 (1.05, 1.07)	3.60E-21	19%	1.15 (1.09, 1.21)	**7.85E-07**
[NDFIP1]	rs10068717	5:141494934	T/C	62%	1.04 (1.03, 1.05)	4.80E-15	62%	1.08 (1.04, 1.13)	**3.36E-04**
[DIAPH1]	rs740474	5:140925362	C/T	42%	1.03 (1.02, 1.04)	5.60E-11	39%	1.05 (0.91, 1.1)	**2.34E-02**
MIR3142-[]-MIR146A	rs2910162	5:159909345	G/A	68%	1.03 (1.02, 1.04)	2.50E-09	57%	1.03 (0.98, 1.07)	2.76E-01
LMAN2-[]-RGS14	rs13153019	5:176782218	C/T	26%	1.04 (1.02, 1.05)	1.30E-08	25%	1.03 (0.98, 1.08)	2.25E-01
HLA-DQA1-[]-HLA-DQB1	rs34004019	6:32626403	A/G	70%	1.1 (1.09, 1.11)	3.80E-52	79%	1.21 (0.78, 1.27)	**6.28E-13**
[HLA-B]	rs2854001	6:31323012	A/G	18%	1.06 (1.05, 1.07)	1.20E-19	23%	1.08 (1.02, 1.13)	**3.81E-03**
HLA-B-[]-MICA	rs2507978	6:31351664	G/A	41%	1.04 (1.02, 1.05)	1.20E-10	44%	0.98 (0.98, 1.03)	4.11E-01
[ITPR3]	rs10947428	6:33647058	C/T	19%	1.05 (1.03, 1.06)	3.50E-13	21%	1.01 (0.96, 1.07)	6.17E-01
[HLA-J]	rs9259819	6:29893575	G/T	50%	1.04 (1.02, 1.05)	2.40E-09	52%	0.97 (0.99, 1.01)	1.71E-01
NCR3-[]-AIF1	rs28895016	6:31574525	C/T	91%	1.1 (1.07, 1.12)	9.40E-16	94%	1.07 (0.86, 1.17)	1.24E-01
[HLA-DPA1]	rs3097670	6:33046752	G/C	88%	1.06 (1.05, 1.08)	7.70E-12	88%	1.02 (0.92, 1.09)	5.92E-01
[BACH2]	rs2134814	6:90987512	C/G	65%	1.05 (1.04, 1.06)	1.70E-17	64%	1.13 (0.85, 1.18)	**6.82E-08**
[ATG5]	rs9372120	6:106667535	G/T	18%	1.04 (1.03, 1.05)	4.20E-11	21%	1.07 (1.02, 1.13)	**7.24E-03**
[PTPRK]	rs35469349	6:128294709	A/T	26%	1.04 (1.03, 1.05)	2.30E-10	31%	1.1 (1.05, 1.15)	**1.30E-04**
[TNFAIP3]	rs5029937	6:138195151	G/T	97%	1.08 (1.05, 1.11)	2.40E-08	97%	1.27 (0.7, 1.43)	**5.32E-05**
[ARID1B]	rs9383820	6:157419508	C/T	76%	1.04 (1.02, 1.05)	1.20E-08	79%	1.03 (0.92, 1.09)	2.68E-01
RNASET2-[]-MIR3939	rs72033857	6:167390671	C/CCTTT	11%	1.06 (1.04, 1.08)	1.20E-09	10%	1.03 (0.96, 1.11)	3.59E-01
ITGB8--[]-ABCB5	rs6461503	7:20560996	T/C	53%	1.04 (1.03, 1.05)	1.70E-14	48%	1.07 (0.9, 1.11)	**3.68E-03**
[ITGB8]	rs10486391	7:20376018	A/G	53%	1.03 (1.02, 1.04)	6.80E-09	59%	1.05 (0.91, 1.09)	**4.11E-02**
[JAZF1]	rs6977955	7:28156887	T/C	21%	1.05 (1.03, 1.06)	7.10E-13	20%	1.06 (1, 1.11)	**4.40E-02**
C7orf72-[]-IKZF1	rs17664743	7:50253897	A/G	20%	1.04 (1.03, 1.05)	6.20E-11	21%	1.02 (0.97, 1.08)	4.00E-01
[GSAP]	rs4296977	7:77018542	C/T	16%	1.06 (1.04, 1.07)	2.10E-13	14%	1.07 (0.88, 1.14)	**2.87E-02**

Genecontext	rsid	Position (b37)	Effect/ other allele	Allergic diseases (Ferreira et al. 2017)			Moderate-severe asthma (this study)		
				Effect allele frequency	OR (95% CI)	P-value	Effect allele frequency	OR (95% CI)	P
MIR5708--[]--ZBTB10	rs7824394	8:81292599	A/C	37%	1.05 (1.04, 1.06)	3.50E-20	36%	1.13 (0.85, 1.18)	**1.72E-07**
[MYC]	rs6990534	8:128814091	A/G	36%	1.04 (1.03, 1.05)	6.40E-14	29%	1.07 (0.89, 1.12)	**6.30E-03**
RANBP6--[]-IL33	rs144829310	9:6208030	T/G	16%	1.09 (1.08, 1.1)	1.20E-35	16%	1.23 (1.16, 1.3)	**1.91E-12**
RANBP6-[]--IL33	rs343478	9:6051399	G/A	52%	1.03 (1.02, 1.04)	2.60E-10	55%	1.07 (0.9, 1.12)	**3.48E-03**
[JAK2]	rs16922576	9:5064193	C/T	32%	1.04 (1.02, 1.05)	3.20E-10	28%	1.06 (1.01, 1.11)	**1.51E-02**
PHF19-[]-TRAF1	rs10760123	9:123650534	T/G	38%	1.03 (1.02, 1.04)	5.20E-09	35%	1.01 (0.95, 1.05)	7.41E-01
C9orf114-[]-LRRC8A	rs12551834	9:131613191	G/A	92%	1.06 (1.04, 1.08)	3.00E-09	90%	1.08 (0.86, 1.16)	**3.14E-02**
[IL2RA]	rs61839660	10:6094697	T/C	7%	1.08 (1.06, 1.1)	4.40E-19	10%	1.07 (1, 1.15)	5.39E-02
[IL2RA]	rs4747846	10:6074451	C/G	52%	1.04 (1.03, 1.05)	1.00E-11	50%	1.09 (1.04, 1.14)	**1.30E-04**
GATA3---[]---SFTA1P	rs12413578	10:9049253	C/T	90%	1.1 (1.08, 1.11)	1.30E-27	90%	1.27 (0.73, 1.36)	**1.20E-11**
GATA3---[]---SFTA1P	rs1444789	10:9064361	C/T	20%	1.07 (1.05, 1.08)	1.50E-22	21%	1.16 (1.1, 1.23)	**1.81E-07**
GATA3--[]SFTA1P	rs11255753	10:8605553	T/G	26%	1.04 (1.03, 1.05)	2.00E-12	27%	1.09 (1.03, 1.14)	**7.94E-04**
GATA3---[]---SFTA1P	rs72782676	10:9032555	C/G	98%	1.3 (1.21, 1.41)	3.20E-11	99%	1.73 (0.44, 2.28)	**7.95E-05**
GATA3---[]---SFTA1P	rs2025758	10:8841669	T/C	58%	1.04 (1.03, 1.05)	4.70E-15	54%	1.09 (0.88, 1.13)	**1.92E-04**
GATA3---[]---SFTA1P	rs11255968	10:8936162	C/T	96%	1.09 (1.06, 1.12)	7.70E-09	97%	1.25 (0.7, 1.43)	**1.21E-03**
[ZNF365]	rs2893907	10:64382359	C/A	58%	1.03 (1.02, 1.04)	1.80E-09	56%	1.04 (0.99, 1.09)	1.02E-01
C10orf95-[]-ACTR1A	rs10883723	10:104225832	C/T	31%	1.03 (1.02, 1.04)	1.60E-08	35%	1.03 (0.98, 1.07)	2.28E-01
AP5B1-[]-OVOL1	rs479844	11:65551957	G/A	57%	1.04 (1.03, 1.05)	1.60E-13	56%	1.07 (1.02, 1.11)	**2.49E-03**
WNT11--[]-LRRC32	rs7936323	11:76293758	A/G	46%	1.09 (1.08, 1.1)	2.20E-63	47%	1.14 (1.1, 1.19)	**7.00E-10**
WNT11--[]-LRRC32	rs55646091	11:76299431	A/G	5%	1.18 (1.15, 1.21)	2.30E-40	5%	1.36 (1.23, 1.51)	**2.00E-09**
WNT11--[]-LRRC32	rs11236814	11:76343428	A/T	91%	1.07 (1.05, 1.09)	4.00E-14	90%	1.06 (0.88, 1.13)	1.41E-01
SESN3--[]-FAM76B	rs59593577	11:95425526	C/T	87%	1.05 (1.04, 1.07)	1.60E-11	87%	1.05 (0.89, 1.12)	9.97E-02
LAYN-[]-SIK2	rs7130753	11:111470567	C/T	69%	1.05 (1.03, 1.06)	7.00E-15	73%	1.07 (0.89, 1.13)	**4.64E-03**
DDX6-[]-CXCR5	rs12365699	11:118743286	G/A	85%	1.06 (1.05, 1.08)	5.10E-18	84%	1.05 (0.9, 1.12)	8.29E-02
KIRREL3-AS3---[]--ETS1	rs56129466	11:128158189	A/G	79%	1.05 (1.03, 1.06)	1.90E-13	78%	1.08 (0.88, 1.13)	**4.40E-03**
[HDAC7]	rs55726902	12:48196982	G/A	80%	1.05 (1.04, 1.06)	2.60E-16	76%	1.1 (0.87, 1.15)	**2.16E-04**
[AQP2]	rs11169225	12:50345671	A/T	18%	1.05 (1.03, 1.06)	1.20E-11	20%	1.09 (1.04, 1.15)	**8.44E-04**
[STAT6]	rs1059513	12:57489709	T/C	89%	1.08 (1.07, 1.1)	1.00E-22	89%	1.18 (0.79, 1.26)	**2.80E-06**
SUOX-[]-IKZF4	rs10876864	12:56401085	G/A	39%	1.05 (1.04, 1.06)	1.40E-19	43%	1.13 (0.85, 1.18)	**2.18E-08**
[ATXN2]	rs7137828	12:111932800	T/C	54%	1.03 (1.02, 1.04)	2.20E-10	51%	1.01 (0.97, 1.05)	7.42E-01
SPPL3-[]-HNF1A-AS1	rs6489785	12:121363724	T/C	37%	1.04 (1.03, 1.05)	1.60E-15	40%	1.06 (0.9, 1.11)	**9.06E-03**
C12orf65-[]-CDK2AP1	rs63406760	12:123742692	T/TG	77%	1.05 (1.03, 1.06)	3.00E-13	79%	1.03 (0.98, 1.09)	2.63E-01
[FOXO1]	rs4943794	13:41173408	C/G	23%	1.04 (1.03, 1.06)	7.20E-12	21%	1.06 (1.01, 1.12)	**2.30E-02**
PIBF1-[]-KLF5	rs9573092	13:73627275	A/G	70%	1.03 (1.02, 1.04)	2.70E-08	65%	1.03 (0.93, 1.08)	1.55E-01
[PSMA6]	rs1048990	14:35761675	G/C	16%	1.04 (1.03, 1.05)	1.00E-08	16%	1.07 (1.01, 1.13)	**3.10E-02**
FOXA1-[]--TTC6	rs111914382	14:38097001	TG/T	27%	1.04 (1.02, 1.05)	8.10E-09	24%	1.01 (0.96, 1.06)	6.50E-01
[RAD51B]	rs2104047	14:68754417	T/C	36%	1.04 (1.03, 1.05)	1.60E-13	28%	1.06 (0.9, 1.11)	**1.38E-02**
JDP2-[]-BATF	rs9323612	14:75968608	A/G	70%	1.03 (1.02, 1.04)	8.60E-09	67%	1.03 (0.93, 1.08)	1.73E-01
RCOR1-[]-TRAF3	rs9989163	14:103235012	A/G	49%	1.03 (1.02, 1.04)	1.90E-08	47%	1 (0.96, 1.05)	9.21E-01

Genecontext	rsid	Position (b37)	Effect/ other allele	Allergic diseases (Ferreira et al. 2017) Effect allele frequency	OR (95% CI)	P-value	Moderate-severe asthma (this study) Effect allele frequency	OR (95% CI)	P
RTF1-[]-ITPKA	rs12440045	15:41782684	C/A	55%	1.03 (1.02, 1.04)	4.90E-10	53%	1.07 (1.02, 1.11)	**4.36E-03**
[RORA]	rs10519067	15:61068347	G/A	86%	1.06 (1.04, 1.07)	9.30E-13	87%	1.18 (0.8, 1.26)	**3.91E-07**
[SMAD3]	rs56375023	15:67448363	A/G	21%	1.07 (1.06, 1.09)	8.20E-32	24%	1.18 (1.12, 1.24)	**7.18E-11**
[IQGAP1]	rs3540	15:91045408	G/A	65%	1.04 (1.03, 1.05)	3.30E-11	68%	1.01 (0.95, 1.06)	6.37E-01
CLEC16A-[]-RMI2	rs11644510	16:11277358	C/T	64%	1.07 (1.06, 1.08)	6.10E-38	63%	1.17 (0.82, 1.22)	**1.61E-11**
RMI2-[]--LITAF	rs12596613	16:11491007	C/G	67%	1.03 (1.02, 1.04)	6.10E-09	66%	0.98 (0.97, 1.03)	4.05E-01
SMTNL2-[]-ALOX15	rs71368508	17:4521473	C/A	99%	1.12 (1.08, 1.17)	2.00E-09	98%	1.24 (0.7, 1.43)	**4.58E-03**
[GSDMB]	rs921650	17:38069076	A/G	48%	1.06 (1.05, 1.07)	5.70E-30	48%	1.11 (1.06, 1.15)	**3.91E-06**
CCR7-[]-SMARCE1	rs112401631	17:38764524	A/T	1%	1.26 (1.21, 1.31)	2.20E-26	2%	1.26 (1.08, 1.46)	**3.29E-03**
CCR7-[]-SMARCE1	rs11464691	17:38770641	TA/T	57%	1.05 (1.04, 1.06)	1.40E-21	63%	1.06 (1.02, 1.11)	**5.81E-03**
[PSMD3]	rs11652139	17:38149033	A/G	59%	1.05 (1.04, 1.06)	7.50E-22	60%	1.06 (1.02, 1.11)	**8.86E-03**
[STAT5B]	rs7207591	17:40414862	A/G	76%	1.04 (1.03, 1.05)	1.40E-09	73%	1.06 (0.89, 1.12)	**1.21E-02**
MAP3K14-[]-ARHGAP27	rs7214661	17:43430696	G/A	32%	1.03 (1.02, 1.04)	1.20E-08	34%	1.03 (0.99, 1.08)	1.93E-01
[ZNF652]	rs9889262	17:47398070	A/T	35%	1.04 (1.03, 1.05)	9.70E-16	36%	1.13 (1.09, 1.19)	**2.07E-08**
DYNAP-[]--RAB27B	rs4801001	18:52336175	T/C	42%	1.03 (1.02, 1.04)	5.90E-09	41%	1.02 (0.94, 1.07)	3.40E-01
[TNFRSF11A]	rs4574025	18:60009814	T/C	56%	1.03 (1.02, 1.04)	6.80E-09	53%	1.01 (0.96, 1.05)	8.08E-01
SLC7A10-[]-CEBPA	rs10414065	19:33721455	C/T	92%	1.1 (1.08, 1.12)	6.10E-18	94%	1.19 (0.77, 1.3)	**1.94E-04**
[NFATC2]	rs3787184	20:50157837	A/G	78%	1.05 (1.04, 1.06)	1.10E-12	83%	1.02 (0.93, 1.08)	5.15E-01
[ZNF217]	rs2766678	20:52208356	G/A	21%	1.06 (1.04, 1.07)	5.00E-18	21%	1.08 (0.88, 1.14)	**3.89E-03**
[RTEL1]	rs6011033	20:62322699	G/A	78%	1.05 (1.03, 1.06)	3.50E-14	76%	1.09 (1.03, 1.14)	**1.14E-03**
[RUNX1]	rs73205303	21:36467830	A/G	13%	1.04 (1.03, 1.06)	7.90E-10	15%	1.15 (1.08, 1.22)	**8.30E-06**
[SIK1]	rs76081789*	21:44846426	T/C	94%	1.07 (1.04, 1.09)	1.30E-08	-	-	-
TEF-[]-TOB2	rs5758343	22:41816652	A/T	22%	1.05 (1.04, 1.06)	4.80E-14	20%	1.08 (0.88, 1.14)	**4.59E-03**

*rs76081789 was not available in our asthma study nor a suitable proxy.

Supplementary Table 5: Summary of stage 1 results for variants previously associated with allergic diseases in the Study of Zhu et. al 2018
35 signals with P<0.05 for moderate-severe asthma have their P value in bold; 28 signals had $P<1.32\times10^{-3}$ for moderate-severe asthma (Bonferroni correction for 38 tests).

				Asthma, Allergy and meta-analysis (Zhu et al. 2018)				Moderate-severe asthma (this study)		
**Nearest gene**	**rsid**	**Position (b37)**	**Effect/ other allele**	**Asthma P**	**Allergy P**	**Meta OR**	**Meta P**	**Effect allele frequency**	**OR (95% CI)**	**P**
HLA-DQB1	rs9273374	6:32626614	A/G	1.31E−35	1.17E−4	0.84	7.87E−35	39%	0.81 (0.77, 0.84)	**7.13E-22**
C11orf30	rs7936070	11:76293527	T/G	2.17E−21	4.40E−22	1.08	2.81E−28	47%	1.14 (1.1, 1.19)	**8.33E-10**
IL1RL1	rs72823641	2:102936159	A/T	7.43E−18	4.27E−24	0.89	1.58E−27	14%	0.84 (0.79, 0.9)	**5.25E-08**
SMAD3	rs56062135	15:67455630	T/C	2.62E−23	6.23E−13	1.16	1.56E−22	24%	1.18 (1.12, 1.24)	**8.31E-11**
IL33	rs9775039	9:6177453	A/G	7.41E−23	4.84E−8	1.18	4.42E−22	16%	1.22 (1.15, 1.29)	**7.38E-12**
CLEC16A	rs36045143	16:11224966	G/A	9.05E−17	8.94E−17	0.93	1.83E−21	25%	0.82 (0.78, 0.86)	**3.84E-15**
TSLP	rs1837253	5:110401872	T/C	1.81E−20	1.07E−13	0.93	4.38E−21	26%	0.81 (0.77, 0.85)	**2.81E-18**
SLC25A46	rs7705653	5:110142816	G/A	4.58E−10	1.88E−20	1.14	1.12E−19	15%	1.13 (1.06, 1.2)	**1.03E-04**
TLR10	rs28393318	4:38784267	G/A	2.61E−10	6.67E−20	0.92	2.14E−19	20%	0.92 (0.88, 0.97)	**2.83E-03**
GSDMB	rs869402	17:38068043	C/T	7.02E−18	6.70E−4	1.12	4.15E−17	48%	1.11 (1.07, 1.16)	**1.10E-06**
D2HGDH	rs34290285	2:242698640	A/G	3.89E−11	1.54E−15	0.93	5.17E−17	26%	0.82 (0.79, 0.87)	**5.79E-15**
LINC00299	rs10174949	2:8442248	A/G	6.08E−10	5.72E−16	0.94	1.70E−16	29%	0.9 (0.86, 0.95)	**1.55E-05**
SMARCE1	rs9911533	17:38775476	C/T	2.08E−07	1.65E−16	0.92	9.70E−16	37%	0.94 (0.9, 0.99)	**8.54E-03**
LOC101928272	rs12413578	10:9049253	T/C	3.27E−14	4.63E−10	0.91	1.09E−14	10%	0.79 (0.73, 0.84)	**1.20E-11**
IL7R	rs6881270	5:35879095	T/C	7.87E−6	2.61E−15	0.91	1.53E−14	28%	0.91 (0.87, 0.95)	**6.33E-05**
SUOX	rs10876864	12:56401085	G/A	1.43E−12	8.49E−10	1.05	1.41E−13	43%	1.13 (1.08, 1.18)	**2.18E-08**
STAT6	rs1059513	12:57489709	C/T	1.18E−11	1.62E−09	0.92	7.65E−13	11%	0.85 (0.79, 0.91)	**2.80E-06**
IL2	rs56267605	4:123363109	C/A	3.29E−10	6.59E−10	1.05	2.56E−12	33%	1.05 (1, 1.1)	**3.82E-02**
FLG-AS1	rs61816766	1:152319572	C/T	3.10E−8	3.07E−11	1.14	4.63E−12	4%	1.33 (1.18, 1.5)	**3.85E-06**
IL2RA	rs61839660	10:6094697	T/C	6.86E−4	3.98E−12	1.12	2.30E−11	10%	1.07 (1, 1.15)	5.39E-02
RAD50	rs2706362	5:131925187	C/T	1.27E−11	1.73E−7	1.06	3.75E−11	19%	1.13 (1.07, 1.19)	**8.43E-06**
CD247	rs1214598	1:167426424	A/G	6.66E−8	4.54E−10	0.95	5.14E−11	38%	0.88 (0.85, 0.92)	**3.05E-08**
LAYN	rs659529	11:111436896	T/A	3.45E−7	1.20E−10	0.95	6.03E−11	29%	0.92 (0.88, 0.97)	**8.29E-04**
LOC101927770	rs2766664	20:52171241	A/G	1.72E−3	1.41E−11	1.08	8.07E−11	24%	1.03 (0.98, 1.09)	1.98E-01
TPD52L3	rs2169282	9:6350235	A/G	3.13E−11	1.30E−5	1.09	1.80E−10	40%	1.12 (1.07, 1.17)	**4.06E-07**
KIF3A	rs10074523	5:132060583	C/A	4.15E−11	1.13E−5	1.1	2.37E−10	25%	1.1 (1.04, 1.15)	**2.69E-04**

				Asthma, Allergy and meta-analysis (Zhu et al. 2018)				Moderate-severe asthma (this study)		
**Nearest gene**	**rsid**	**Position (b37)**	**Effect/ other allele**	**Asthma P**	**Allergy P**	**Meta OR**	**Meta P**	**Effect allele frequency**	**OR (95% CI)**	**P**
SLC7A10	rs10414065	19:33721455	T/C	6.78E−9	2.74E−8	0.91	2.63E−10	6%	0.84 (0.77, 0.92)	**1.94E-04**
ABCB5	rs6461503	7:20560996	T/C	2.13E−9	8.16E−8	1.05	3.19E−10	48%	1.07 (1.02, 1.11)	**3.68E-03**
RTEL1	rs3208007	20:62322288	T/C	9.27E−9	2.81E−8	0.95	3.22E−10	24%	0.92 (0.88, 0.97)	**2.08E-03**
LINC00708	rs10795656	10:8595839	A/G	2.10E−6	3.19E−10	1.05	4.07E−10	27%	1.07 (1.02, 1.13)	**3.84E-03**
C5orf56	rs2548992	5:131808668	A/G	7.96E−11	5.40E−4	1.1	4.54E−10	26%	1.17 (1.11, 1.23)	**2.10E-10**
ZBTB10	rs2136016	8:81300681	G/A	3.72E−10	3.57E−4	1.09	2.11E−9	39%	1.13 (1.08, 1.18)	**6.01E-08**
IVL	rs61815704	1:152893891	G/C	4.08E−7	5.51E−8	1.14	5.16E−9	2%	1.3 (1.13, 1.5)	**2.38E-04**
RERE	rs301805	1:8481016	T/G	6.01E−8	3.36E−7	0.96	6.43E−9	42%	0.89 (0.86, 0.93)	**3.58E-07**
RUNX3	rs742230	1:25251424	G/A	5.95E−6	1.27E−8	0.96	1.02E−8	43%	0.93 (0.89, 0.97)	**1.06E-03**
RAD51B	rs8008961	14:68752643	T/C	2.14E−7	3.01E−7	1.05	1.24E−8	28%	1.06 (1.01, 1.11)	**1.83E-02**
NRROS	rs4916533	3:196373582	T/C	2.79E−7	3.62E−7	0.93	1.66E−8	8%	0.88 (0.81, 0.95)	**1.12E-03**
EVI5	rs12743520	1:93037112	A/C	3.02E−3	6.87E−9	0.93	3.83E−8	26%	0.96 (0.91, 1)	7.06E-02

Supplementary Table 6: Summary of results for 3 novel signals for moderate-severe asthma in the 23&Me asthma GWAS

Chr	Position	Variant	Locus	Non-coded	coded	Minor allele	MAF	Meta-analyses		23&Me				
								**OR [95% CI]**	**P**	**rsid**	**proxy r2**	**Alleles (A/B)**	**OR [95% CI]**	**P**
4	123,055,701	rs560026225	*KIAA1109*	G	GATT	GATT	23.60%	1.12 [1.08-1.16]	3.06E-09	rs72687036	0.66	A/G	1.06 [1.04-1.08]	3.96E-07
10	8,115,362	rs10905284	*GATA3*	C	A	C	42.94%	0.90 [0.88-0.93]	1.76E-10	rs10905284	1.00	A/C	1.04 [1.02-1.06]	2.72E-05
11	1,136,478	rs11603634	*MUC5AC*	A	G	A	49.64%	1.09 [1.06-1.12]	2.32E-08	rs11603634	1.00	A/G	1.00 [0.97-1.02]	8.09E-01

The corresponding effect size (OR) defined as per copy of the B allele.

Supplementary Table 7: GRASP and GWAS catalog look up for variants in LD ($r^2 > 0.4$) with 3 novel signals for moderate-severe asthma

**Gene**	**Sentinel**	**Reported genes**	**Reported SNP**	**Band.**	**r^2**	**phenotype**	**p-value**	**N**	**First author/ Journal**	**PMID**	**Year**
KIAA1109	rs560026225	*KIAA1109*	rs45515895	4q27	0.562	Mean platelet volume	8.00E-10	164,454	Astle WJ, *Cell*	27863252	2017
KIAA1109	rs560026225	*IL2, ADAD1, KIAA1109, IL21*	rs17454584	4q27	0.573	Allergic sensitization	6.00E-10	31,879	Bonnelykke K, *Nat. Genet.*	23817571	2014
GATA3	rs10905284	*FLJ45983*	rs7897792	10p14	0.808	Rheumatoid arthritis	1.90E-10	58,284	Okada Y, *Nature*	24390342	2014
GATA3	rs10905284	*GATA3*	rs10905284	10p14	1.000	Eosinophil counts	7.00E-09	172,275	Astle WJ, *Cell*	27863252	2017
MUC5AC	rs11603634	-	rs4077759	11p15.5	0.416	Fibrotic idiopathic interstitial pneumonias (pulmonary fibrosis)	8.47E-13	50,410	Fingerlin TE, *Nat. Genet.*	23583980	2013

Supplementary Table 8: Results of eQTL analyses for variants meeting genome wide significance ($P < 5 \times 10^{-8}$) with susceptibility to moderate-severe asthma.

Chr	Position	Variant	Locus	Sentinel SNP	Proxy	No. SNPs	R2	eQTL P	Probeset id	Gene	Effect allele	Direction	Tissue	Dataset
1	167,427,247	rs7523907	*CD247*	rs7523907	n/a	1	1	8.00E-15	6290400	*CD247*	C	+	Blood	Westra-cis-eQTL
2	102,949,161	rs12479210	*IL1RL1*	rs12479210	n/a	168	1	6.20E-10	100136977_TGI_at	*IL18R1*	C	+	Lung, Blood	UBio-cis-eQTL (2), UBC-cis-eQTL
					n/a	843	1	8.70E-35	100148210_TGI_at, 100302151_TGI_at, 100148162_TGI_at, 100302783_TGI_at, 100312840_TGI_at	*IL1RL1*	C	+	Lung	UBC-cis-eQTL
					rs6543114	1	0.681875	1.82E-05	100129736_TGI_at	*SLC9A2*	A	+	Lung	UBC-cis-eQTL
2	242,698,640	rs34290285	*D2HGDH*	rs34290285	n/a	31	1	0.000001	100154978_TGI_at	*D2HGDH*	G	+	Lung	UBC-cis-eQTL
					n/a	26	1	2.64E-08	100135940_TGI_at	*ING5*	G	+	Lung	UBC-cis-eQTL
4	123,055,701	rs560026225	*KIAA1109*	4:123055701:G:GATT (rs560026225)	rs17454584	3	0.573326	1.51E-05	100144991_TGI_at	*KIAA1109*	A	+	Lung	UBC-cis-eQTL
5	110,467,499	rs1438673	*WDR36*	rs1438673	rs2289277	2	0.745315	1.73E-05	100142361_TGI_at	*TSLP*	C	+	Lung	UBC-cis-eQTL
5	131,799,626	rs3749833	*C5orf56*	rs3749833	rs2548992	72	0.970592	0.00000494	100132153_TGI_at	*RAD50*	G	-	Lung	UBC-cis-eQTL
					n/a	41	1	2.84E-10	100123073_TGI_at	*SLC22A5*	T	-	Lung	UBC-cis-eQTL
					n/a	37	1	1.91E-08	100150988_TGI_at	*U46120*	T	-	Lung	UBio-cis-eQTL
5	131,887,986	rs1986009	*RAD50*	rs1986009	rs12652920	5	0.984912	0.0000139	100158314_TGI_at	*BC043424*	G	+	Lung	UBC-cis-eQTL
					n/a	72	1	0.00000597	100132153_TGI_at	*RAD50*	C	-	Lung	UBC-cis-eQTL
					rs12652920	29	0.984912	1.75E-13	100158314_TGI_at	*SLC22A5*	G	+	Lung, Blood	Westra-cis-eQTL, UBC-cis-eQTL
6	32,581,739	rs776111176	*HLA-DQA1*	chr6:32581739 (rs776111176)	rs2395175	2	0.501612	8.89E-11	100304000_TGI_at	*PSORS1C3*	G	-	Lung	UBC-trans-eQTL
					rs2760982	1	0.448833	1.24E-12	100134503_TGI_at	*ENST00000342748*	G	-	Lung	UBC-trans-eQTL
6	32,627,250	rs9273410	*HLA-DQB1*	rs9273410	rs9274607	10	0.838508	9.83E-19	100126665_TGI_at, 100135327_TGI_at	*AGPAT1*	G	-	Lung	UBC-cis-eQTL
					rs9271383	2	0.638051	3.41E-06	100139818_TGI_at	*BAT2*	C	+	Lung	UBC-cis-eQTL
					rs9274607	28	0.838508	7.10E-11	100158347_TGI_at	*C19orf6*	C	-	Lung	UBC-trans-eQTL
					rs9271383	140	0.638051	5.63E-07	100161395_TGI_at, 100129037_TGI_at	*TAP2*	C	+	Lung	UBC-cis-eQTL
					rs3129758	32	0.584267	7.43E-11	100303039_TGI_at	*CDSN*	G	-	Lung	UBC-trans-eQTL

					rs9273527	2	0.618033	1.72E-10	100161253_TGI_at	*BTN3A2*	T	+	Lung	UBC-trans-eQTL
					rs9273527	4	0.618033	6.72E-11	100303994_TGI_at	*ZFP57*	T	_	Lung	UBC-trans-eQTL
					rs72844114	2	0.457473	3.67E-11	100306864_TGI_at	*ZNF764*	G	+	Lung	UBC-trans-eQTL
					rs72853984	3	0.459233	1.45E-10	100134503_TGI_at	*ENST00000342748*	T	+	Lung	UBC-trans-eQTL
					rs9273481	4	0.620469	1.51E-10	100129349_TGI_at	*HLA-A*	G	+	Lung	UBC-trans-eQTL
					rs9274607	6	0.838508	8.36E-80	100313766_TGI_at	*HLA-DQA2*	C	+	Lung	UBC-cis-eQTL
					n/a	1058	1	9.36E-14	209480_at, 100311704_TGI_at, 100300398_TGI_at	*HLA-DQB1*	A	-	Nasal Brush, Bronchial Brush, Blood, Lung, Sputum	UBio-cis-eQTL, UBC-cis-eQTL
					rs9274607	112	0.838508	0.00000372	100311684_TGI_at	*HLA-DQB2*	C	-	Lung	UBC-cis-eQTL
					rs9274607	234	0.838508	1.35E-19	100125275_TGI_at	*HLA-DRB1*	C	-	Lung	UBC-cis-eQTL
					rs9273241	134	0.831772	4.05E-24	100139572_TGI_at	*HLA-DRB3*	C	+	Lung	UBC-trans-eQTL
					rs9274607	135	0.838508	2.29E-73	100127159_TGI_at	*HLA-DRB4*	C	+	Lung	UBC-trans-eQTL
					rs9274607	154	0.838508	7.74E-21	100302940_TGI_at	*HLA-DRB5*	C	-	Lung	UBC-cis-eQTL
					rs9274607	138	0.838508	5.50E-40	100160430_TGI_at	*HLA-DRB6*	C	+	Lung	UBC-cis-eQTL
11	1,136,478	rs11603634	*MUC5AC*	rs11603634	rs11602802	4	0.460739	2.50E-05	214303_x_at	*MUC5AC*	A	+	Bronchial brush	UBio-cis-eQTL
11	76,293,726	rs7936312	*C11orf30*	rs7936312	n/a	15	1	4.48E-13	100143158_TGI_at	*C11orf30*	G	+	Lung	UBC-cis-eQTL
12	56,449,875	rs7305461	*RPS26*	rs7305461	rs1081975	55	0.917454	2.61E-242	100146882_TGI_at	*40S ribosomal protein S26 pseudogene*	C	-	Lung	UBC-trans-eQTL
					rs705700	43	0.894132	0.0000166	100146593_TGI_at	*CDK2*	T	-	Lung	UBC-cis-eQTL
					rs2271194	1	0.930264	0.000119964	1510564	*CNPY2*	A	-	Blood	Westra-cis-eQTL
					rs1131017	55	0.981593	3.68E-43	100156286_TGI_at	*ENST00000299756*	G	-	Lung	UBC-trans-eQTL
					rs1131017	55	0.981593	1.50E-240	100131680_TGI_at	*ENST00000356247*	G	-	Lung	UBC-trans-eQTL
					rs1131017	7	0.981593	0.00000201	100144439_TGI_at	*FAM62A*	A	-	Lung	UBio-cis-eQTL, UBC-cis-eQTL
					rs1131017	8	0.981593	2.27E-10	100129882_TGI_at	*hCT32692*	G	-	Lung	UBC-trans-eQTL
					n/a	1	1	0.001296289	1090433	*RNF41*	A	+	Blood	Westra-cis-eQTL
					rs1131017	55	0.981593	6.98E-190	100312533_TGI_at	*similar to 40S ribosomal protein S26*	G	-	Lung	UBC-cis-eQTL

12	57,497,005	rs703816	*STAT6*	rs703816	n/a	91	1	5.12E-36	100310285_TGI_at, 100156721_TGI_at, 100305452_TGI_at	*STAT6*	T	-	Lung, Blood	Westra-cis-eQTL, UBC-cis-eQTL
15	67,441,750	rs72743461	*SMAD3*	rs72743461	rs17228058	1	0.995669	4.68E-06	6840328	*SMAD3*	G	-	Blood	Westa-cis-eQTL
16	11,230,703	rs7203459	*CLEC16A*	rs7203459	n/a	14	1	6.10E-09	100143389_TGI_at	*AK124470*	T	+	Lung	UBC-cis-eQTL
					rs34540843	1	0.968229	0.000390341	231221_at	*CLEC16A*	G	+	Blood	UBio-cis-eQTL
					rs2241099	5	0.963908	2.60E-40	1990300	*DEXI*	G	-	Blood	Westra-cis-eQTL
17	37,910,368	rs2941522	*IKZF3*	rs2941522	n/a	536	1	3.28E-11	100307813_TGI_at, 100152237_TGI_at, 100305614_TGI_at, 100307813_TGI_at	*CRKRS*	T	-	Lung	UBC-cis-eQTL
					n/a	211	1	2.43E-18	100138145_TGI_at	*GSDMA*	T	-	Lung	UBC-cis-eQTL
					n/a	211	1	1.00E-14	100144966_TGI_at, 219233_s_at	*GSDMB*	T	+	Lung, Blood	Westra-cis-eQTl, UBC-cis-eQTL
					rs907091	88	0.97553	0.0000122	100127152_TGI_at	*ORMDL3*	C	-	Lung	UBC-cis-eQTL
					rs903506	64	0.471	4.48E-10	100145295_TGI_at	*PERLD1*	G	+	Lung	UBC-cis-eQTL
					rs2952140	4	0.963009	0.000015	100134069_TGI_at	*PLXDC1*	C	-	Lung	UBC-cis-eQTL
					rs876493	1	0.3443	3.43E-07	100125568_TGI_at	*PNMT*	G	+	Lung	UBC-cis-eQTL
					rs36095411	5	0.865581	1.49E-10	100126839_TGI_at	*ZPBP2*	T	-	Lung	UBC-trans-eQTL
17	47,439,302	rs112502960	*ZNF652*	rs112502960	rs4794029	7	0.480575	1.72E-08	100124455_TGI_at	*GNGT2*	C	-	Lung	UBC-cis-eQTL

- UBC-cis-eQTL – University of British Columbia Lung eQTL dataset[10] (10% FDR)
- Westra – Blood cell dataset[9] (10% FDR)
- UBio – U-BIOPRED datasets from blood cells, sputum, lung biopsy, bronchial brushing and nasal brushing[12,13] (5% FDR).
- No. SNPs – number of SNPs identified in LD (r2<0.4) with sentinel SNP and showing association at designated FDR.
- P value reported corresponds to lowest p-value for any SNP/probe combination and R2 is the linkage disequilibrium between this reported SNP and the sentinel SNP from the case-control association results.

Supplementary Table 9: ENCODE analyses of rs11603634 (shown in red) and proxies (r^2>0.4) highlighting potential regulatory mechanisms underlying eQTL for *MUC5AC*

**SNP**	**LD (R^2)**	**Promoter histone marks**	**Enhancer histone marks**	**DNAse**	**Proteins bound**	**Motifs changed**
rs11602802	0.46	-	ESDR, LNG, MUS, KID, GI, BRN, BONE	LNG,BRST,SKIN,MUS,BRN,LNG	-	NF-Y
rs11600289	0.45	-	LNG, ADRL, GI	-	-	HDAC2,PRDM1
rs12791023	0.45	-	IPSC, GI	-	-	LBP-1,LBP-9
rs11603634	1	-	GI	GI,MUS,SKIN		Fox,Foxa,Foxc1,Foxd3,Foxj1,Foxk1,HDAC2,PPAR,Zfp105,p300
rs10902099	1	-	-	-	-	Evi1,Foxo,Foxp1,GATA,HDAC2,HNF1,Hoxa10,TATA,Zfp105
rs10902102	0.44	-	-	-	-	HMG-IY,Hoxa9,Mef2,Nkx6-1,Pou2f2,Zfp105
rs11245979	0.45	-	-	-	-	CTCF,NF-kappaB
rs28490880	0.4	LNG, CRVX	ESC, ESDR, LNG, FAT, STRM, BLD, MUS, SKIN, GI, LIV, BRN, BONE	ESDR,BLD,BLD,BLD,BLD,GI,LNG,BLD,CRVX,BRN	ERALPHA_A,NFKB,PU1,AP2GAMMA,FOSL2,ZBTB7A	Ets,GR,Hic1,NF-kappaB,Roaz,SREBP,Znf143

Data from HaploReg v4.1 (http://archive.broadinstitute.org/mammals/haploreg/haploreg.php)

Supplementary Table 10: ENCODE analyses of rs560026225 (proxy rs72687036 shown in red) and proxies (r^2>0.4) highlighting potential regulatory mechanisms underlying eQTL for *KIA1109*.

SNP	LD (R^2)	Promoter histone marks	Enhancer histone marks	DNAse	Proteins bound	Motifs changed
rs17518038	0.42	-	-	-	-	Pou2f2,STAT
rs3108402	0.41	-	-	-	-	HDAC2
rs3108401	0.42	-	GI	-	-	Bcl6b,Ehf,Elf3,Irf,STAT,Tel2
rs1949945	0.41	-	-	-	-	-
rs941130	0.41	-	ESDR, LNG	-	-	-
rs10021288	0.69	-	-	-	-	-
rs4279237	0.74	-	LNG	-	-	-
rs3115230	0.45	-	-	-	-	Foxo,Mef2
rs76080510	0.59		FAT, LNG, SKIN	-	-	Bcl6b,CEBPG,Gfi1,Hoxc9,Lhx3,Ncx,Nkx6-1,OTX,Pax-6,Pou3f4,STAT
rs77130822	0.58		FAT, LNG, SKIN	BRN,BRN,LNG,GI,SKIN	-	Ets,Pax-5,Pax-6,STAT
rs6534338	0.48	-	-	-	-	INSM1,Maf,Pou2f2,Pou5f1,YY1,ZBTB7A
rs114785709	0.72	BLD	ESC, BLD	-	-	AP-2,LF-A1,PU.1,Rad21,SP1,SREBP,Zbtb12
rs56035021	0.55	-	-	-	-	Foxd1,NRSF
rs112481526	0.68	-	BLD	-	-	EBF,Pax-5
rs62323898	0.54	-	BLD	-	-	-
rs10857090	0.51	-	-	-	-	-
rs139735807	0.94	-	-	-	-	Arid5b,ERalpha-a,GCNF,HDAC2,HEY1,MZF1::1-4,NR4A,Nkx2,Pou1f1,RORalpha1,SF1
rs72687029	0.95	-	-	-	-	Arid3a,Mef2,Myb,TATA
rs55969942	0.56	-	VAS	-	-	Hsf,Pou2f2
rs55904957	0.56	-	VAS	-	CFOS	AFP1,AIRE,Pax-4
rs7671357	0.56	-	-	-	-	-
rs4505848	0.55		FAT	-	-	Mef2,Myc
rs5861560	0.57	BLD	BLD	-	-	Barx1,DMRT2,DMRT4,Foxa,HNF1,PLZF,Pou2f2,TATA
rs45605540	0.73	-	-	-	-	CCNT2,GATA,Hand1,LF-A1,TAL1
rs7664452	0.58	-	-	-	-	E4BP4,Foxk1,Foxo,Foxp3,HDAC2,STAT
rs72687036	1	-	STRM, HRT	-	-	-
rs143502740	0.96	-	-	-	-	Cdx2,Hoxa10,Hoxd10,Nanog,Pou1f1,p300

rs202035542	0.44	-	-	-	-	Nanog,Pou1f1
rs6827756	0.49	-	BLD	-	-	Arid3a,Mef2,Pou2f2,p300
rs45515895	0.99	-	BLD	-	-	-
rs72687044	0.57	LNG	-	-	-	HES1,TATA
rs13147049	0.57	-	-	-	-	Nkx6-1,Pdx1,Pou3f4,Sp100
rs6534347	0.49	-	-	-	-	AIRE,ERalpha-a
rs28854492	0.55	-	-	MUS	-	EBF,MZF1::1-4,Sin3Ak-20
rs144914346	0.95	-	-	-	-	Arid5b,Rad21,Smad
rs34834002	0.54	-	-	-	-	Barx1,Bsx,Dlx2,Egr-1,Ets,GATA,Hoxa5,LUN-1,NERF1a,STAT
rs7693745	0.7	-	-	-	-	CTCFL,GATA,HNF4
rs200269026	0.47	-	-	FAT, STRM, BLD	-	
rs7699742	0.53	-	-	-	-	CEBPG,GATA,HEY1,Myc,Smad3,TATA,p53
rs11722421	0.54	-	-	-	-	-
rs45528737	0.44	-	-	-	-	E2F
rs1127348	0.9	-	-	-	-	Brachyury,Zbtb12
rs201757131	0.5	-	-	-	-	Barhl1,Barx2,CDP,Dbx1,Dbx2,En-1,Esx1,Foxd1,Foxp1,HMG-IY,HNF1,Hlx1,Hmx,Hoxa10,Hoxa3,Hoxa7,Hoxb4,Hoxc6,LUN-1,Lhx3,Mef2,Msx-1,Nkx2,OTX,Pax-4,Pax-6,Pax7,Phox2a,Pou2f2,Pou3f2,Prrx1,Prrx2,Vax2,Zfp105
rs33976078	0.54	-	-	-	-	Dbx1,Foxd1,Foxp1,Foxq1,HMG-IY,Hoxa10,Mef2,OTX,Pax-4,Zfp105
rs11727369	0.47	ESC, IPSC, PLCNT	ESC, ESDR, IPSC, BLD, SKIN, LIV	-	YY1,MAFF,MAFK	DBP,Znf143
rs56313700	0.9	-	-	-	-	PPAR,THAP1
rs151275015	0.43	-	-	-	-	NF-kappaB,PLZF
rs1383046	0.88	-	-	-	-	-
rs4145717	0.53	-	-	-	-	Hoxa9,Pbx-1
rs17388568	0.66	-	-	-	-	CDP,Cdc5,Pou2f2,RFX5
rs12499753	0.52	-	-	-	-	-
rs1904522	0.51	-	-	-	-	DMRT5,PLZF
rs55896948	0.51	-	-	-	-	-
rs716501	0.51	-	-	-	-	GR
rs17454584	0.86	-	-	-	-	Mrg1::Hoxa9,Pou2f2,Pou5f1

rs35914000	0.64	-	BLD	-	-	Irf
rs56360035	0.51	-	-	-	-	HDAC2,LXR,PRDM1,Pax-4,SP1,UF1H3BETA
rs56267605	0.51					ELF1,Ets,Irf,Nkx3,PU.1,Pax-8
rs3136534	0.51					DMRT2,Pou2f2,TCF4
rs2069772	0.63	BLD				Arid3a,Barx2,Dbx2,En-1,Evi-1,Gbx1,Gbx2,HNF1,Hlxb9,Hoxa4,Hoxa7,Hoxb4,Hoxb7,Hoxc6,Hoxd8,Msx-1,Msx2,Ncx,Nkx6-1,Nkx6-2,Nobox,Pax-6,Pax7,Phox2a,Pou2f2,Pou3f2,Pou3f3,Pou3f4,Pou4f3,Pou6f1,Prrx1,Sox
rs2069763	0.51	BLD, GI	BLD	THYM		Hand1,Msx-1
rs58939053	0.49					
rs45599938	0.5					CTCF,Pax-5,Rad21
rs11098659	0.51					Bbx,En-1,Foxp1,Gfi1b,Hdx,Msx-1,YY1
rs45454992	0.62					NF-I,TLX1::NFIC
rs45610037	0.84		FAT, LNG, BLD	BLD,SKIN		AP-1,CEBPB,Sox,Zec

Data from HaploReg v4.1 (http://archive.broadinstitute.org/mammals/haploreg/haploreg.php)

Supplementary Table 11: ENCODE analyses of rs10905284 (*GATA3,* shown in red) and proxies (r^2<0.4) highlighting potential mechanisms

SNP	LD (R^2)	Promoter histone marks	Enhancer histone marks	DNAse	Proteins bound	Motifs changed
rs2275806	0.41	ESC, ESDR, IPSC, FAT, BRST, BLD, MUS, BRN, SKIN, LIV, PLCNT, GI, HRT, CRVX, VAS	ESC, ESDR, IPSC, FAT, BLD, BRN, SKIN, GI, LNG, MUS, THYM, HRT, PANC, SPLN	ESC,ESDR,ESDR,ESDR,ESDR,ESC,IPSC,IPSC, BRST,BLD,BLD,BLD,BLD,BLD,BLD,SKIN,ADRL,BRN,HRT,KID,LNG,MUS,MUS,PLCNT,GI, THYM,MUS,GI,LNG,BLD,LIV,BRST,MUS,VAS,BLD,SKIN	CTCF,RAD21,SMC3,YY1,ZNF143,POL2,CTCFL,HAE2F1	GR,ZBRK1
rs3781094	0.55	BRST, BLD, SKIN, FAT, ADRL, KID, VAS	ESC, ESDR, IPSC, BLD, MUS, BRN, SKIN, LNG, HRT, SPLN	ESC,ESDR,ESDR,ESDR,ESDR,ESC,IPSC,IPSC, BRST,BLD,BLD,BLD,BLD,SKIN,ADRL,BRN,LNG,GI,THYM,BRST,VAS,BLD		Hbp1,Pou2f2,TCF12
rs3802604	0.58	ESC, ESDR, FAT, BRST, BLD, MUS, SKIN, ADRL, KID, LNG, GI, THYM, VAS, BRN	ESC, ESDR, IPSC, FAT, BLD, BRN, SKIN, LNG, MUS, GI	ESC,ESDR,ESDR,ESDR,ESDR,ESC,LNG,IPSC, BRST,BLD,BLD,BLD,BLD,BLD,SKIN,SKIN,SKIN,ADRL,BRN,KID,LNG,MUS,PLCNT,GI,THYM,GI,BLD,BRST,MUS,MUS,VAS,BRN,SKIN, LNG	BATF,EBF1,POL2,SIX5,NFKB,P300,RAD21, ZNF263	BDP1,ERalpha-a,EWSR1-FLI1,GR,NRSF,PU.1,Rad21,SMC3,Spz1,TATA,WT1,ZNF263,Znf143
rs477771	0.48	ESDR, BLD, KID, GI, THYM, BRST, VAS, SKIN	ESC, ESDR, IPSC, BRST, BLD, FAT, GI, ADRL, MUS, BRN	ESDR,ESC,IPSC,BRST,BLD,BLD,SKIN,THYM, BLD	EBF1,NFKB	PLAG1,TATA,p300
rs444762	0.5	ESDR, BLD, KID, GI, THYM, BRST, VAS, SKIN	ESC, ESDR, IPSC, BRST, BLD, FAT, ADRL, MUS, SPLN, BRN	ESC,ESDR,ESDR,ESDR,ESC,IPSC,BRST,BLD, BLD,BLD,SKIN,PLCNT,THYM,BLD,BRST,VAS,SKIN	POL2	
rs376397	0.5	BLD, KID, GI, THYM, BRST, VAS	ESC, ESDR, IPSC, BRST, BLD, FAT, ADRL, MUS, SPLN, BRN	ESC,ESDR,ESDR,ESDR,IPSC,BRST,BLD,BLD, BLD,SKIN,ADRL,PLCNT,THYM,BLD,BRST,VAS,SKIN	POL2	GATA
rs409560	0.49	BLD, ADRL, KID, GI, BRST, VAS	ESC, ESDR, IPSC, BRST, BLD, FAT, SPLN, BRN	SDR,ESDR,ESDR,ESDR,ESC,IPSC,BLD,BLD,SKIN,PLCNT,THYM,BLD,VAS,SKIN		HEY1,Mrg,Nkx2,TATA
rs374641	0.63	BLD, BRST	ESC, ESDR, IPSC, BRST, BLD, FAT, LNG, PLCNT, SPLN, BRN	ESDR,BRST,BLD,BLD,PLCNT,VAS,SKIN,LNG	EGR1,HAE2F1,POL2	CTCF,NRSF,Pbx3,SMC3
rs386680	0.64	BLD, BRST	ESC, ESDR, IPSC, BRST, BLD, SKIN, FAT, THYM, SPLN, BRN	BRST,BLD,BLD,PLCNT,VAS,BLD,SKIN,LNG	EGR1,HAE2F1,POL2	BCL,CTCF,NRSF,Sin3Ak-20
rs371668	0.64	BLD, BRST	ESC, ESDR, IPSC, BRST, BLD, FAT, ADRL, PLCNT, SPLN, VAS, BRN	ESDR,ESDR,BRST,BLD,BLD,BLD,BLD,PLCNT,THYM,VAS,BLD,BRN,SKIN,LNG	EGR1,HAE2F1,POL2	EBF,HEY1,LBP-1,LBP-9,NRSF,Pbx3,Rad21,p300
rs3824660	0.64	ESDR, BRST, KID, VAS	ESC, ESDR, IPSC, BRST, BLD, SKIN, FAT, THYM, SPLN, BRN	PLCNT		BCL,BDP1,NF-kappaB,YY1
rs2277228	0.7	ESC, ESDR, KID, PLCNT	ESDR, IPSC, ESC, BRST, BLD, MUS, SKIN, FAT, ADRL, PLCNT, THYM, HRT, LNG, SPLN	ESC,ESDR,ESDR,ESDR,ESC,IPSC,IPSC,PLCNT,THYM,VAS,SKIN		CDP,PLAG1
rs201042786	0.7	ESC, KID	ESC, ESDR, IPSC, BRST, BLD, SKIN, FAT, ADRL, PLCNT, THYM, MUS		GATA2	EWSR1-FLI1,Evi-1,Irf
rs570730	0.61	ESC, KID	ESC, ESDR, IPSC, BRST, BLD, SKIN, FAT, LIV, BRN, ADRL, PLCNT, MUS, VAS	ESC,BLD,MUS,VAS,SKIN		Gm397,Nkx2,Zfp691

rs570613	0.61	ESC, KID	ESC, ESDR, IPSC, BRST, BLD, SKIN, FAT, LIV, BRN, ADRL, PLCNT, MUS, VAS	ESDR,ESDR,ESC,IPSC,IPSC,MUS,VAS,SKIN		GR,Pbx3,SETDB1
rs568727	0.61	ESC, KID	ESC, ESDR, IPSC, BRST, BLD, SKIN, FAT, ADRL, PLCNT, VAS	ESC,IPSC,IPSC,BRST,BLD,ADRL,BRST,VAS,SKIN	POL2	Maf,Nr2f2
rs10752126	0.7	-		ESC,ESC,IPSC,IPSC,BLD,ADRL,THYM,VAS	POL2	
rs10905279	0.69	-		BLD,GI,MUS,VAS,SKIN	GATA2,GABP,POL2	CEBPA
rs7897792	0.74	-	ESC, ESDR, IPSC, BRST, BLD, SKIN, FAT, THYM	VAS,SKIN	POL2	Foxp1,GATA,HDAC2,Pax-5,TATA,p300
rs537544	0.62	-	ESC, ESDR, IPSC, BRST, BLD, SKIN, FAT, THYM	VAS		GR,Gfi1,Irf
rs3847417	0.81	-	BLD			GATA,GR,ZID
rs3802597	0.98	-	ESDR, ESC, IPSC, BRST, BLD, THYM, PLCNT, HRT, VAS	ESC,ESDR,ADRL,MUS,BRN	CMYC,POL2	EBF,THAP1,YY1
rs10905284	1	-		ESDR,THYM,VAS		DMRT2,YY1
rs34610379	0.67	-		PLCNT,SKIN		Dbx1,TATA

Data from HaploReg v4.1 (http://archive.broadinstitute.org/mammals/haploreg/haploreg.php)

Supplementary Table 12: DeepSEA analyses of rs11603634 (*MUC5AC*) and proxies (r^2>0.4) identifies potential alterations in Fox transcription factor activity for rs11603634 and alterations in several factors for rs12788104.

(A) rs11603634

**Chromatin feature**	**Cell Type**	**Treatment**	**Log2 fold change**	**E-Value**
FOXA1	HepG2	None	1.6612	0.001328
FOXA2	HepG2	None	1.6506	0.001216
FOXA1	HepG2	None	1.643	0.001216
FOXA1	T-47D	DMSO_0.02pct	1.5325	0.000406
DNase	LNCaP	androgen	1.348	0.006265

(B) rs12788104 (r2 0.47 with rs11603634)

**Chromatin feature**	**Cell Type**	**Treatment**	**Log2 fold change**	**E-Value**
GATA2	K562	None	-5.2408	0.0001
TAL1	K562	None	-4.762	0.000089
GATA2	K562	None	-4.7	0.000026
GATA1	K562	None	-4.4207	0.000009
p300	K562	None	-4.1833	0.000545
ZEB1	GM12878	None	1.8451	0.000787

*E is short for 'Expect', and E-value is defined as the expected proportion of SNPs with larger predicted effect (from reference allele to alternative allele) for this chromatin feature. The predicted effect magnitude is measured as the product of relative and absolute change, i.e. |log(p_ref/(1-p_ref))-log(p_alt/(1-p_alt))| * |p_ref-p_alt| . E-value is computed based on the empirical distributions of predicted effects for 1000 Genomes SNPs.*

Supplementary Table 13: DeepSEA analyses of rs10905284 (*GATA3*) and proxies (r^2>0.4) identifies potential functional effects for rs10905284 and rs3802597.

(A) rs10905284

Chromatin feature	Cell Type	Treatment	Log2 fold change	E-Value
MafK	HepG2	None	0.80585	0.027291
MafF	HepG2	None	0.77694	0.026661
MafK	HepG2	None	0.77479	0.024518
MafK	IMR90	None	0.70611	0.027451
MafK	K562	None	0.61198	0.024304

(B) rs3802597 (r2 0.93 with rs10905284)

Chromatin feature	Cell Type	Treatment	Log2 fold change	E-Value
Max	HUVEC	None	2.0529	0.0028
USF1	SK-N-SH_RA	None	1.9338	0.0061
USF2	HepG2	None	1.8769	0.0084
USF1	A549	EtOH_0.02pct	1.8565	0.0064
USF1	HepG2	None	1.8417	0.0037

*E is short for 'Expect', and E-value is defined as the expected proportion of SNPs with larger predicted effect (from reference allele to alternative allele) for this chromatin feature. The predicted effect magnitude is measured as the product of relative and absolute change, i.e. |log(p_ref/(1-p_ref))-log(p_alt/(1-p_alt))| * |p_ref-p_alt| . E-value is computed based on the empirical distributions of predicted effects for 1000 Genomes SNPs.*

Supplementary Table 14: DeepSEA analyses of rs560026225 (*KIAA1109*) and proxies (r^2>0.4) identifies potential functional effects for rs17389644.

(A) rs17389644 (r2 0.57 with rs560026225)

Chromatin feature	Cell Type	Treatment	Log2 fold change	E-Value
DNase	HMVEC-dBl-Ad	None	1.9371	0.000929
DNase	HPAEC	None	1.8865	0.000845
DNase	HMVEC-dLy-Neo	None	1.8511	0.000955
DNase	HMVEC-LBl	None	1.8486	0.001183
Pol2-4H8	HUVEC	None	1.8327	0.000344

*E is short for 'Expect', and E-value is defined as the expected proportion of SNPs with larger predicted effect (from reference allele to alternative allele) for this chromatin feature. The predicted effect magnitude is measured as the product of relative and absolute change, i.e. |log(p_ref/(1-p_ref))-log(p_alt/(1-p_alt))| * |p_ref-p_alt| . E-value is computed based on the empirical distributions of predicted effects for 1000 Genomes SNPs.*
